# Supplementary material for: Synergy of dual-atom catalysts deviated from the scaling relationship for oxygen evolution reaction
Source: Nat Commun. 2023 Jul 24;14:4449. doi: 10.1038/s41467-023-40177-1 (PMC10366111; doi:10.1038/s41467-023-40177-1)
Supplement: Supplementary file 1 — Supplementary information [file 41467_2023_40177_MOESM1_ESM.docx]

Supplementary information

Synergy of Dual-atom Catalysts Deviated from the Scaling Relationship for Oxygen Evolution Reaction

Cong Fang^1,2^, Jian Zhou^1,2,3^, Lili Zhang^1,2,3^, Wenchao Wan^4^, Yuxiao Ding,^3,5^* and Xiaoyan Sun^1,2,3^*

^1^ Qingdao Institute of Bioenergy and Bioprocess Technology, Chinese Academy of Sciences, Qingdao 266101, China

^2^ Shandong Energy Institute, Qingdao 266101, China

^3^ University of Chinese Academy of Sciences, Beijing 100049, China

^4^ Max-Plank Institute for Chemical Energy Conversion, Mülheim an der Ruhr 45470, Germany

^5^ Lanzhou Institute of Chemical Physics, Chinese Academy of Sciences, Lanzhou 730000, China

E-mail: [sunxy@qibebt.ac.cn](mailto:sunxy@qibebt.ac.cn); [yuxiaoding@licp.cas.cn](mailto:yuxiaoding@licp.cas.cn);

# Table of Contents

1. **Figures**

***Figure S1***. Charge density differences of FeZr@NC and FePd@NC.

***Figure S2***. Optimized structures of oxygenated intermediates on M′M@NC surface.

***Figure S3***. Optimized structures of oxygenated intermediates on M@NC surface.

***Figure S4***. Gibbs free energy diagram of AEM and OCM for comparison.

***Figure S5***. Selectivity of the second *OH adsorption behaviour.

***Figure S6***. *O_2_ bond length versus acquired charge and Δ*G*_*O2→O2(g)_, respectively.

***Figure S7***. Optimized structure of M′M@Gr monolayer in a *p*(6 × 6) supercell.

***Figure S8***. The computed *η* of the FeM, CoM, NiM, and CuM dimers on M′M@Gr.

***Figure S9***. Gibbs free energy diagram along the OCM of M′M@Gr.

***Figure S10***. Scaling relations for *O_2_/*OH in terms of adsorption free energy (Δ*G*).

***Figure S11***. Relationship between Δ*G*_*OH_ and Δ*G*_*OOH_ for AEM.

***Figure S12***. Relationship between Δ*G*_*OH_ and Δ*G*_*OH-*O_ for M′M@Gr.

***Figure S13***. Gibbs free energy diagrams on M_2_@NC.

***Figure S14***. Optimized structures of oxygenated intermediates on M_2_@NC surface.

***Figure S15***. Relationship between Δ*G*_*OH_ and Δ*G*_*OHO_ for M@NC.

***Figure S16***. Free energy diagram for partially *O selective M′M@NC.

***Figure S17***. Calculated kinetic barriers for the O-O coupling via OCM pathway.

***Figure S18*.** Comparison of descriptors Δ*G*_*OH-*OH_ and Δ*G*_*O_.

***Figure S19***. PDOS, -COHP and Charge density differences of NiPd@NC.

***Figure S20***. Free energy diagrams of NiPd@NC, Ni_2_@NC and Pd_2_@NC.

***Figure S21***. The ∆G_*OH_ versus d band center and the ICOHP.

***Figure S22***. The Electronic structures of NiPt@NC, CuPd@NC and CuPt@NC.

***Figure S23***. The Relationship between bond length of M2 dimers and overpotential.

***Figure S24***. Free energy diagrams for NiPd@NC, CuPd@NC and CuPt@NC at different potentials at pH=1 and 13 respectively.

1. **Tables**

***Table S1***. The calculated *E*_cal_, *E*_ZPE_ and TS at T = 298 K for gaseous molecules.

***Table S2***. The bond length (Å) between the two transition-metal atoms (M_2_).

***Table S3***. The charge transfer from M_2_ dimer to N-doped graphene.

***Table S4***. Electronegativities of metals, which are from reference database.

**Table S5.** Computed energy of *E*_M,bulk_, *E*_M,gas_, *E*_coh-cal_ and Δ*E*_coh-exp_ were listed for validation of computational methods used.

***Table S6***. Computed BE, *E*_coh-ave_ and *E*_agg_ of FeM dimer embeded in N-doped graphene.

***Table S7***. Computed BE, *E*_coh-ave_ and *E*_agg_ of CoM dimer embeded in N-doped graphene.

***Table S8***. Computed BE, *E*_coh-ave_ and *E*_agg_ of NiM dimer embeded in N-doped graphene.

***Table S9***. Computed BE, *E*_coh-ave_ and *E*_agg_ of CuM dimer embeded in N-doped graphene.

***Table S10***. Computed dissolution potential (*U*_diss_) of FeM@NC.

***Table S11***. Computed dissolution potential (*U*_diss_) of CoM@NC.

***Table S12***. Computed dissolution potential (*U*_diss_) of NiM@NC.

***Table S13***. Computed dissolution potential (*U*_diss_) of CuM@NC.

***Table S14***. The optimized adsorption configuration for *OH/FeM@NC.

***Table S15***. The optimized adsorption configuration for *OH-*OH/FeM@NC.

***Table S16***. The optimized adsorption configuration for *OH-*OH/FeM@NC.

***Table S17***. The optimized adsorption configuration for *OH-*OH/FeM@NC.

***Table S18***. The optimized adsorption configuration for *OH/CoM@NC.

***Table S19***. The optimized adsorption configuration for *OH-*OH/CoM@NC.

***Table S20***. The optimized adsorption configuration for *OH-*OH/CoM@NC.

***Table S21***. The optimized adsorption configuration for *OH-*OH/CoM@NC.

***Table S22***. The optimized adsorption configuration for *OH/NiM@NC.

***Table S23***. The optimized adsorption configuration for *OH-*OH/NiM@NC.

***Table S24***. The optimized adsorption configuration for *OH-*OH/NiM@NC.

***Table S25***. The optimized adsorption configuration for *OH-*OH/NiM@NC.

***Table S26***. The optimized adsorption configuration for *OH/CuM@NC.

***Table S27***. The optimized adsorption configuration for *OH-*OH/CuM@NC.

***Table S28***. The optimized adsorption configuration for *OH-*OH/CuM@NC.

***Table S29***. The optimized adsorption configuration for *OH-*OH/CuM@NC.

***Table S30***. The computed *η* of the FeM, CoM, NiM, and CuM dimer on M′M@NC.

***Table S31***. Computed *E*_tot_, *E*_ZPE_ and TS of the intermediates on the CoCu@NC system.

***Table S32***. Computed *E*_tot_, *E*_ZPE_ and TS of the intermediates on the CoPd@NC system.

***Table S33***. Computed *E*_tot_, *E*_ZPE_ and TS of the intermediates on the NiCu@NC system.

***Table S34***. Computed *E*_tot_, *E*_ZPE_ and TS of the intermediates on the NiPd@NC system.

***Table S35***. Computed *E*_tot_, *E*_ZPE_ and TS of the intermediates on the NiPt@NC system.

***Table S36***. Computed *E*_tot_, *E*_ZPE_ and TS of the intermediates on the CuCu@NC system.

***Table S37***. Computed *E*_tot_, *E*_ZPE_ and TS of the intermediates on the CuPd@NC system.

***Table S38***. Computed *E*_tot_, *E*_ZPE_ and TS of the intermediates on the CuPt@NC system.

***Table S39***. Computed *E*_tot_, *E*_ZPE_ and TS of the intermediates on the Co@NC system.

***Table S40***. Computed *E*_tot_, *E*_ZPE_ and TS of the intermediates on the Cu@NC system.

***Table S41***. Computed *E*_tot_, *E*_ZPE_ and TS of the intermediates on the Ni@NC system.

***Table S42***. Computed *E*_tot_, *E*_ZPE_ and TS of the intermediates on the Pd@NC system.

***Table S43***. Computed *E*_tot_, *E*_ZPE_ and TS of the intermediates on the Pt@NC system.

***Table S44***. Computed *E*_tot_, *E*_ZPE_ and TS of the intermediates on the CoCu@NC system along the AEM.

***Table S45***. Computed *E*_tot_, *E*_ZPE_ and TS of the intermediates on the CoPd@NC system along the AEM.

***Table S46***. Computed *E*_tot_, *E*_ZPE_ and TS of the intermediates on the NiCu@NC system along the AEM.

***Table S47***. Computed *E*_tot_, *E*_ZPE_ and TS of the intermediates on the NiPd@NC system along the AEM.

***Table S48***. Computed *E*_tot_, *E*_ZPE_ and TS of the intermediates on the NiPt@NC system along the AEM.

***Table S49***. Computed *E*_tot_, *E*_ZPE_ and TS of the intermediates on the CuCu@NC system along the AEM.

***Table S50***. Computed *E*_tot_, *E*_ZPE_ and TS of the intermediates on the CuPd@NC system along the AEM.

***Table S51***. Computed *E*_tot_, *E*_ZPE_ and TS of the intermediates on the CuPt@NC system along the AEM.

***Table S52.*** Δ*G*_*OH→*OH-*OH_ of the second *OH adsorbed at different sites (M-M site, M-C site and M-M(anti) site) on the FeM@NC DACs.

***Table S53.*** Δ*G*_*OH→*OH-*OH_ of the second *OH adsorbed at different sites (M-M site, M-C site and M-M(anti) site) on the CoM@NC DACs.

***Table S54.*** Δ*G*_*OH→*OH-*OH_ of the second *OH adsorbed at different sites (M-M site, M-C site and M-M(anti) site) on the NiM@NC DACs.

***Table S55.*** Δ*G*_*OH→*OH-*OH_ of the second *OH adsorbed at different sites (M-M site, M-C site and M-M(anti) site) on the CuM@NC DACs.

***Table S56.*** Desorption free energy change (Δ*G*_*O2→O2(g)_) for *O_2_ toward O_2_ of FeM, CoM, NiM and CuM. Bolded font: With a benchmark of 1.17 eV for NiFe-CNG.

***Table S57.*** Adsorption free energy of FeM@Gr for *OH and the calculated relative energy of the optimized adsorption configuration for *OH-*OH. Bold fonts represent the most stable configuration.

***Table S58.*** Adsorption free energy of CoM@Gr for *OH and the calculated relative energy of the optimized adsorption configuration for *OH-*OH. Bold fonts represent the most stable configuration.

***Table S59.*** Adsorption free energy of NiM@Gr for *OH and the calculated relative energy of the optimized adsorption configuration for *OH-*OH. Bold fonts represent the most stable configuration.

***Table S60.*** Adsorption free energy of CuM@Gr for *OH and the calculated relative energy of the optimized adsorption configuration for *OH-*OH. Bold fonts represent the most stable configuration.

***Table S61***. Computed BE, *E*_agg_ and *U*_diss_.

***Table S62***. Computed *E*_tot_, *E*_ZPE_ and TS of the intermediates on the Ni_2_@NC system.

***Table S63***. Computed *E*_tot_, *E*_ZPE_ and TS of the intermediates on the Cu_2_@NC system.

***Table S64***. Computed *E*_tot_, *E*_ZPE_ and TS of the intermediates on the Rh_2_@NC system.

***Table S65***. Computed *E*_tot_, *E*_ZPE_ and TS of the intermediates on the Pd_2_@NC system.

***Table S66***. Computed *E*_tot_, *E*_ZPE_ and TS of the intermediates on the Ag_2_@NC system.

***Table S67***. Computed *E*_tot_, *E*_ZPE_ and TS of the intermediates on the Ir_2_@NC system.

***Table S68***. Computed *E*_tot_, *E*_ZPE_ and TS of the intermediates on the Pt_2_@NC system.

***Table S69***. Computed *E*_tot_, *E*_ZPE_ and TS of the intermediates on the Au_2_@NC system.

***Table S70.*** Gibbs free energy change (Δ*G*) for *OH toward *OH-*OH versus *OH → *O + H^+^ +e^−^ of FeM.

***Table S71.*** Gibbs free energy change (Δ*G*) for *OH toward *OH-*OH versus *OH → *O + H^+^ +e^−^ of CoM.

***Table S72.*** Gibbs free energy change (Δ*G*) for *OH toward *OH-*OH versus *OH → *O + H^+^ +e^−^ of NiM.

***Table S73.*** Gibbs free energy change (Δ*G*) for *OH toward *OH-*OH versus *OH → *O + H^+^ +e^−^ of CuM.

***Table S74.*** Gibbs free energy change (Δ*G*) for *OH toward *OH-*OH versus *OH → *O + H^+^ +e^−^ of M_2_.

***Table S75.*** Gibbs free energy change (Δ*G*) for *OH-*OH → *OH-*O + H^+^ +e^−^ versus *OH-*OH → *OOH + H^+^ +e^−^ of FeM.

***Table S76.*** Gibbs free energy change (Δ*G*) for *OH-*OH → *OH-*O + H^+^ +e^−^ versus *OH-*OH → *OOH + H^+^ +e^−^ of CoM.

***Table S77.*** Gibbs free energy change (Δ*G*) for *OH-*OH → *OH-*O + H^+^ +e^−^ versus *OH-*OH → *OOH + H^+^ +e^−^ of NiM.

***Table S78.*** Gibbs free energy change (Δ*G*) for *OH-*OH → *OH-*O + H^+^ +e^−^ versus *OH-*OH → *OOH + H^+^ +e^−^ of CuM.

***Table S79.*** Gibbs free energy change (Δ*G*) for *OH-*OH → *OH-*O + H^+^ +e^−^ versus *OH-*OH → *OOH + H^+^ +e^−^ of M_2_.

***Table S80.*** Energy difference between after and before oxygen coupling, Δ*E* = *E*_Post-coupling_ − *E*_Pre-coupling_.

***Table S81.*** Free energy changes for each intermediate of NiPd@NC under different conditions.

***Table S82.*** Free energy changes for each intermediate of CuPd@NC under different conditions.

***Table S83.*** Free energy changes for each intermediate of CuPt@NC under different conditions.


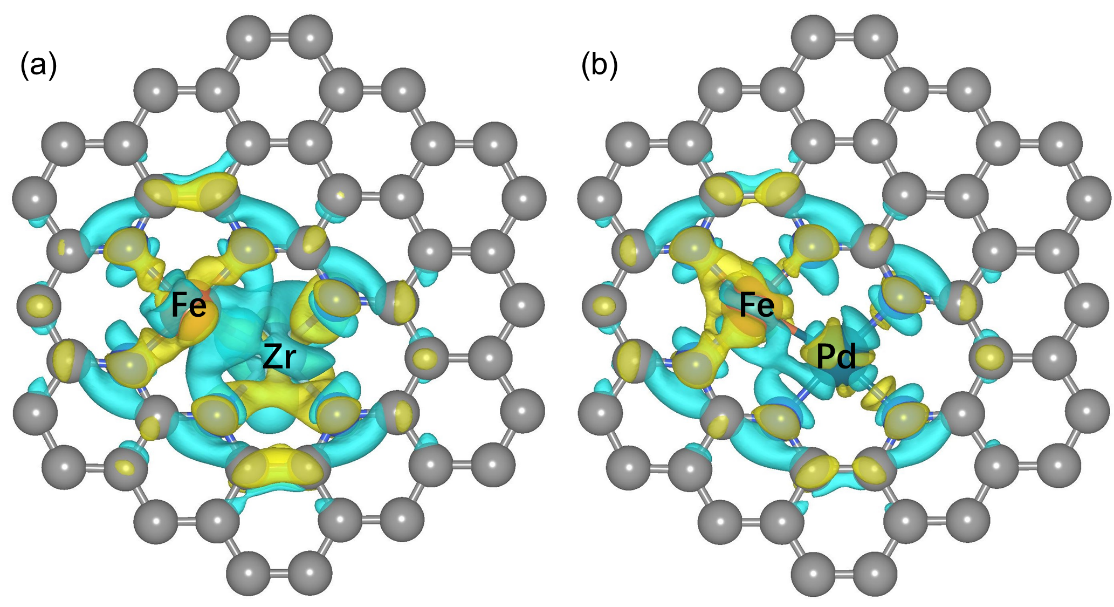


***Figure S1***. **Electronic structures**. Charge density differences of (a) FeZr@NC and (b) FePd@NC. The isosurface value is 0.004 e/Bohr^3^. The grey and blue spheres in the atomic models represent C and N atoms, respectively.


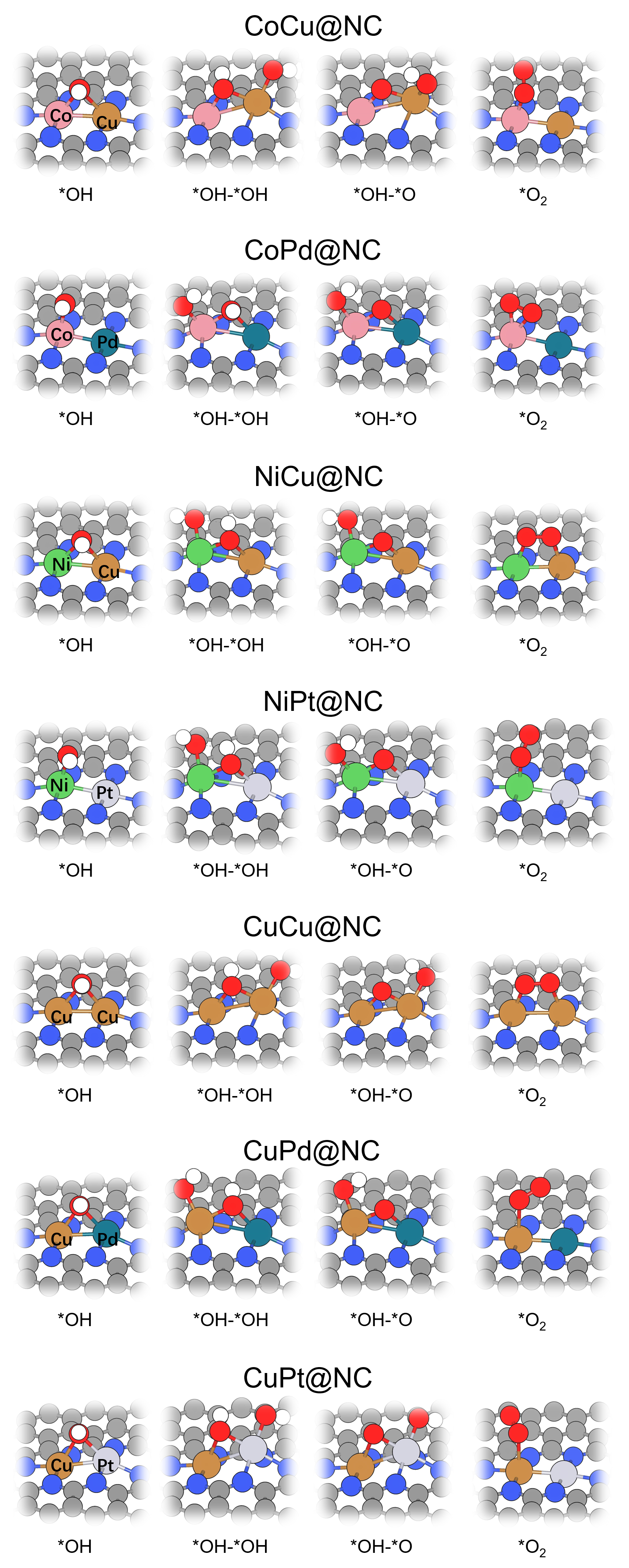


***Figure S2***. **Optimized configuration**. Optimized structures of oxygenated intermediates on M′M@NC surface. The white, red, grey and blue spheres in the atomic models represent H, O, C and N atoms, respectively.


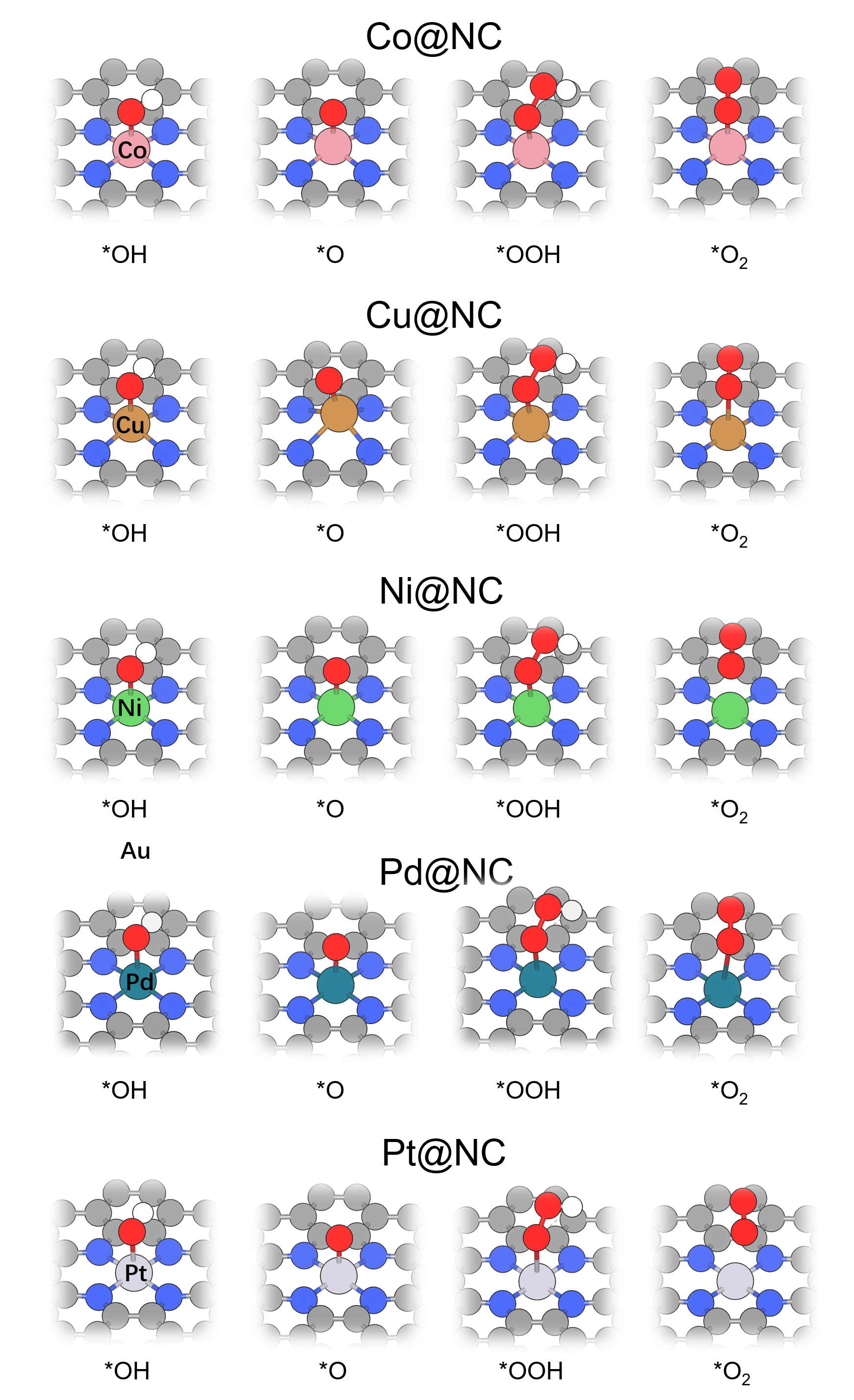


***Figure S3***. **Optimized configuration**. Optimized structures of oxygenated intermediates on M@NC surface. The white, red, grey and blue spheres in the atomic models represent H, O, C and N atoms, respectively.


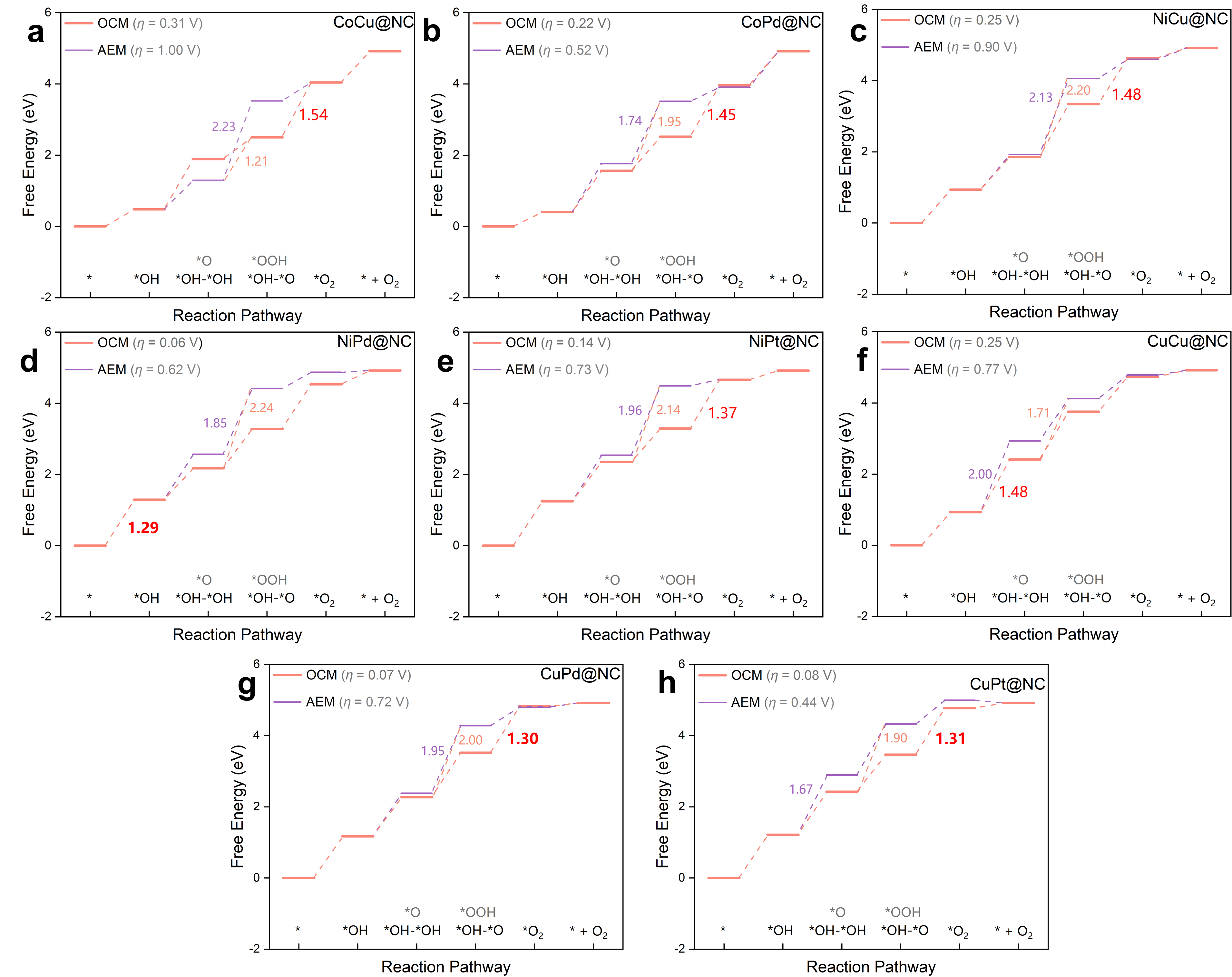


***Figure S4***. **Gibbs free energy diagrams.** Gibbs free energy diagrams of (a) CoCu@NC, (b) CoPd@NC, (c) NiCu@NC, (d) NiPd@NC, (e) NiPt@NC, (f) CuCu@NC, (g) CuPd@NC and (h) CuPt@NC for comparison as well as the crossing of potential reaction pathway.


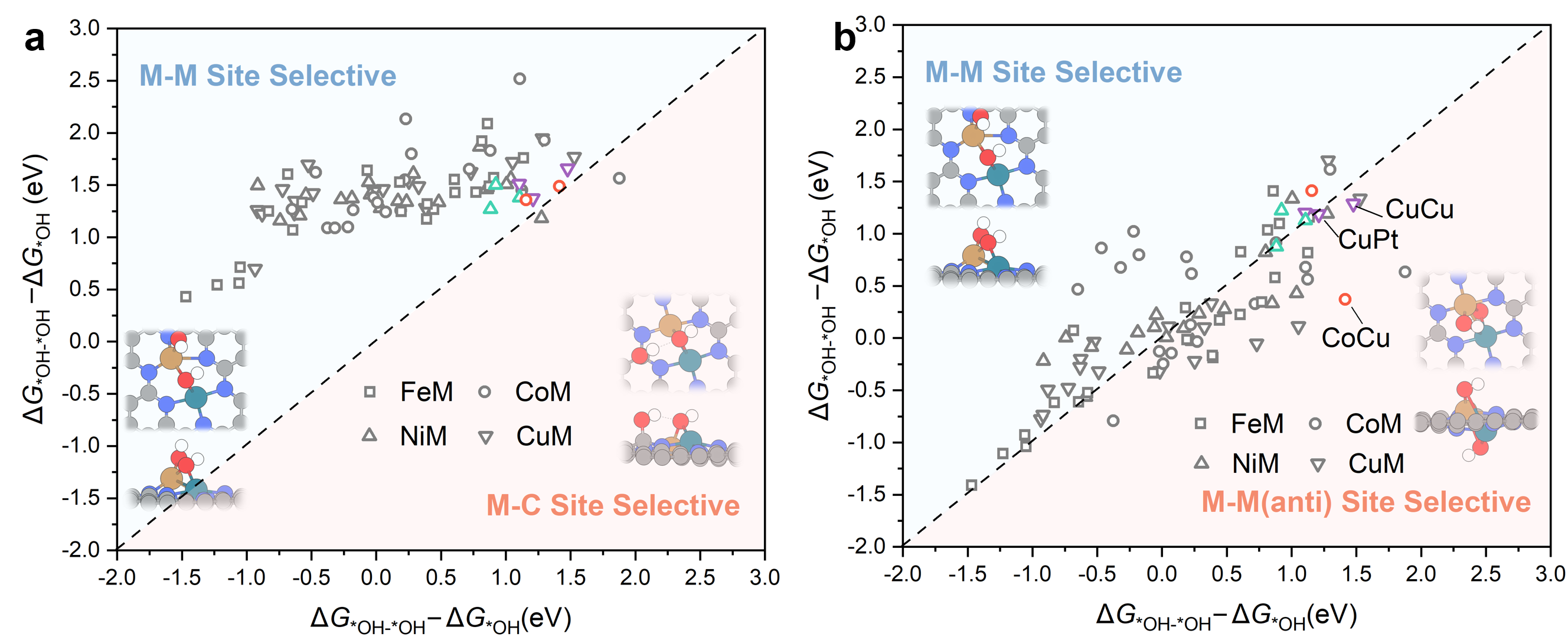


***Figure S5.*** **Selectivity of the** **oxygenated intermediates.** (a) Selectivity of the second *OH adsorption at the M-M site versus the M-C site. (b) Selectivity of the second *OH adsorption at the M-M site versus the M-M(anti) path. Insets show the schematic configuration of *OH-*OH/M′M@NC for different adsorption sites. The white, red, grey and blue spheres in the atomic models represent H, O, C and N atoms, respectively.


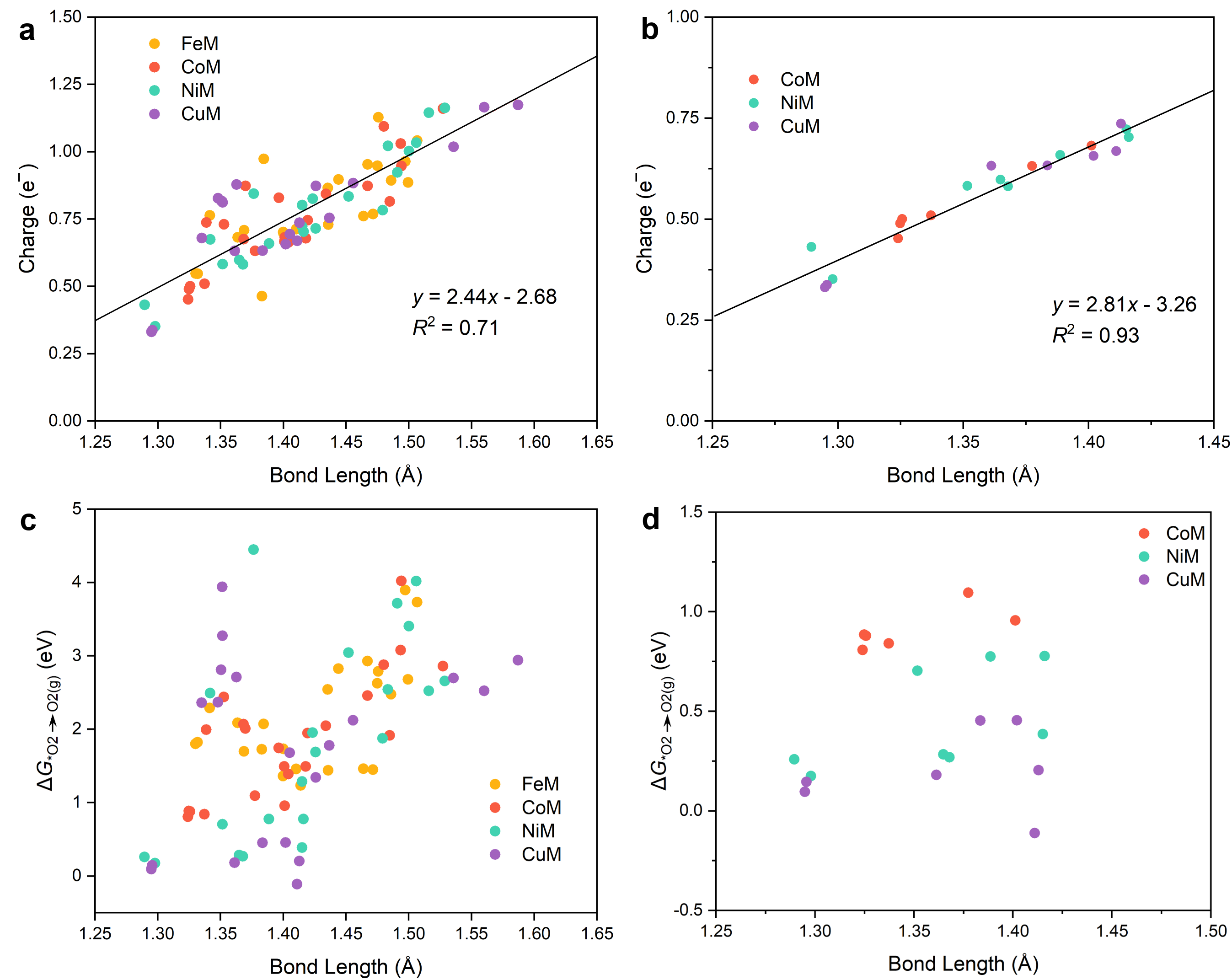


***Figure S6.*** **Correlation of *O_2_ bond length with related parameters.** (a, b) The relationship between *O_2_ bond length and the charge it obtained. (c, d) Relationship between *O_2_ bond length and Δ*G*_*O2→O2(g)_.


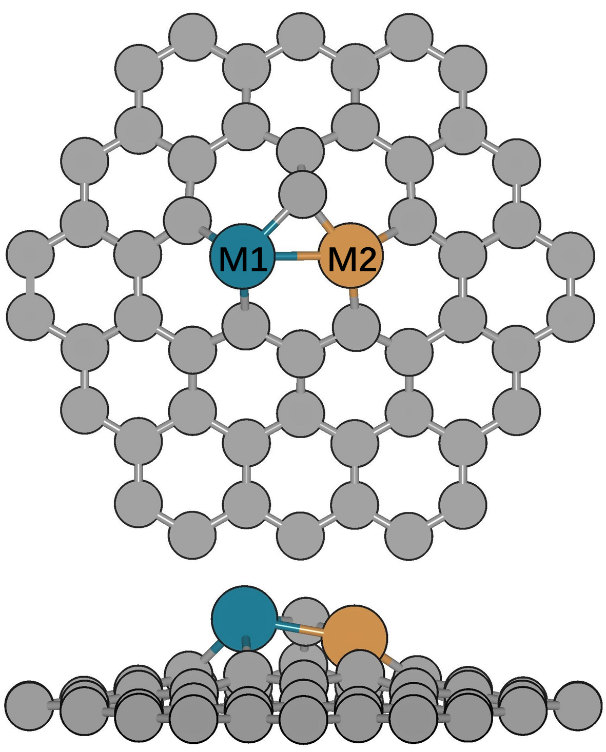


***Figure S7.*** **Optimized configuration**. Optimized structure of M′M@Gr monolayer in a *p*(6 × 6) supercell.


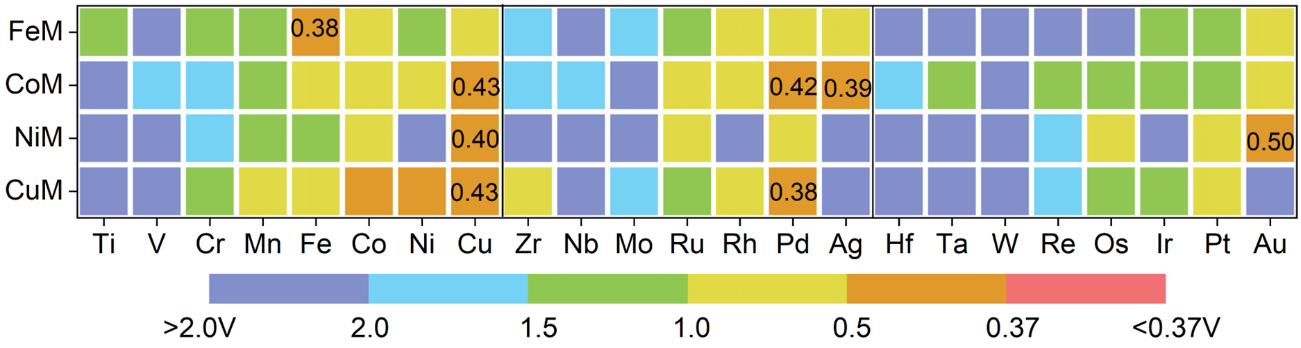


***Figure S8.*** **The activity map.** The computed overpotential (*η*) of the FeM, CoM, NiM, and CuM dimers on M′M@Gr.


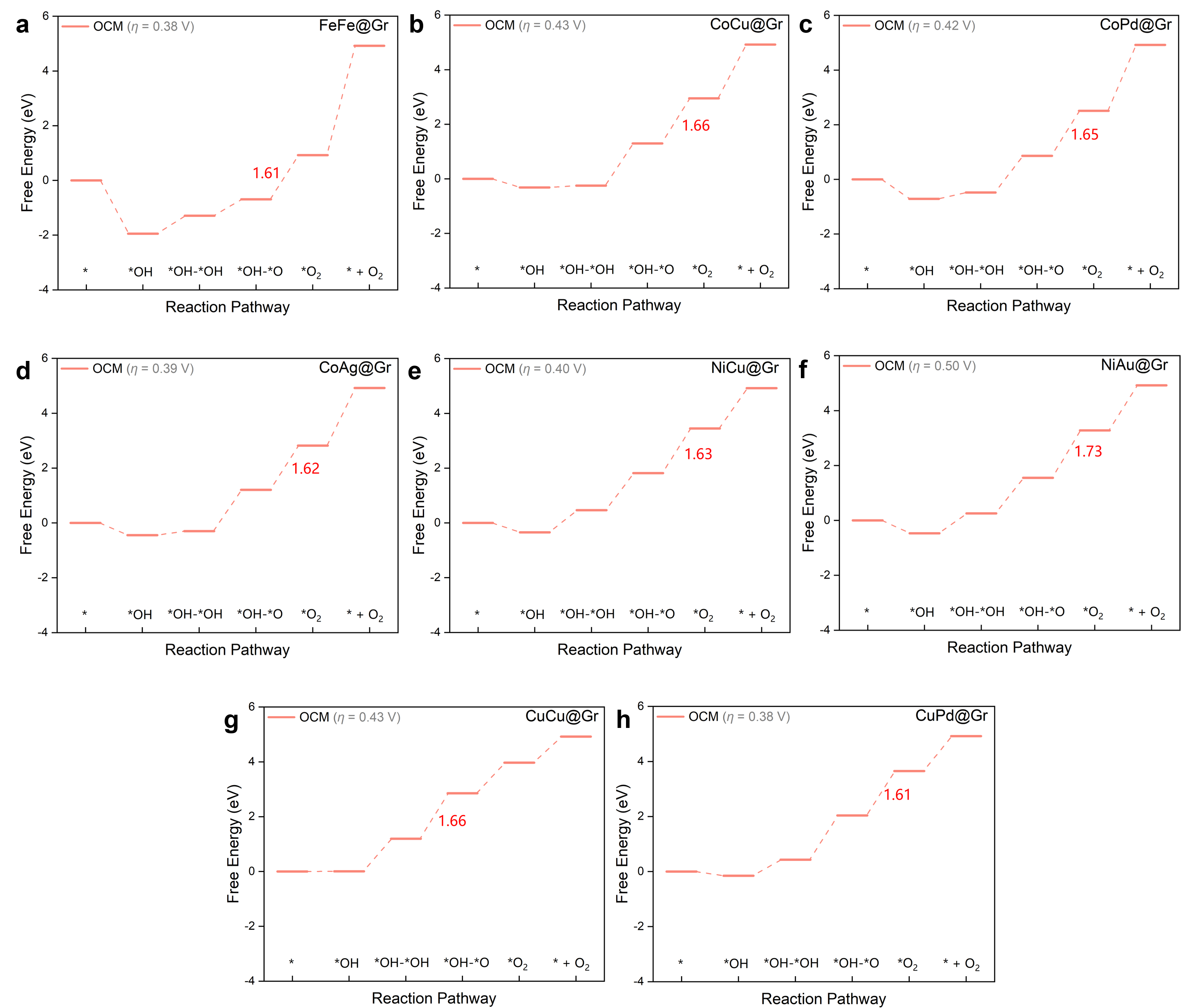


***Figure S9.*** **Gibbs free energy diagrams.** Gibbs free energy diagram of (a) FeFe@Gr, (b) CoCu@Gr, (c) CoPd@Gr, (d) CoAg@Gr, (e) NiCu@Gr, (f) NiAu@Gr, (g) CuCu@Gr and (h) CuPd@Gr along the OCM of M′M@Gr.


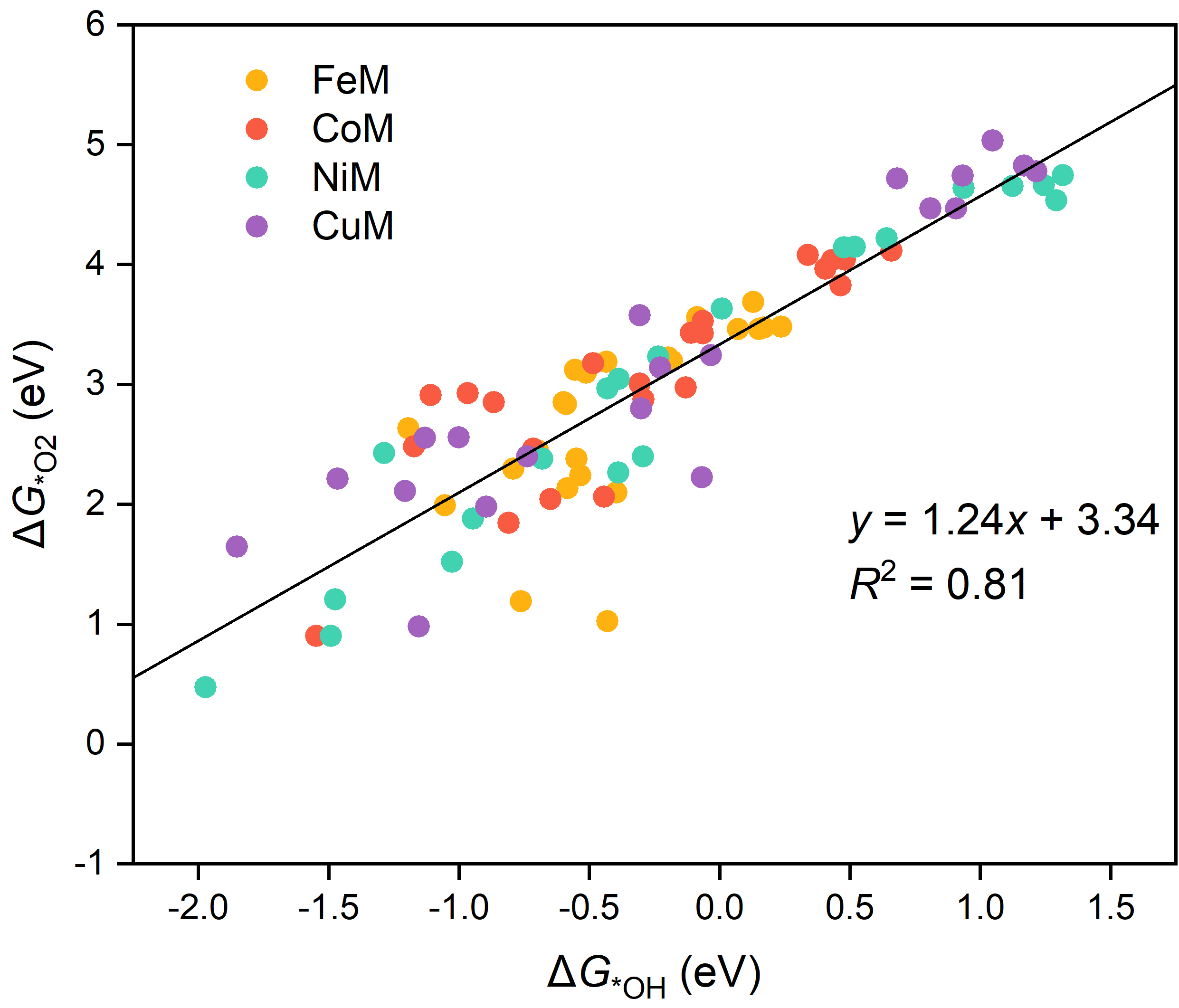


***Figure S10***. **Scaling relationship.** Scaling relations for *O_2_/*OH in terms of adsorption free energy (Δ*G*).


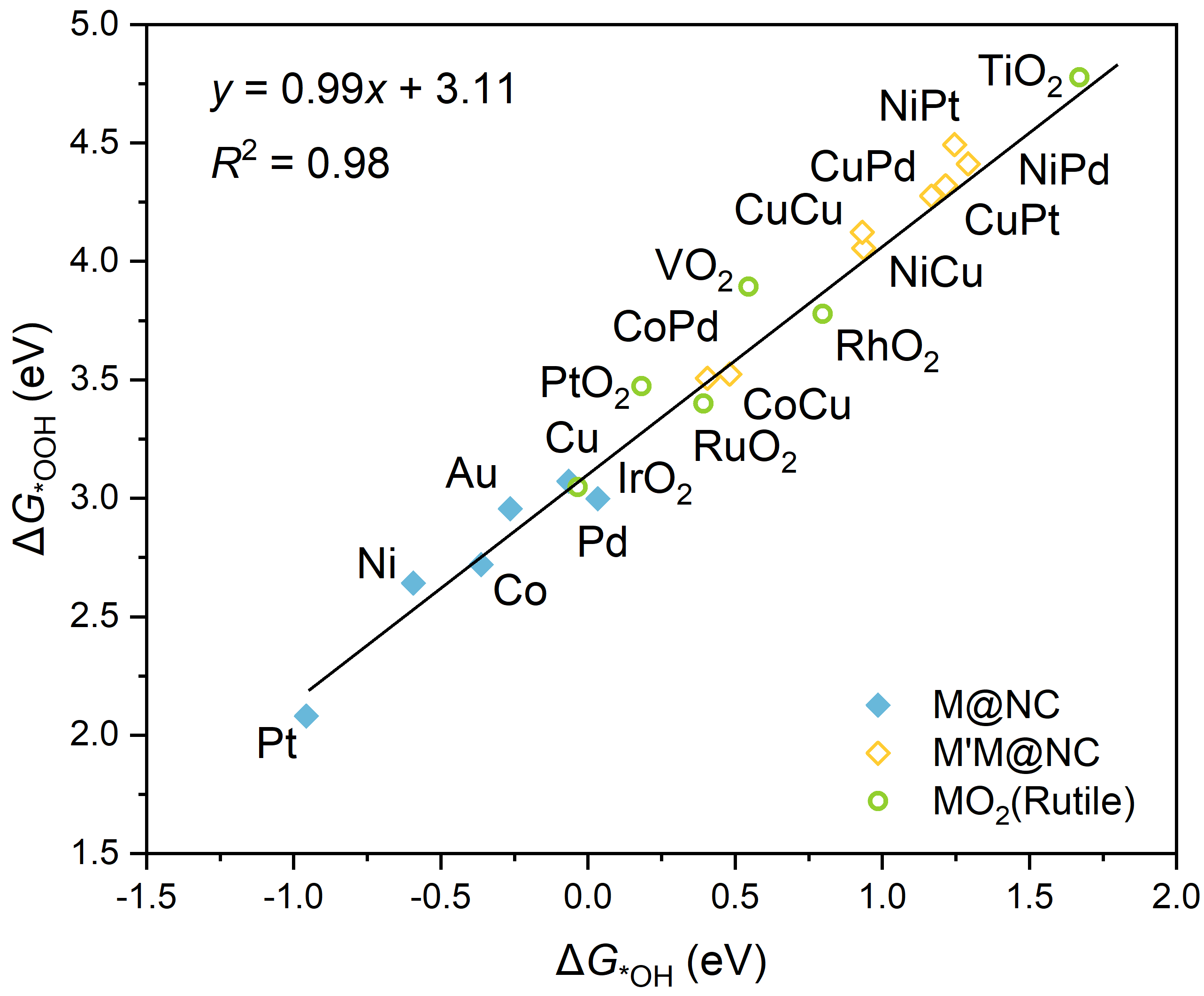


***Figure S11.*** **Scaling relationship.** Relationship between ΔG_*OH_ and ΔG_*OOH_ for M@NC, M′M@NC and rutile MO_2_ (M = Ti, V, Ru, Rh, Ir and Pt).


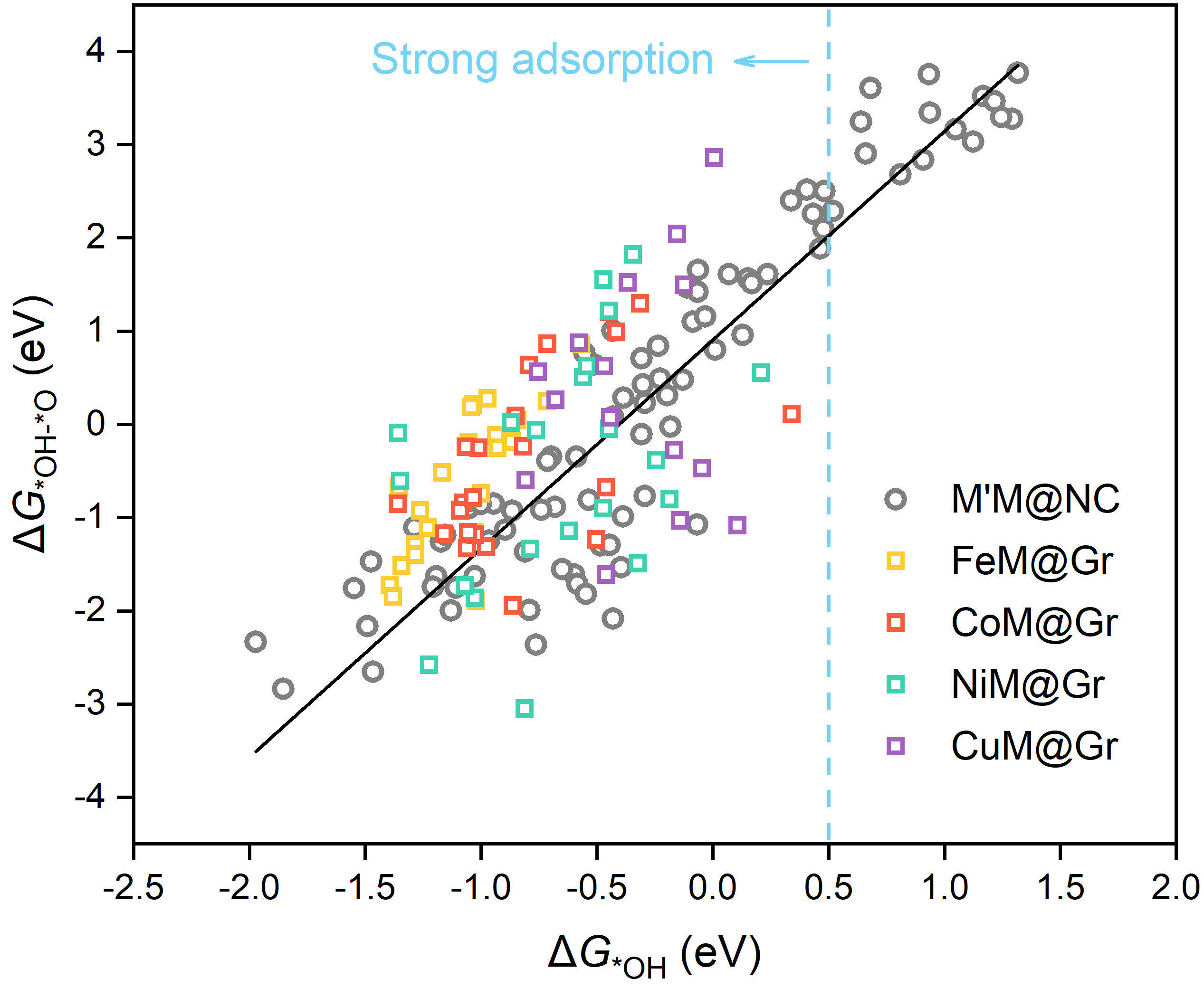


***Figure S12.*** **Scaling relationship.** Relationship between Δ*G*_*OH_ and Δ*G*_*OH-*O_ for M′M@Gr.


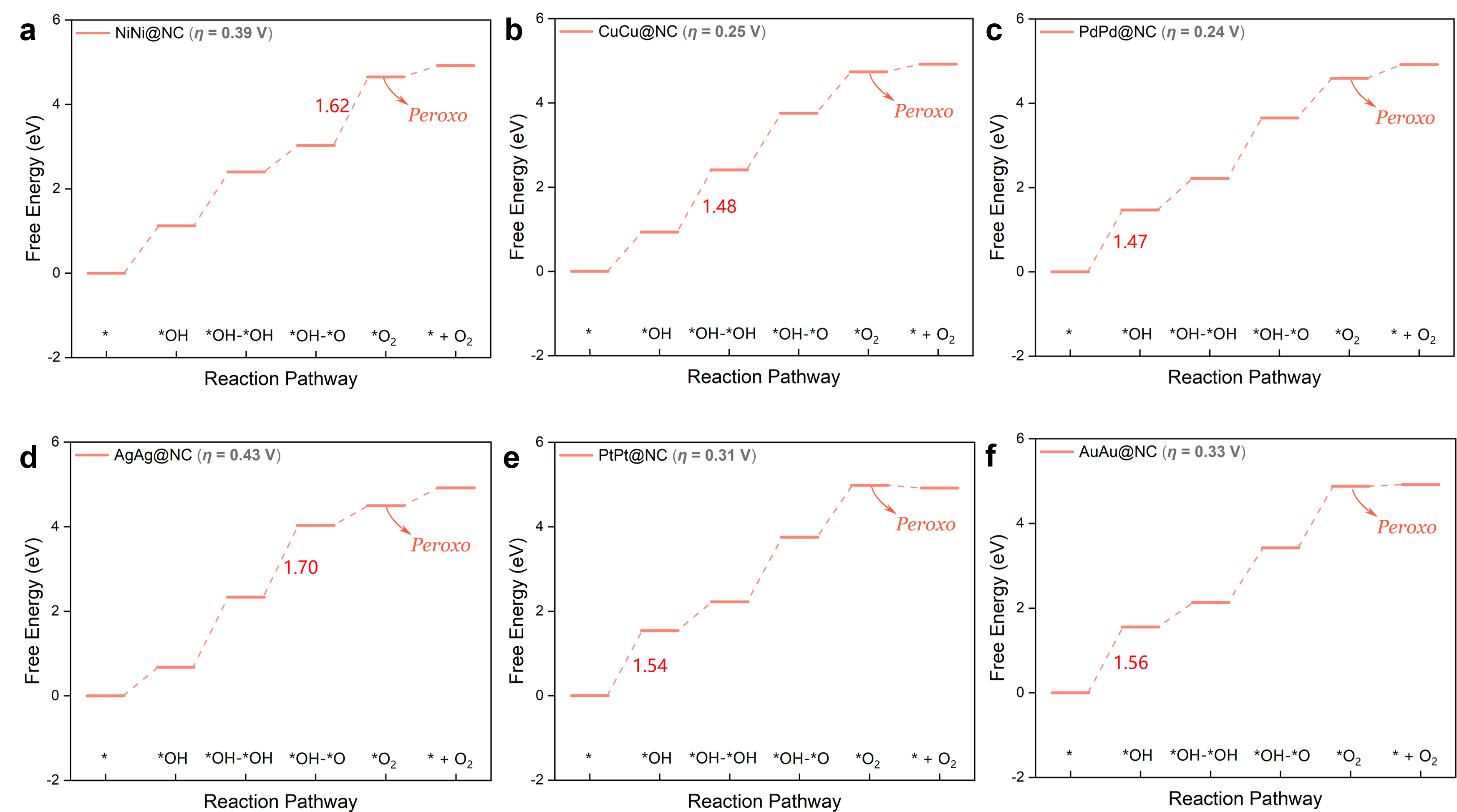


***Figure S13***. **Gibbs free energy diagrams.** Gibbs free energy diagrams of (a) NiNi@NC, (b) CuCu@NC, (c) PdPd@NC, (d) AgAg@NC, (e) PtPt@NC and (f) AuAu@NC for OER.


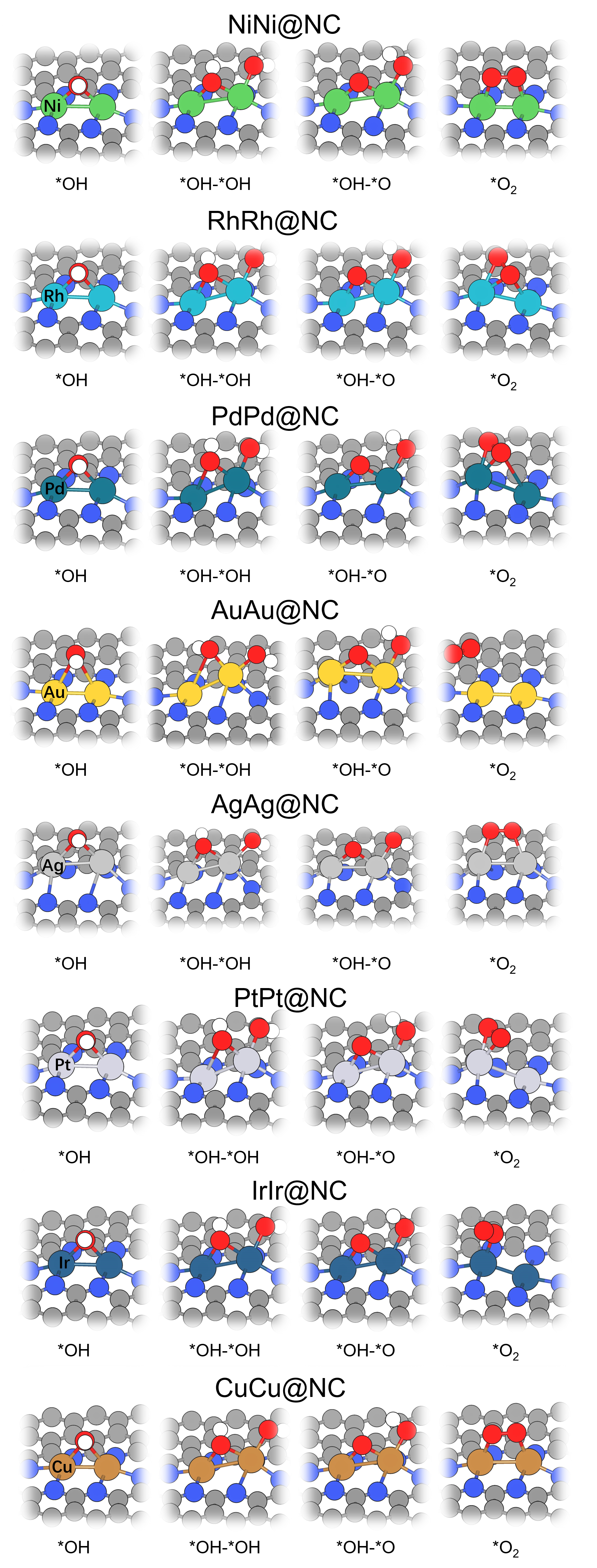


***Figure S14***. **Optimized configuration**. Optimized structures of oxygenated intermediates on M_2_@NC surface. The white, red, grey and blue spheres in the atomic models represent H, O, C and N atoms, respectively.


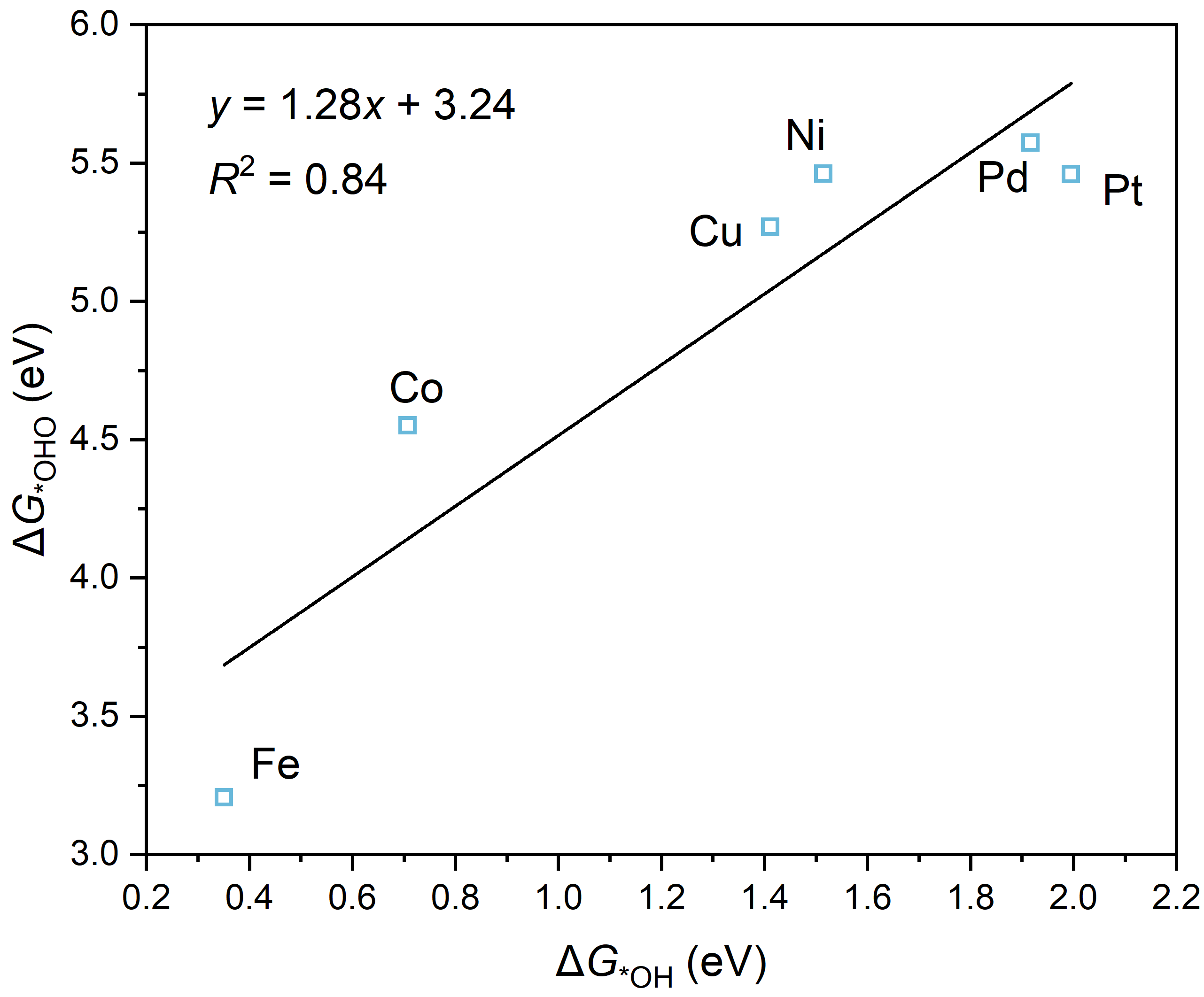


***Figure S15***. **Scaling relationship.** Relationship between Δ*G*_*OH_ and Δ*G*_*OHO_ for M@NC.


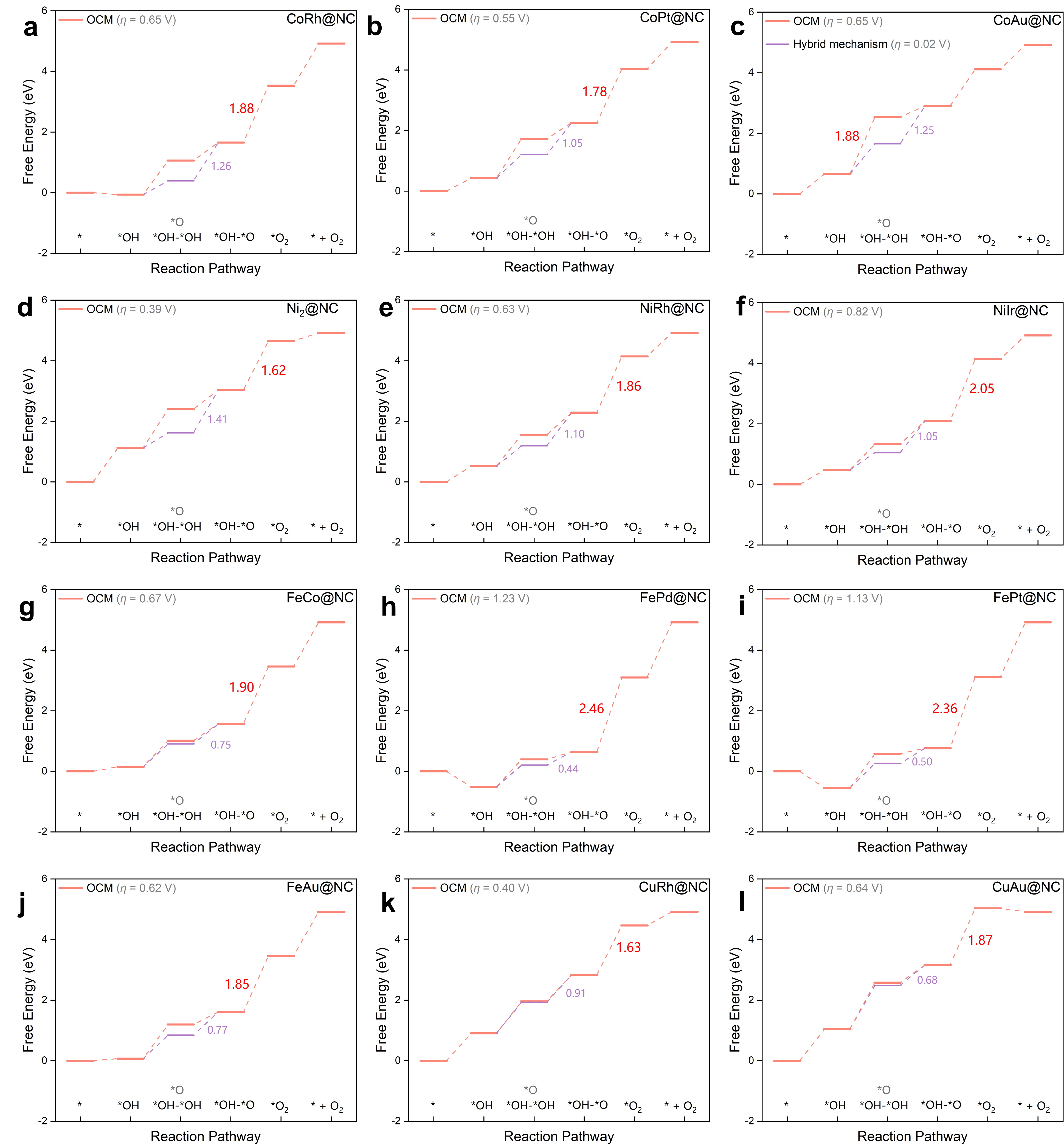


***Figure S16***. **Gibbs free energy diagrams.** Gibbs free energy diagrams of (a) CoRh@NC, (b) CoPt@NC, (c) CoAu@NC, (d) NiNi@NC, (e) NiRh@NC, (f) NiIr@NC, (g) FeCo@NC, (h) FePd@NC, (i) FePt@NC, (j) FeAu@NC, (k) CuRh@NC and (l) CuAu@NC for partially *O selective M′M@NC.


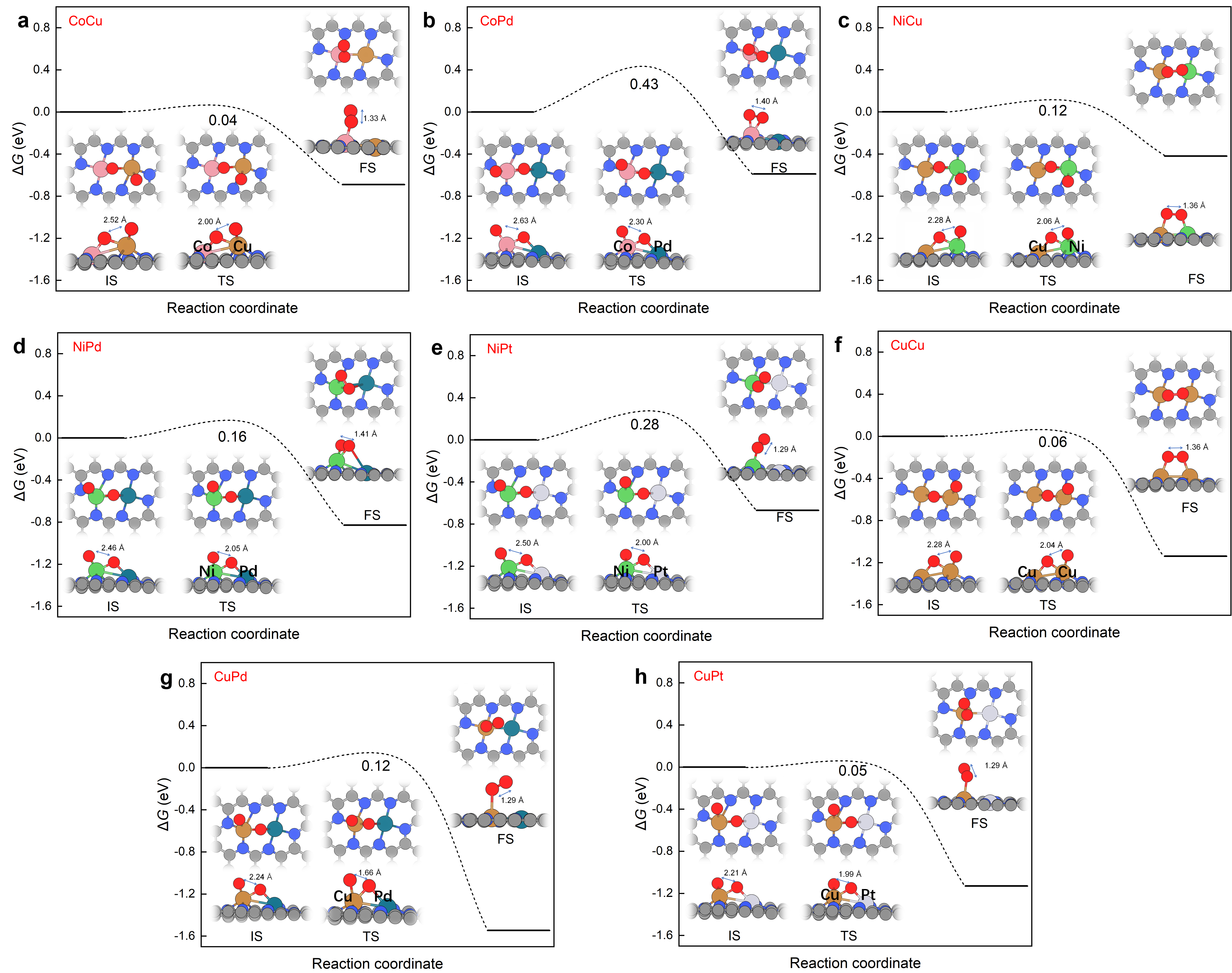


***Figure S17***. **Calculated kinetic barriers.** Calculated kinetic barriers for the O-O coupling via OCM pathway on (a) CoCu@NC, (b) CoPd@NC, (c) NiCu@NC, (d) NiPd@NC, (e) NiPt@NC, (f) CuCu@NC, (g) CuPd@NC and (h) CuPt@NC. IS, TS, and FS denote the initial state, transition state and final state, respectively. The white, red, grey and blue spheres in the atomic models represent H, O, C and N atoms, respectively.


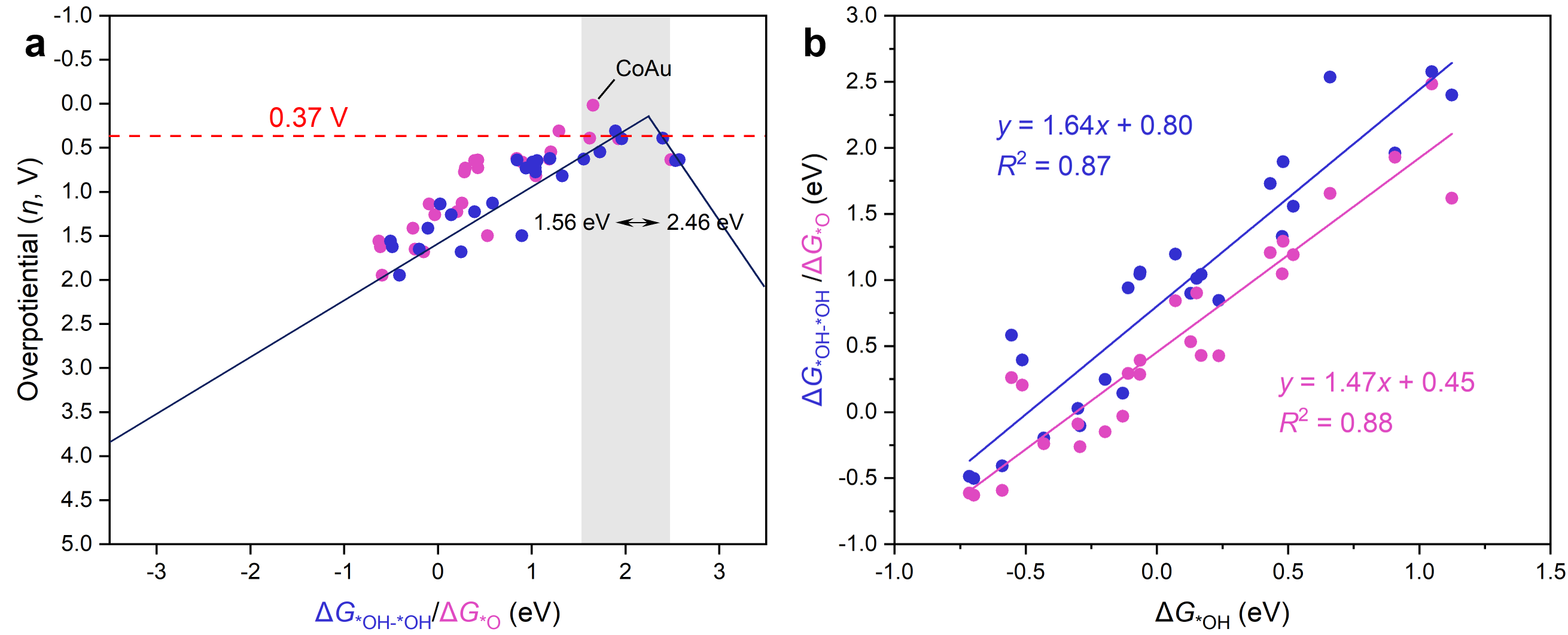


***Figure S18*. Applicability of descriptors to *O-selective DACs.** (a) Volcano plot of DACs with *O-selectivity (or following the hybrid mechanism). Green represents Δ*G*_*OH-*OH_ as the descriptor and red represents Δ*G*_*O_ as the descriptor. (b) Scaling relationships of Δ*G*_*OH_/Δ*G*_*OH-*OH_ (blue) and Δ*G*_*OH_/Δ*G*_*O_ (purple) for DACs with *O selectivity (or following the hybrid mechanism), respectively.


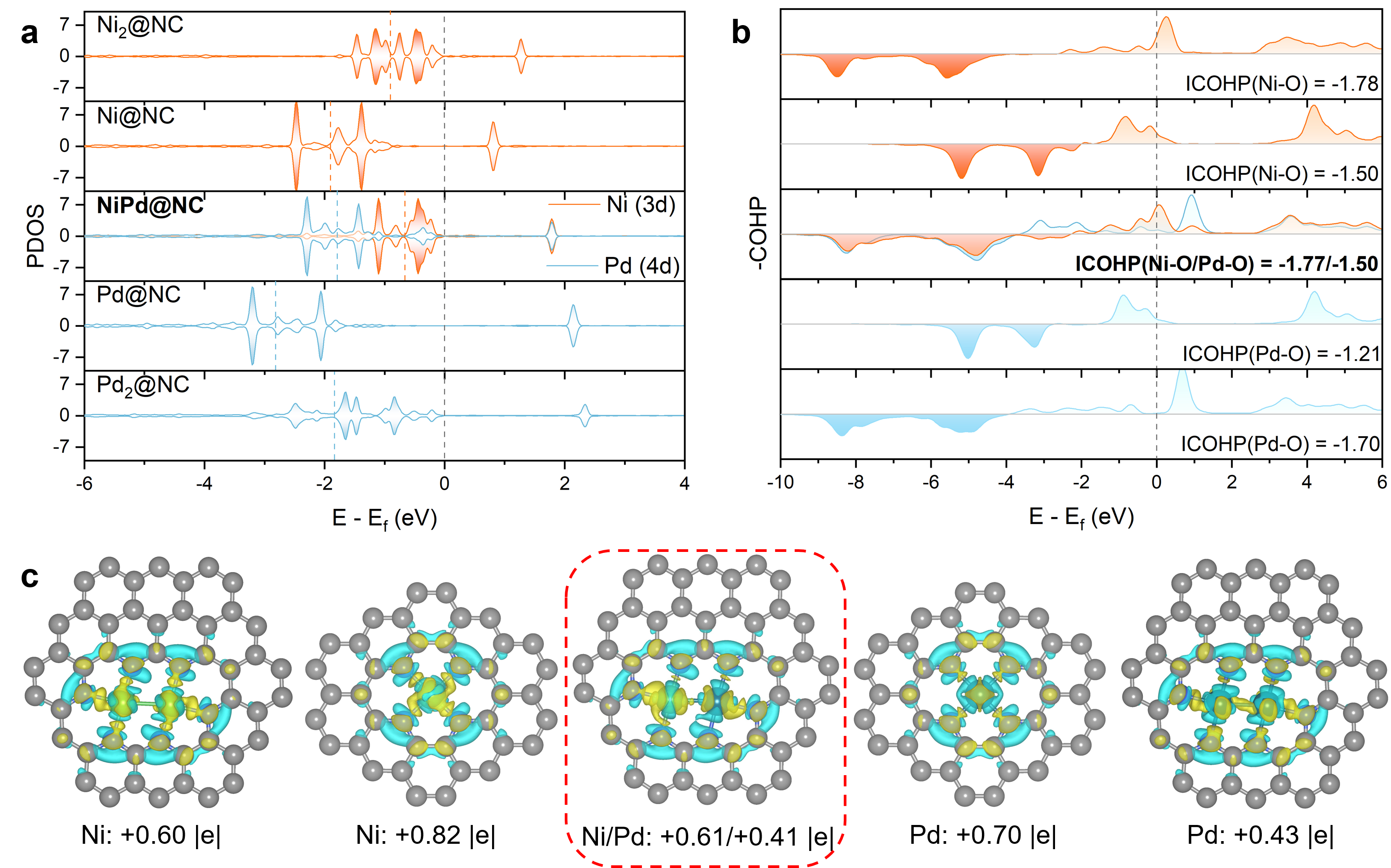


***Figure S19*.** **Electronic structures**. (a) The partial density of states (PDOS) for the Ni/Pd-d orbitals of Ni_2_@NC, Ni@NC, NiPd@NC, Pd@NC and Pd_2_@NC, respectively. (b) Crystal orbital Hamilton population (-COHP) for Ni_2_@NC, Ni@NC, NiPd@NC, Pd@NC and Pd_2_@NC, respectively. (c) Charge density differences (Δ*ρ =ρ*_(slab+ads)_ −*ρ*_(slab)_ −*ρ*_(ads)_) of Ni_2_@NC, Ni@NC, NiPd@NC, Pd@NC and Pd_2_@NC, respectively, and isosurface level = 0.004 e/Bohr^3^. Yellow: charge accumulation; cyan: charge depletion.


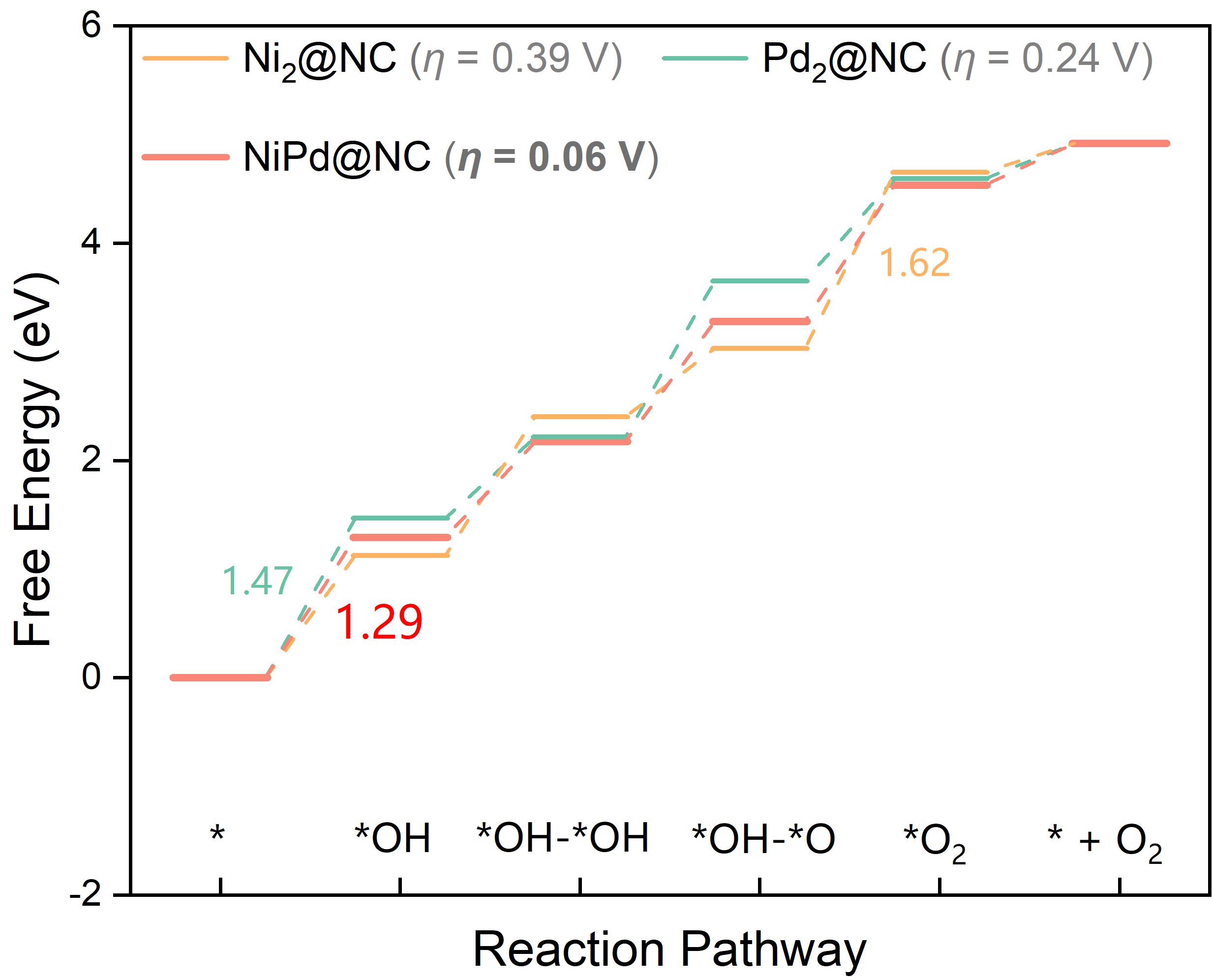


***Figure S20***. **Free energy diagrams.** Free energy diagrams of NiPd@NC, Ni_2_@NC and Pd_2_@NC.


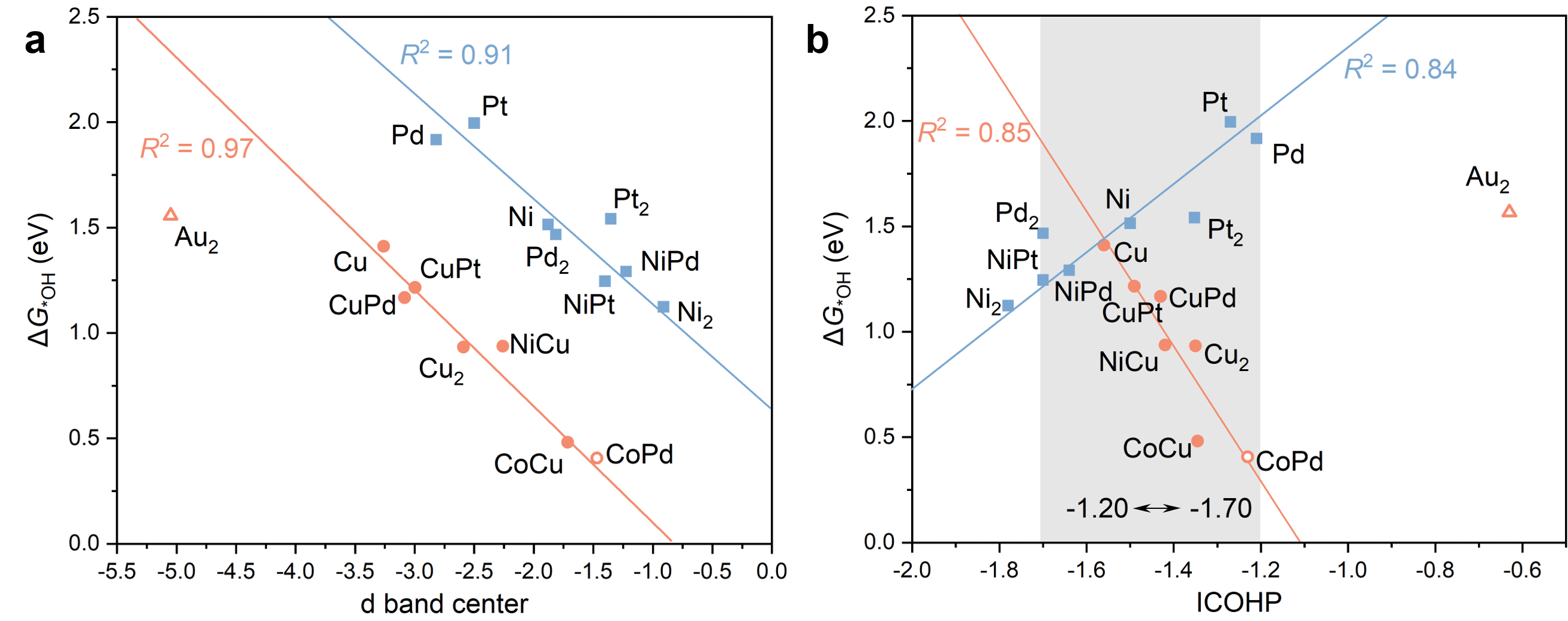


***Figure S21***. **Electronic structures**. (a) Relationship between the d band center and the Δ*G*_*OH_. (b) Relationship between the ICOHP and the Δ*G*_*OH_. Open symbols represent M_2_ dimers that deviate from the linear correlation, where CoPd does not belong to the CuM dimers.


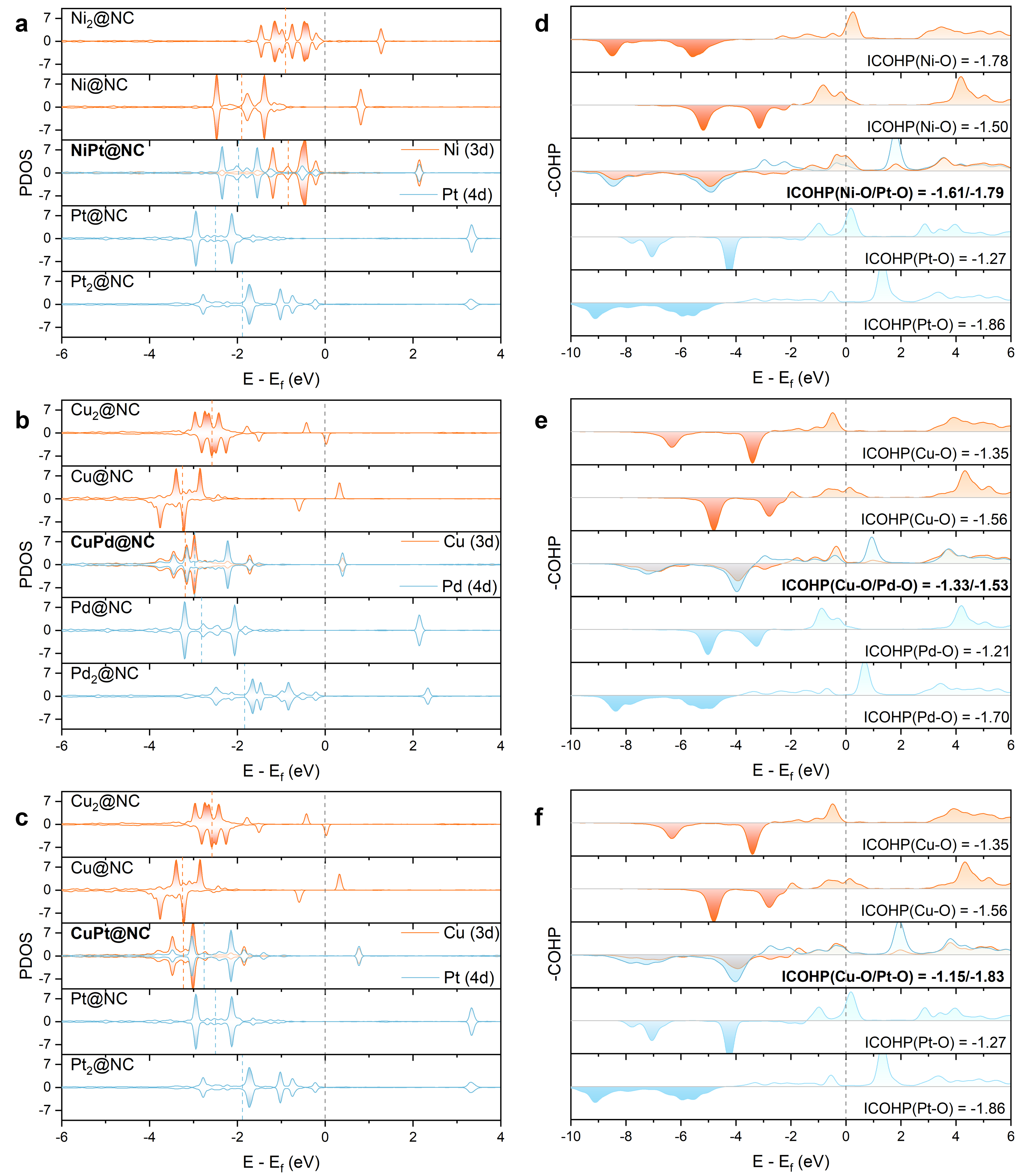


***Figure S22***. **Electronic structures**. (a-c) Partial density of states (PDOS) of NiPt@NC, CuPd@NC, CuPt@NC and the corresponding homonuclear DACs as well as SACs, respectively. (d-f) Crystal orbital Hamiltonian groups (-COHP) of NiPt@NC, CuPd@NC, CuPt@NC and the corresponding homonuclear DACs and SACs, respectively.


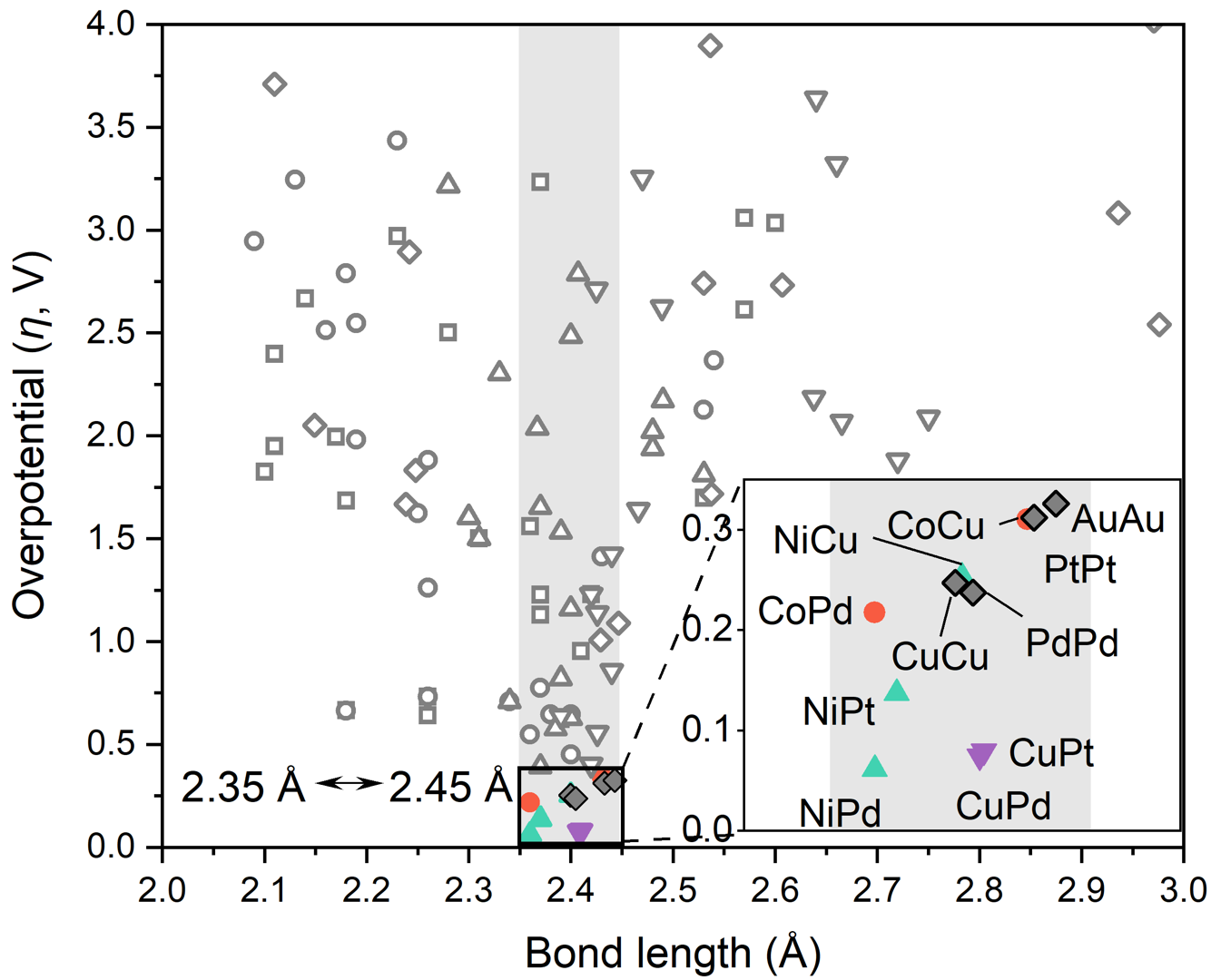


***Figure S23***. **The relationship between *η* and the bond length**. Variation in overpotential with a bond length of M_2_ dimers and the close-up view of the 11 high-performance DACs.


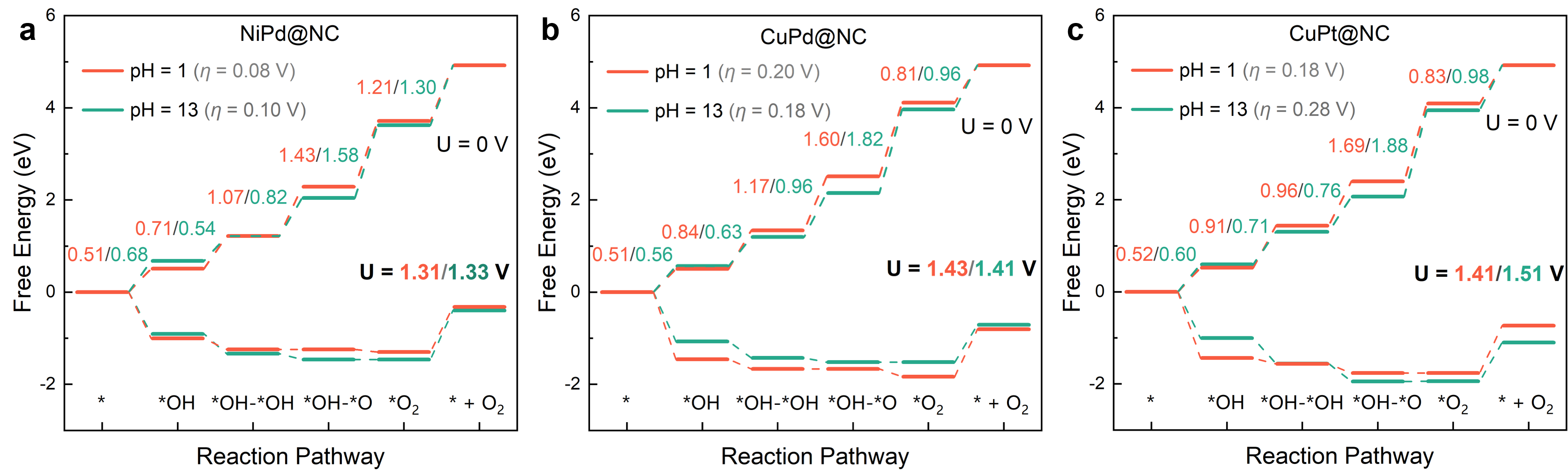


***Figure S24***. **Free energy diagrams**. Free energy diagrams for (a) NiPd@NC, (b) CuPd@NC and (c) CuPt@NC at different potentials at pH=1 and 13 respectively.

***Table S1***. The calculated total energies (*E*_cal_), zero-point energies (*E*_ZPE_) and entropy corrections (TS) at T = 298 K for gaseous molecules.

| Species | *E*_cal_ (eV) | *E*_ZPE_ (eV) | TS (eV) |
| --- | --- | --- | --- |
| H_2_ (g) | -6.70 | 0.29 | 0.40 |
| H_2_O (l) | -14.22 | 0.57 | 0.67 |

***Table S2***. The bond length (Å) between the two transition-metal atoms (M_2_).

|  | FeM | CoM | NiM | CuM |
| --- | --- | --- | --- | --- |
| Ti | 2.37 | 2.13 | 2.37 | 2.67 |
| V | 2.60 | 2.09 | 2.53 | 2.64 |
| Cr | 2.53 | 2.25 | 2.37 | 2.43 |
| Mn | 2.36 | 2.43 | 2.30 | 2.47 |
| Fe | 2.11 | 2.18 | 2.31 | 2.42 |
| Co | 2.18 | 2.26 | 2.34 | 2.43 |
| Ni | 2.31 | 2.34 | 2.37 | 2.40 |
| Cu | 2.42 | 2.43 | 2.40 | 2.40 |
| Zr | 2.57 | 2.53 | 2.48 | 2.75 |
| Nb | 2.23 | 2.19 | 2.49 | 2.66 |
| Mo | 2.11 | 2.16 | 2.40 | 2.49 |
| Ru | 2.18 | 2.26 | 2.40 | 2.44 |
| Rh | 2.26 | 2.40 | 2.40 | 2.42 |
| Pd | 2.37 | 2.36 | 2.36 | 2.41 |
| Ag | 2.41 | 2.40 | 2.39 | 2.56 |
| Hf | 2.57 | 2.54 | 2.48 | 2.72 |
| Ta | 2.28 | 2.23 | 2.41 | 2.64 |
| W | 2.14 | 2.18 | 2.28 | 2.47 |
| Re | 2.10 | 2.19 | 2.33 | 2.43 |
| Os | 2.17 | 2.26 | 2.39 | 2.44 |
| Ir | 2.26 | 2.37 | 2.39 | 2.43 |
| Pt | 2.37 | 2.36 | 2.37 | 2.41 |
| Au | 2.39 | 2.38 | 2.38 | 2.39 |

***Table S3***. The charge transfer from M_2_ dimer to N-doped graphene.

|  | FeM | CoM | NiM | CuM |
| --- | --- | --- | --- | --- |
| Ti | 2.08 | 1.83 | 1.88 | 1.98 |
| V | 1.74 | 1.76 | 1.76 | 1.85 |
| Cr | 1.89 | 1.72 | 1.64 | 1.76 |
| Mn | 1.85 | 1.65 | 1.63 | 1.77 |
| Fe | 1.50 | 1.48 | 1.47 | 1.53 |
| Co | 1.48 | 1.35 | 1.31 | 1.32 |
| Ni | 1.47 | 1.31 | 1.18 | 1.32 |
| Cu | 1.53 | 1.32 | 1.32 | 1.36 |
| Zr | 2.21 | 2.12 | 2.06 | 2.15 |
| Nb | 1.93 | 1.89 | 1.83 | 1.97 |
| Mo | 1.85 | 1.75 | 1.73 | 1.81 |
| Ru | 1.42 | 1.36 | 1.29 | 1.33 |
| Rh | 1.38 | 1.19 | 1.14 | 1.15 |
| Pd | 1.23 | 1.11 | 1.02 | 1.23 |
| Ag | 1.43 | 1.24 | 1.26 | 1.18 |
| Hf | 2.11 | 2.01 | 2.03 | 2.12 |
| Ta | 1.97 | 1.92 | 1.95 | 2.00 |
| W | 1.93 | 1.84 | 1.88 | 1.92 |
| Re | 1.62 | 1.62 | 1.59 | 1.75 |
| Os | 1.47 | 1.53 | 1.29 | 1.31 |
| Ir | 1.36 | 1.17 | 1.12 | 1.17 |
| Pt | 1.20 | 1.11 | 1.02 | 1.21 |
| Au | 1.38 | 1.15 | 1.19 | 1.25 |

***Table S4***. Electronegativities of metals, which are from reference database.

| M | electronegativity |
| --- | --- |
| Ti | 1.54 |
| V | 1.63 |
| Cr | 1.66 |
| Mn | 1.55 |
| Fe | 1.83 |
| Co | 1.88 |
| Ni | 1.91 |
| Cu | 1.90 |
| Zr | 1.33 |
| Nb | 1.60 |
| Mo | 2.16 |
| Ru | 2.20 |
| Rh | 2.28 |
| Pd | 2.20 |
| Ag | 1.93 |
| Hf | 1.30 |
| Ta | 1.50 |
| W | 2.36 |
| Re | 1.90 |
| Os | 2.20 |
| Ir | 2.20 |
| Pt | 2.28 |
| Au | 2.54 |

**Table S5.** Computed energy of single metal atom in the bulk phase (*E*_M,bulk_), gas phase (*E*_M,gas_), cohesive energy (*E*_coh-cal_)^a^ and Experimental values (Δ*E*_coh-exp_) were listed for validation of computational methods used.

| Metal | *E*_M,bulk_ | *E*_M,gas_ | Δ*E*_coh-cal_ | Δ*E*_coh-exp_ |
| --- | --- | --- | --- | --- |
| Ti | -7.83 | -2.53 | -5.30 | -4.88 |
| V | -8.99 | -3.64 | -5.36 | -5.34 |
| Cr | -9.51 | -5.44 | -4.07 | -4.11 |
| Mn | -8.99 | -5.15 | -3.84 | -2.91 |
| Fe | -8.24 | -3.38 | -4.86 | -4.30 |
| Co | -6.82 | -1.90 | -4.92 | -4.41 |
| Ni | -5.41 | -0.60 | -4.81 | -4.47 |
| Cu | -3.73 | -0.22 | -3.51 | -3.50 |
| Zr | -8.52 | -2.36 | -6.16 | -6.27 |
| Nb | -10.21 | -3.22 | -7.00 | -7.60 |
| Mo | -10.93 | -4.65 | -6.28 | -6.83 |
| Ru | -9.24 | -2.48 | -6.76 | -6.76 |
| Rh | -7.25 | -1.27 | -5.98 | -5.76 |
| Pd | -5.22 | -1.47 | -3.75 | -3.50 |
| Ag | -2.72 | -0.20 | -2.51 | -2.95 |
| Hf | -9.92 | -3.51 | -6.41 | -6.44 |
| Ta | -11.81 | -3.70 | -8.11 | -8.10 |
| W | -12.95 | -4.56 | -8.39 | -8.91 |
| Re | -12.43 | -4.61 | -7.82 | -8.04 |
| Os | -11.25 | -2.92 | -8.33 | -8.18 |
| Ir | -8.85 | -1.56 | -7.29 | -6.95 |
| Pt | -6.10 | -0.54 | -5.56 | -5.85 |
| Au | -3.22 | -0.18 | -3.03 | -3.81 |

^a^ *E*_coh-cal_ = (*E*_M,bulk_ - n*E*_M,gas_)/n, where *E*_M,gas_ is the energy of single gaseous metal atom, *E*_M,bulk_ is the energy of metal unit cell, and n is the number of metal unit cell.***Table S6***. Computed BE, *E*_coh-ave_ and aggregation energy (*E*_agg_) of M_2_ dimer embeded in N-doped graphene, where *E*_coh-ave_ = 1/2(*E*_coh-Fe_ + *E*_coh-M_) and E_agg_ = BE - *E*_coh-ave_. Bolded fonts are DACs synthesized for the experiment.

| FeM | BE | *E*_coh-ave_ | *E*_agg_ |
| --- | --- | --- | --- |
| Ti | -6.98 | -5.08 | -1.90 |
| V | -6.09 | -5.11 | -0.98 |
| Cr | -5.17 | -4.46 | -0.70 |
| Mn | -5.19 | -4.35 | -0.84 |
| **Fe** | **-5.34** | **-4.86** | **-0.49** |
| **Co** | **-5.96** | **-4.89** | **-1.07** |
| **Ni** | **-5.92** | **-4.83** | **-1.08** |
| **Cu** | **-5.06** | **-4.18** | **-0.88** |
| Zr | -7.75 | -5.51 | -2.24 |
| Nb | -7.01 | -5.93 | -1.08 |
| Mo | -5.81 | -5.57 | -0.24 |
| Ru | -6.03 | -5.81 | -0.23 |
| Rh | -6.16 | -5.42 | -0.74 |
| Pd | -4.76 | -4.30 | -0.46 |
| Ag | -3.93 | -3.69 | -0.24 |
| Hf | -7.86 | -5.64 | -2.22 |
| Ta | -7.43 | -6.48 | -0.95 |
| W | -6.58 | -6.62 | 0.04 |
| Re | -6.29 | -6.34 | 0.05 |
| Os | -6.50 | -6.59 | 0.10 |
| Ir | -6.64 | -6.08 | -0.56 |
| Pt | -5.77 | -5.21 | -0.56 |
| Au | -4.37 | -3.95 | -0.43 |

***Table S7***. Computed BE, *E*_coh-ave_ and aggregation energy (*E*_agg_) of M_2_ dimer embeded in N-doped graphene, where *E*_coh-ave_ = 1/2(*E*_coh-Co_ + *E*_coh-M_) and *E*_agg_ = BE - *E*_coh-ave_. Bolded fonts are DACs synthesized for the experiment.

| CoM | BE | *E*_coh-ave_ | *E*_agg_ |
| --- | --- | --- | --- |
| Ti | -7.26 | -5.11 | -2.14 |
| V | -6.45 | -5.14 | -1.31 |
| Cr | -5.59 | -4.50 | -1.10 |
| Mn | -5.54 | -4.38 | -1.16 |
| Co | -6.03 | -4.92 | -1.11 |
| **Ni** | **-6.10** | **-4.87** | **-1.23** |
| Cu | -5.33 | -4.21 | -1.11 |
| Zr | -8.07 | -5.54 | -2.53 |
| Nb | -7.35 | -5.96 | -1.39 |
| Mo | -6.00 | -5.60 | -0.40 |
| Ru | -5.47 | -5.84 | 0.37 |
| Rh | -5.65 | -5.45 | -0.19 |
| Pd | -4.79 | -4.34 | -0.45 |
| Ag | -4.21 | -3.72 | -0.49 |
| Hf | -8.18 | -5.67 | -2.51 |
| Ta | -7.80 | -6.52 | -1.28 |
| W | -6.81 | -6.66 | -0.15 |
| Re | -6.33 | -6.37 | 0.04 |
| Os | -5.92 | -6.63 | 0.71 |
| Ir | -5.20 | -6.11 | 0.91 |
| Pt | -5.79 | -5.24 | -0.55 |
| Au | -4.65 | -3.98 | -0.67 |

***Table S8***. Computed BE, *E*_coh-ave_ and aggregation energy (*E*_agg_) of M_2_ dimer embeded in N-doped graphene, where *E*_coh-ave_ = 1/2(*E*_coh-Ni_ + *E*_coh-M_) and *E*_agg_ = BE - *E*_coh-ave_. Bolded fonts are DACs synthesized for the experiment.

| NiM | BE | *E*_coh-ave_ | *E*_agg_ |
| --- | --- | --- | --- |
| Ti | -7.37 | -5.06 | -2.31 |
| V | -6.56 | -5.08 | -1.48 |
| Cr | -5.67 | -4.44 | -1.23 |
| Mn | -5.75 | -4.32 | -1.43 |
| **Ni** | **-6.27** | **-4.81** | **-1.46** |
| **Cu** | **-5.36** | **-4.16** | **-1.20** |
| Zr | -8.19 | -5.49 | -2.70 |
| Nb | -7.28 | -5.90 | -1.37 |
| Mo | -5.88 | -5.55 | -0.33 |
| Ru | -6.86 | -5.78 | -1.08 |
| Rh | -6.83 | -5.40 | -1.43 |
| Pd | -5.90 | -4.28 | -1.62 |
| Ag | -4.26 | -3.66 | -0.60 |
| Hf | -8.31 | -5.61 | -2.70 |
| Ta | -7.71 | -6.46 | -1.25 |
| W | -6.59 | -6.60 | 0.01 |
| Re | -6.16 | -6.31 | 0.15 |
| Os | -7.23 | -6.57 | -0.66 |
| Ir | -7.30 | -6.05 | -1.25 |
| Pt | -6.91 | -5.18 | -1.72 |
| Au | -4.77 | -3.92 | -0.84 |

***Table S9***. Computed BE, *E*_coh-ave_ and aggregation energy (*E*_agg_) of M_2_ dimer embeded in N-doped graphene, where *E*_coh-ave_ = 1/2(*E*_coh-Cu_ + *E*_coh-M_) and *E*_agg_ = BE - *E*_coh-ave_.

| CuM | BE | *E*_coh-ave_ | *E*_agg_ |
| --- | --- | --- | --- |
| Ti | -6.49 | -4.40 | -2.09 |
| V | -5.72 | -4.43 | -1.29 |
| Cr | -4.99 | -3.79 | -1.21 |
| Mn | -4.78 | -3.67 | -1.11 |
| Cu | -4.54 | -3.51 | -1.04 |
| Zr | -7.30 | -4.83 | -2.46 |
| Nb | -6.31 | -5.25 | -1.06 |
| Mo | -4.93 | -4.89 | -0.03 |
| Ru | -5.33 | -5.13 | -0.20 |
| Rh | -5.63 | -4.75 | -0.88 |
| Pd | -4.59 | -3.63 | -0.96 |
| Ag | -3.49 | -3.01 | -0.48 |
| Hf | -7.43 | -4.96 | -2.47 |
| Ta | -6.68 | -5.81 | -0.88 |
| W | -5.54 | -5.95 | 0.41 |
| Re | -5.19 | -5.66 | 0.47 |
| Os | -5.73 | -5.92 | 0.19 |
| Ir | -3.70 | -5.40 | 1.70 |
| Pt | -5.58 | -4.53 | -1.05 |
| Au | -3.70 | -3.27 | -0.43 |

***Table S10***. Computed total energy of FeM@NC, where *E*_NC_ = 620.53 eV. For comparison, the standard dissolution potential (*U*) of metal atoms, the number of transferred electrons (*N*_e_) during the dissolution and dissolution potential (*U*_diss_) are also listed.

| FeM | *U* | *E*_FeM@NC_ | *N*_e_ | *U*_diss_ |
| --- | --- | --- | --- | --- |
| Ti | -1.63 | -640.40 | 2 | 0.86 |
| V | -1.18 | -639.72 | 2 | 0.17 |
| Cr | -0.91 | -639.69 | 2 | 0.02 |
| Mn | -1.19 | -639.44 | 2 | 0.02 |
| Fe | -0.45 | -637.98 | 2 | 0.04 |
| Co | -0.28 | -637.73 | 2 | 0.70 |
| Ni | -0.26 | -636.35 | 2 | 0.73 |
| Cu | 0.34 | -634.26 | 2 | 0.83 |
| Zr | -1.45 | -641.78 | 4 | 0.55 |
| Nb | -1.10 | -641.14 | 3 | 0.09 |
| Mo | -0.20 | -640.18 | 3 | -0.14 |
| Ru | 0.46 | -638.46 | 2 | 0.23 |
| Rh | 0.60 | -637.50 | 2 | 0.82 |
| Pd | 0.95 | -634.91 | 2 | 0.71 |
| Ag | 0.80 | -631.97 | 1 | 0.50 |
| Hf | -1.55 | -643.14 | 4 | 0.48 |
| Ta | -0.60 | -642.48 | 3 | 0.23 |
| W | 0.10 | -641.64 | 3 | -0.21 |
| Re | 0.30 | -641.11 | 3 | -0.11 |
| Os | 0.84 | -639.83 | 8 | 0.16 |
| Ir | 1.16 | -638.75 | 3 | 0.80 |
| Pt | 1.18 | -635.99 | 2 | 0.93 |
| Au | 1.50 | -632.84 | 3 | 0.87 |

***Table S11***. Computed total energy of CoM@NC, where *E*_NC_ = 620.53 eV. For comparison, the standard dissolution potential (*U*) of metal atoms, the number of transferred electrons (*N*_e_) during the dissolution and dissolution potential (*U*_diss_) are also listed.

| CoM | *U* | *E*_CoM@NC_ | *N*_e_ | *U*_diss_ |
| --- | --- | --- | --- | --- |
| Ti | -1.63 | -639.47 | 2 | 1.19 |
| V | -1.18 | -638.96 | 2 | 0.58 |
| Cr | -0.91 | -639.05 | 2 | 0.50 |
| Mn | -1.19 | -638.67 | 2 | 0.43 |
| Co | -0.28 | -636.38 | 2 | 0.83 |
| Ni | -0.26 | -635.23 | 2 | 0.96 |
| Cu | 0.34 | -633.31 | 2 | 1.14 |
| Zr | -1.45 | -640.93 | 4 | 0.82 |
| Nb | -1.10 | -640.34 | 3 | 0.42 |
| Mo | -0.20 | -639.09 | 3 | 0.08 |
| Ru | 0.46 | -635.86 | 2 | -0.28 |
| Rh | 0.60 | -634.99 | 2 | 0.36 |
| Pd | 0.95 | -633.48 | 2 | 0.79 |
| Ag | 0.80 | -631.05 | 1 | 0.91 |
| Hf | -1.55 | -642.30 | 4 | 0.76 |
| Ta | -0.60 | -641.73 | 3 | 0.58 |
| W | 0.10 | -640.60 | 3 | 0.03 |
| Re | 0.30 | -639.70 | 3 | -0.02 |
| Os | 0.84 | -637.18 | 8 | 0.00 |
| Ir | 1.16 | -634.39 | 3 | -0.29 |
| Pt | 1.18 | -634.55 | 2 | 1.00 |
| Au | 1.50 | -631.91 | 3 | 1.15 |

***Table S12***. Computed total energy of NiM@NC, where *E*_NC_ = 620.53 eV. For comparison, the standard dissolution potential (*U*) of metal atoms, the number of transferred electrons (*N*_e_) during the dissolution and dissolution potential (*U*_diss_) are also listed.

| NiM | *U* | *E*_NiM@NC_ | *N*_e_ | *U*_diss_ |
| --- | --- | --- | --- | --- |
| Ti | -1.63 | -638.40 | 2 | 1.19 |
| V | -1.18 | -637.89 | 2 | 0.58 |
| Cr | -0.91 | -637.92 | 2 | 0.50 |
| Mn | -1.19 | -637.80 | 2 | 0.43 |
| Ni | -0.26 | -634.29 | 2 | 0.83 |
| Cu | 0.34 | -632.08 | 2 | 0.96 |
| Zr | -1.45 | -639.87 | 4 | 1.14 |
| Nb | -1.10 | -638.90 | 3 | 0.82 |
| Mo | -0.20 | -637.54 | 3 | 0.42 |
| Ru | 0.46 | -637.34 | 2 | 0.08 |
| Rh | 0.60 | -636.07 | 2 | -0.28 |
| Pd | 0.95 | -634.40 | 2 | 0.36 |
| Ag | 0.80 | -629.85 | 1 | 0.79 |
| Hf | -1.55 | -641.28 | 4 | 0.91 |
| Ta | -0.60 | -640.25 | 3 | 0.76 |
| W | 0.10 | -638.88 | 3 | 0.58 |
| Re | 0.30 | -638.06 | 3 | 0.03 |
| Os | 0.84 | -638.52 | 8 | -0.02 |
| Ir | 1.16 | -637.29 | 3 | 0.00 |
| Pt | 1.18 | -635.48 | 2 | -0.29 |
| Au | 1.50 | -630.85 | 3 | 1.00 |

***Table S13***. Computed total energy of CuM@NC, where *E*_NC_ = 620.53 eV. For comparison, the standard dissolution potential (*U*) of metal atoms, the number of transferred electrons (*N*_e_) during the dissolution and dissolution potential (*U*_diss_) are also listed.

| CuM | *U* | *E*_CuM@NC_ | *N*_e_ | *U*_diss_ |
| --- | --- | --- | --- | --- |
| Ti | -1.63 | -636.27 | 2 | 1.44 |
| V | -1.18 | -635.84 | 2 | 0.87 |
| Cr | -0.91 | -636.18 | 2 | 0.92 |
| Mn | -1.19 | -635.47 | 2 | 0.68 |
| Cu | 0.34 | -630.07 | 2 | 1.38 |
| Zr | -1.45 | -637.71 | 4 | 1.09 |
| Nb | -1.10 | -636.60 | 3 | 0.47 |
| Mo | -0.20 | -635.25 | 3 | 0.09 |
| Ru | 0.46 | -633.90 | 2 | 0.60 |
| Rh | 0.60 | -633.28 | 2 | 1.36 |
| Pd | 0.95 | -631.40 | 2 | 1.61 |
| Ag | 0.80 | -627.93 | 1 | 1.20 |
| Hf | -1.55 | -639.13 | 4 | 1.04 |
| Ta | -0.60 | -637.83 | 3 | 0.57 |
| W | 0.10 | -636.40 | 3 | -0.11 |
| Re | 0.30 | -635.76 | 3 | -0.05 |
| Os | 0.84 | -635.13 | 8 | 0.51 |
| Ir | 1.16 | -629.72 | 3 | -0.61 |
| Pt | 1.18 | -632.46 | 2 | 1.81 |
| Au | 1.50 | -628.34 | 3 | 1.26 |

***Table S14***. Adsorption free energy of FeM@NC for *OH, Bold fonts represent the most stable configuration.

| **FeM** | 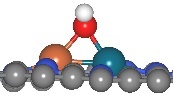 | 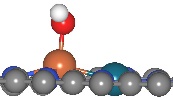 | 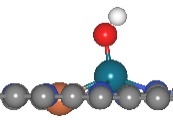 |
| --- | --- | --- | --- |
| FeTi | **-0.98** | / | -0.92 |
| FeV | -1.29 | / | **-1.56** |
| FeCr | **-1.42** | / | / |
| FeMn | **-1.06** | / | / |
| Fe_2_ | **-0.97** | / | / |
| FeCo | **-0.23** | 0.02 | / |
| FeNi | -0.19 | **-0.23** | / |
| FeCu | / | **-0.35** | / |
| FeZr | **-0.97** | / | -0.78 |
| FeNb | -0.94 | / | **-1.23** |
| FeMo | -0.78 | / | **-1.30** |
| FeRu | **-0.56** | 0.00 | / |
| FeRh | **-0.15** | / | / |
| FePd | / | **-0.81** | / |
| FeAg | **-0.75** | -0.38 | / |
| FeHf | **-1.18** | / | -1.03 |
| FeTa | -1.15 | / | **-1.56** |
| FeW | -0.81 | / | **-1.57** |
| FeRe | / | 0.39 | **-0.83** |
| FeOs | **-0.54** | -0.03 | / |
| FeIr | -0.03 | **-0.22** | 1.01 |
| FePt | -0.39 | **-0.93** | / |
| FeAu | 0.32 | **-0.25** | / |

***Table S15***. The calculated relative energy of the optimized adsorption configuration for *OH-*OH, Bold fonts represent the most stable configuration.

| **FeM** | 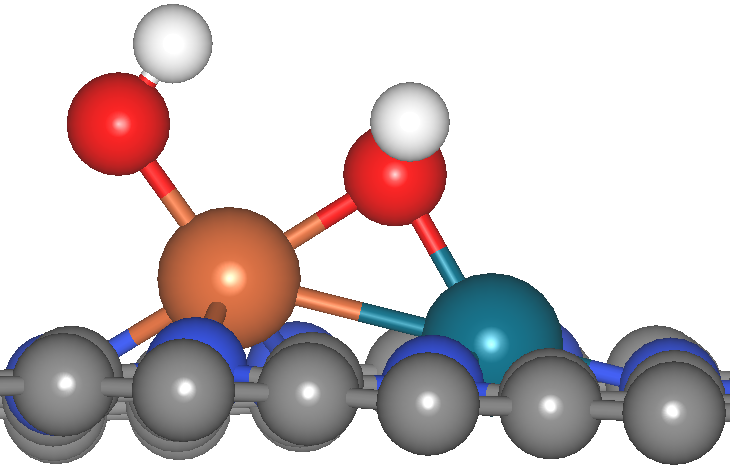 | 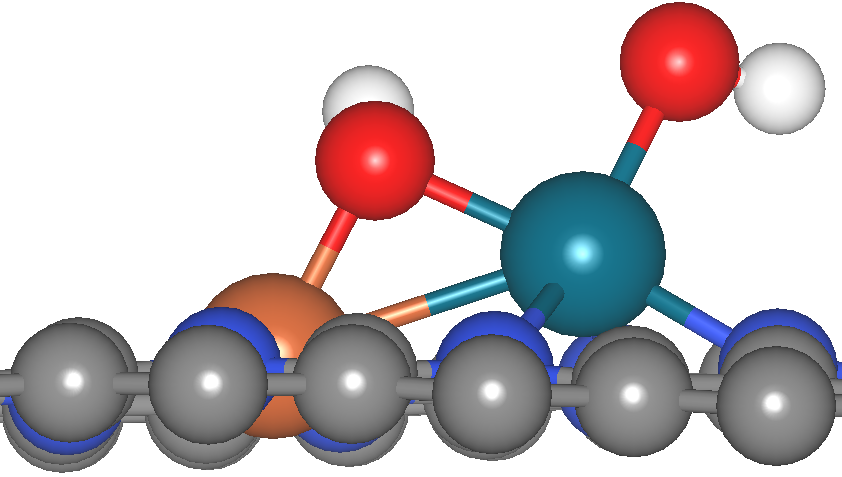 |
| --- | --- | --- |
| FeTi | 0.00 | **-0.71** |
| FeCr | 0.00 | **-0.22** |
| FeMn | 0.00 | **-0.20** |
| FeCo | 0.00 | **-0.43** |
| FeZr | 0.00 | **-0.47** |
| FeRu | 0.00 | **-0.07** |
| FeRh | **-0.59** | 0.00 |
| FeAg | **-0.19** | 0.00 |
| FeHf | 0.00 | **-0.63** |
| FeOs | 0.00 | **-0.21** |

***Table S16***. The calculated relative energy of the optimized adsorption configuration for *OH-*OH, Bold fonts represent the most stable configuration.

| **FeM** | 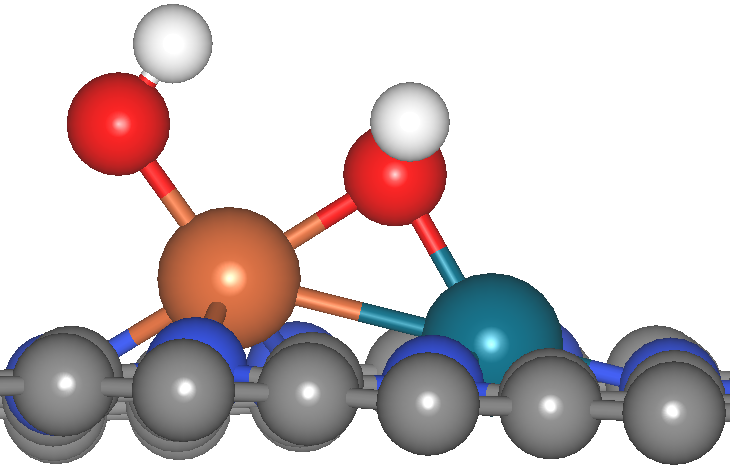 | 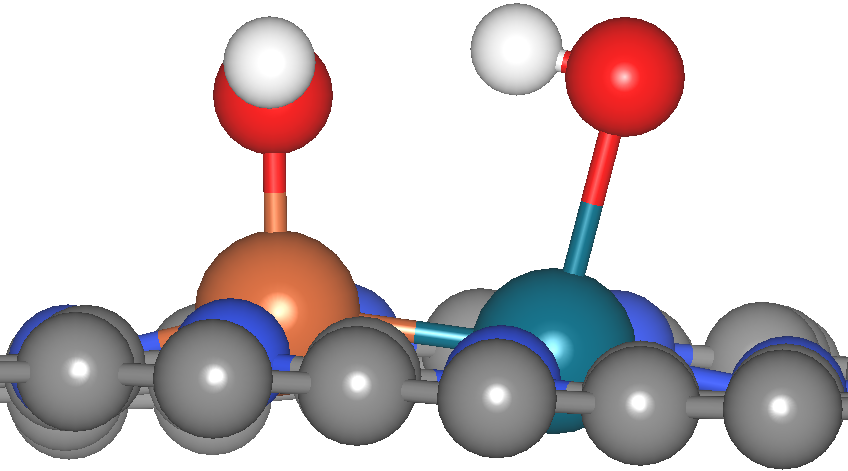 |
| --- | --- | --- |
| FeNi | **0.00** | / |
| FeCu | **0.00** | / |
| FePd | **0.00** | / |
| FeIr | **0.00** | / |
| FePt | **0.00** | / |
| FeAu | **0.00** | / |

***Table S17***. The calculated relative energy of the optimized adsorption configuration for *OH-*OH, Bold fonts represent the most stable configuration.

| **FeM** | 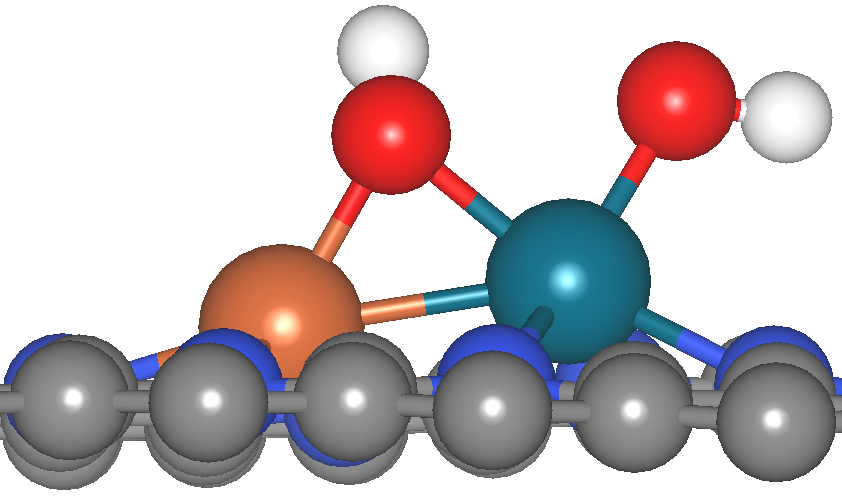 | 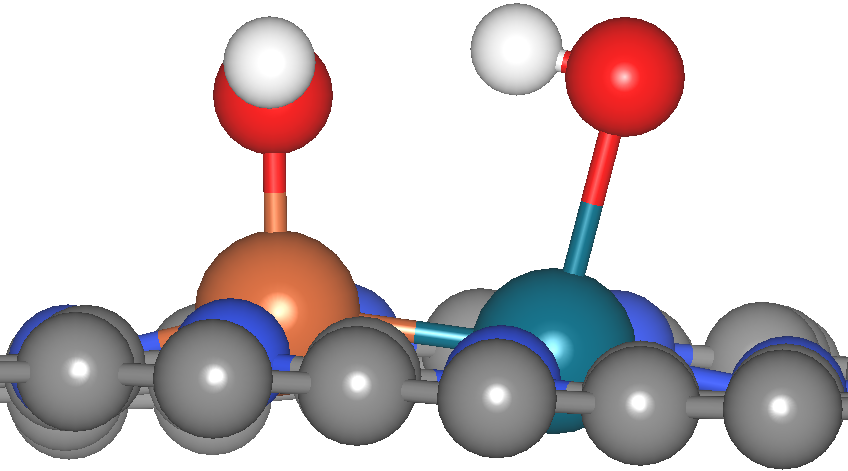 |
| --- | --- | --- |
| FeV | **0.00** | **/** |
| FeNb | **0.00** | **/** |
| FeMo | **0.00** | **/** |
| FeTa | **0.00** | **/** |
| FeW | **0.00** | **/** |
| FeRe | **/** | **0.00** |

***Table S18***. Adsorption free energy of CoM@NC for *OH, Bold fonts represent the most stable configuration.

| **CoM** | 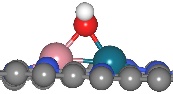 | 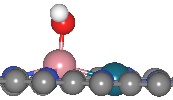 | 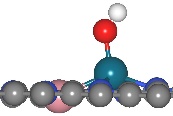 |
| --- | --- | --- | --- |
| CoTi | -0.82 | / | **-0.88** |
| CoV | -0.99 | / | **-1.29** |
| CoCr | **-1.09** | / | -0.70 |
| CoMn | **-0.66** | / | / |
| Co_2_ | **-0.50** | / | / |
| CoNi | / | **0.19** | / |
| CoCu | **0.14** | / | / |
| CoZr | **-0.84** | / | -0.72 |
| CoNb | -0.33 | / | **-1.20** |
| CoMo | -0.87 | / | **-1.49** |
| CoRu | **-0.50** | / | -0.28 |
| CoRh | **-0.45** | / | -0.02 |
| CoPd | 0.17 | **0.13** | / |
| CoAg | **0.02** | 0.12 | 0.45 |
| CoHf | **-1.04** | / | -0.76 |
| CoTa | -0.58 | / | **-1.49** |
| CoW | / | / | **-1.85** |
| CoRe | / | 0.52 | **-1.13** |
| CoOs | **-0.69** | 0.31 | -0.67 |
| CoIr | **-0.44** | 0.10 | / |
| CoPt | 0.28 | **0.16** | / |
| CoAu | / | **0.37** | 1.53 |

***Table S19***. The calculated relative energy of the optimized adsorption configuration for *OH-*OH, Bold fonts represent the most stable configuration.

| **CoM** | 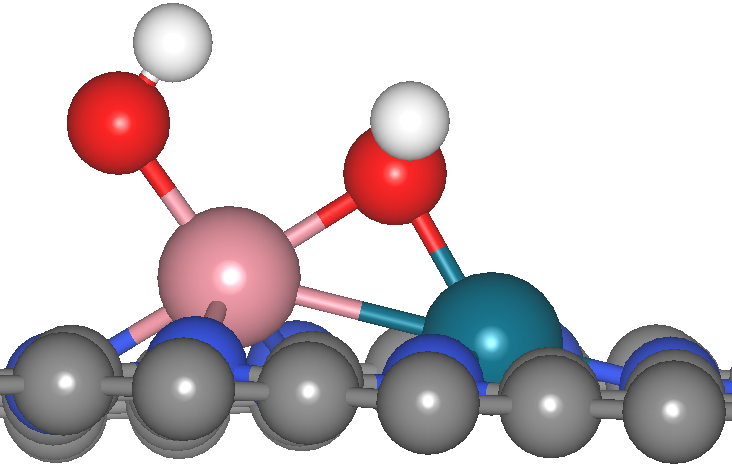 | 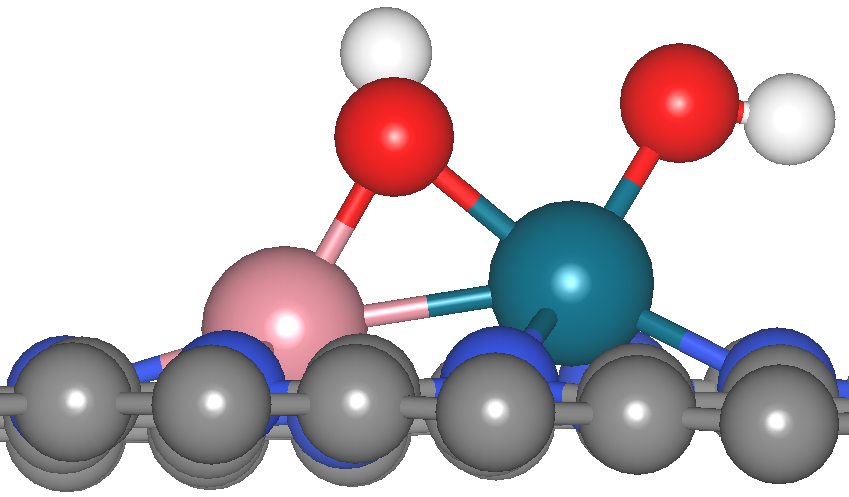 |
| --- | --- | --- |
| CoCr | 0.00 | **-0.67** |
| CoMn | 0.00 | **-0.53** |
| CoCu | 0.00 | **-0.53** |
| CoZr | 0.00 | **-0.35** |
| CoRu | 0.00 | **-0.67** |
| CoRh | 0.00 | **-0.07** |
| CoAg | **-0.28** | 0.00 |
| CoHf | 0.00 | **-0.53** |
| CoOs | 0.00 | **-0.78** |
| CoIr | 0.00 | **-0.26** |

***Table S20***. The calculated relative energy of the optimized adsorption configuration for *OH-*OH, Bold fonts represent the most stable configuration.

| **CoM** | 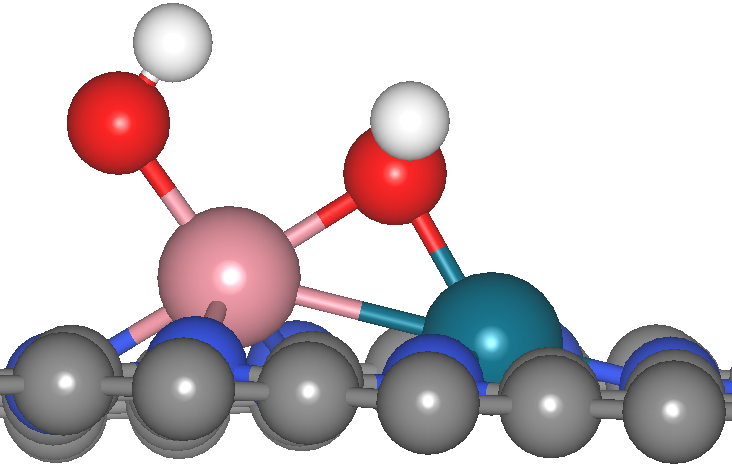 | 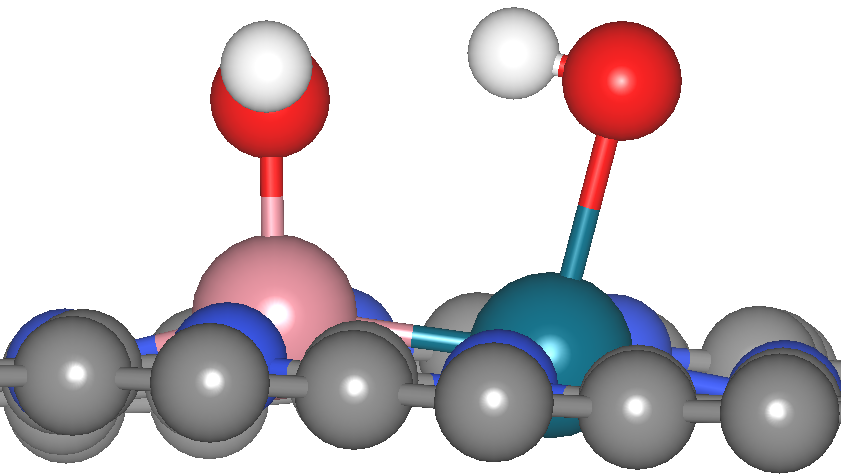 |
| --- | --- | --- |
| CoNi | **-0.46** | 0.00 |
| CoPd | **-0.74** | 0.00 |
| CoPt | **-0.61** | 0.00 |
| CoAu | **/** | **0.00** |

***Table S21***. The calculated relative energy of the optimized adsorption configuration for *OH-*OH, Bold fonts represent the most stable configuration.

| **CoM** | 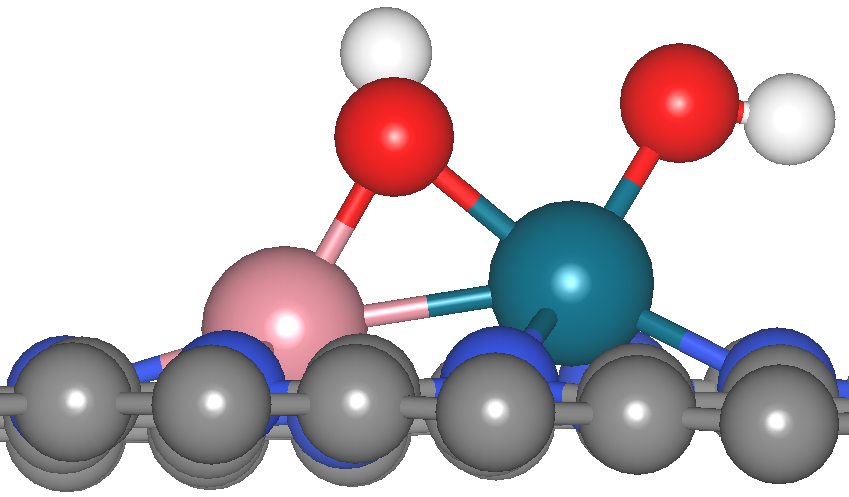 | 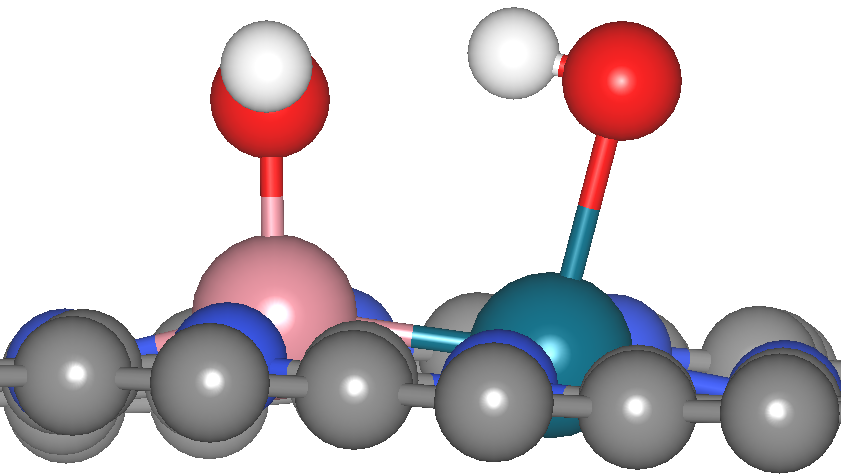 |
| --- | --- | --- |
| CoTi | **0.00** | **/** |
| CoV | **0.00** | **/** |
| CoNb | **0.00** | **/** |
| CoMo | **0.00** | **/** |
| CoTa | **0.00** | **/** |
| CoW | **0.00** | **/** |
| CoRe | **0.00** | **/** |

***Table S22***. Adsorption free energy of NiM@NC for *OH, Bold fonts represent the most stable configuration.

| **NiM** | 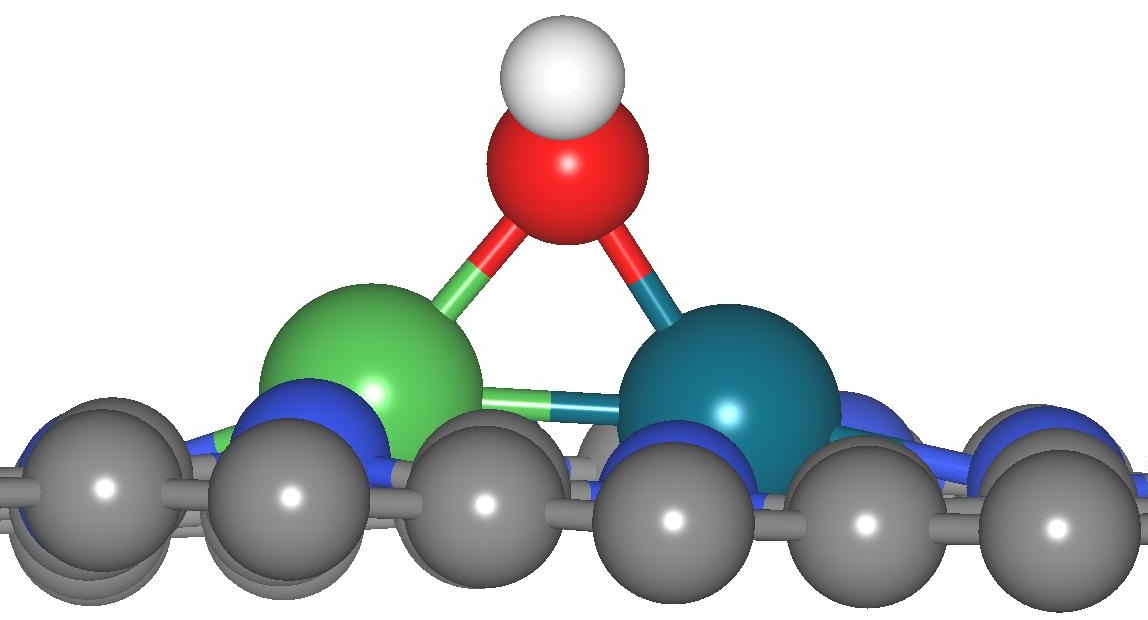 | 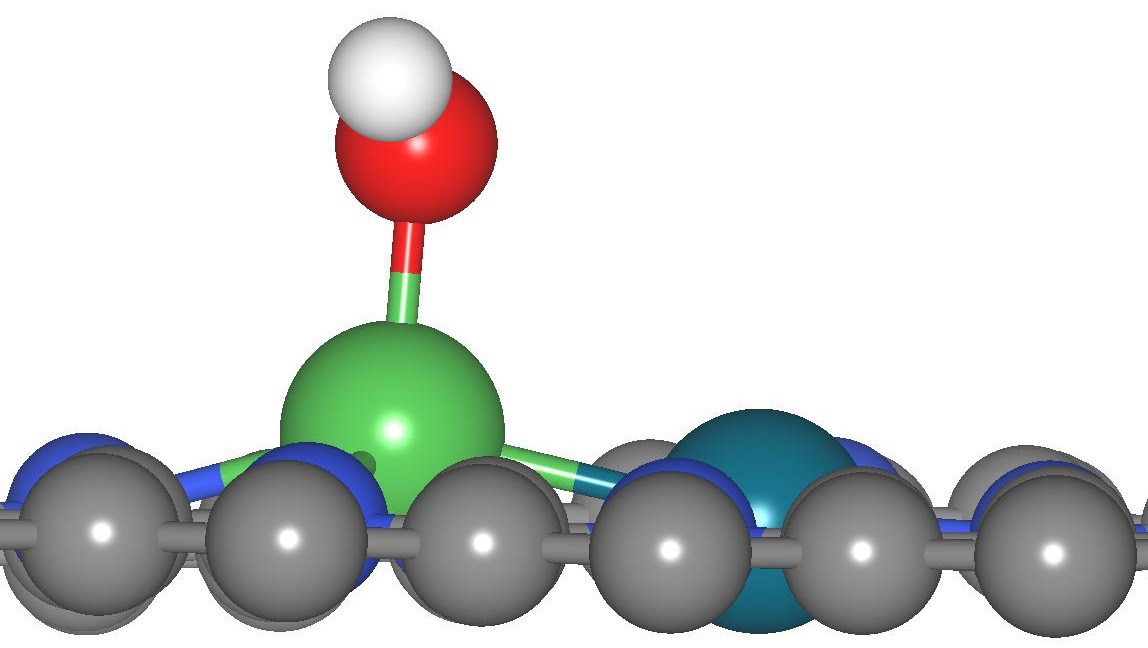 | 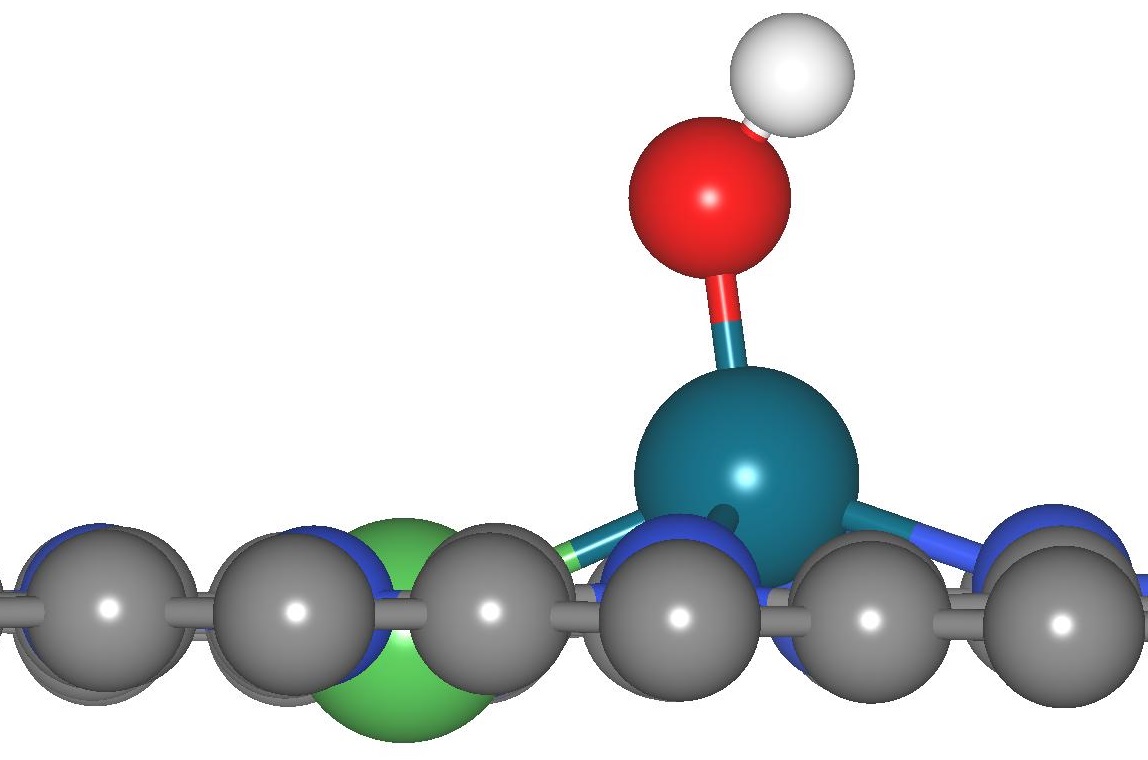 |
| --- | --- | --- | --- |
| NiTi | -0.20 | / | **-1.03** |
| NiV | -0.53 | / | **-1.28** |
| NiCr | -0.60 | / | **-0.78** |
| NiMn | / | / | **-0.24** |
| Ni_2_ | **0.73** | / | / |
| NiCu | **0.59** | / | / |
| NiZr | -0.23 | / | **-0.67** |
| NiNb | -0.19 | / | **-1.37** |
| NiMo | / | / | **-1.71** |
| NiRu | / | / | **-0.53** |
| NiRh | **0.17** | / | 0.68 |
| NiPd | **0.92** | / | / |
| NiAg | **0.31** | / | 0.60 |
| NiHf | -0.48 | / | **-0.76** |
| NiTa | -0.44 | / | **-1.83** |
| NiW | / | / | **-2.22** |
| NiRe | / | / | **-1.53** |
| NiOs | / | / | **-0.70** |
| NiIr | **0.14** | 0.77 | 0.79 |
| NiPt | 1.02 | **1.01** | / |
| NiAu | / | **1.04** | 1.48 |

***Table S23***. The calculated relative energy of the optimized adsorption configuration for *OH-*OH, Bold fonts represent the most stable configuration.

| **NiM** | 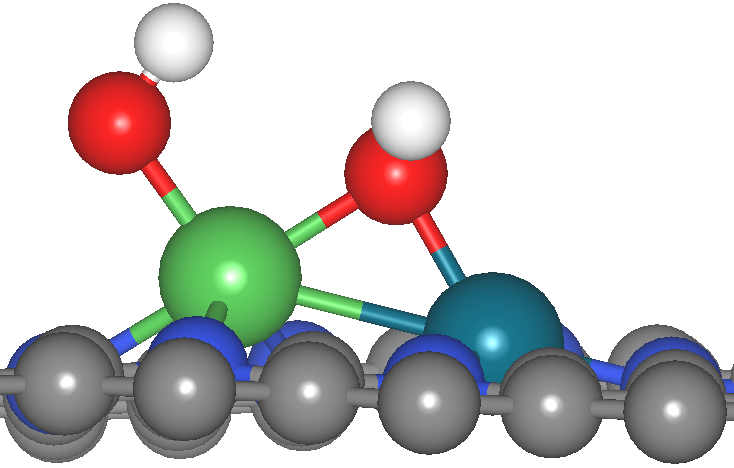 | 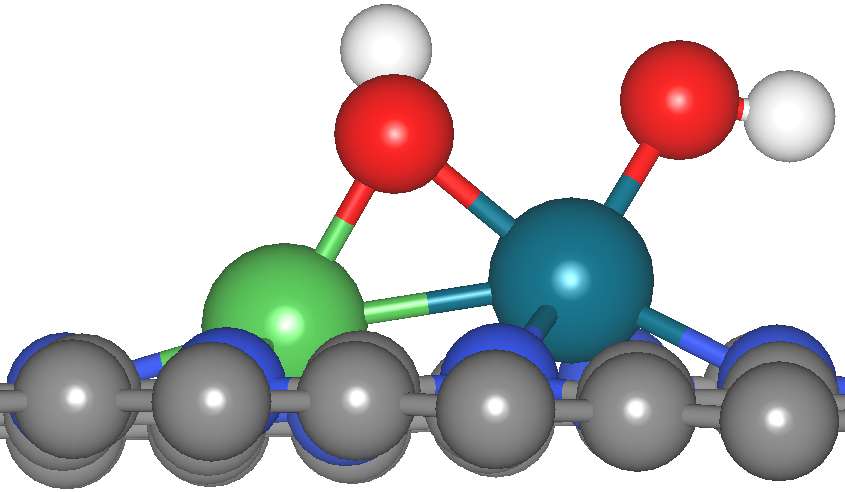 |
| --- | --- | --- |
| NiCu | **-0.44** | 0.00 |
| NiRh | **-0.15** | 0.00 |
| NiPd | **-0.35** | 0.00 |
| NiAg | **-0.63** | 0.00 |
| NiIr | 0.00 | **-0.36** |

***Table S24***. The calculated relative energy of the optimized adsorption configuration for *OH-*OH, Bold fonts represent the most stable configuration.

| **NiM** | 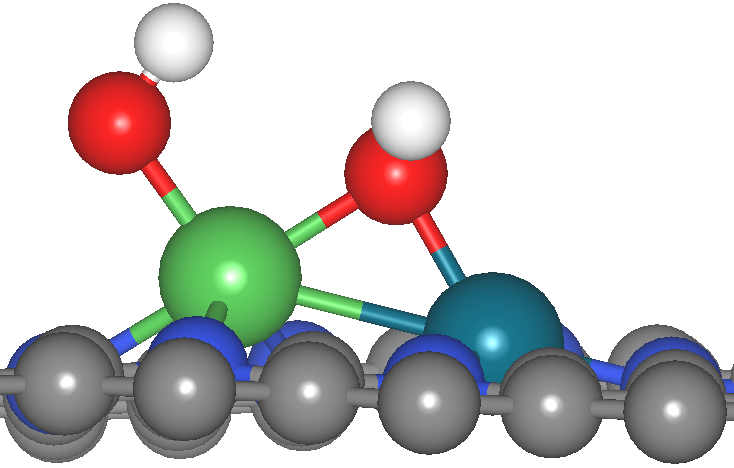 | 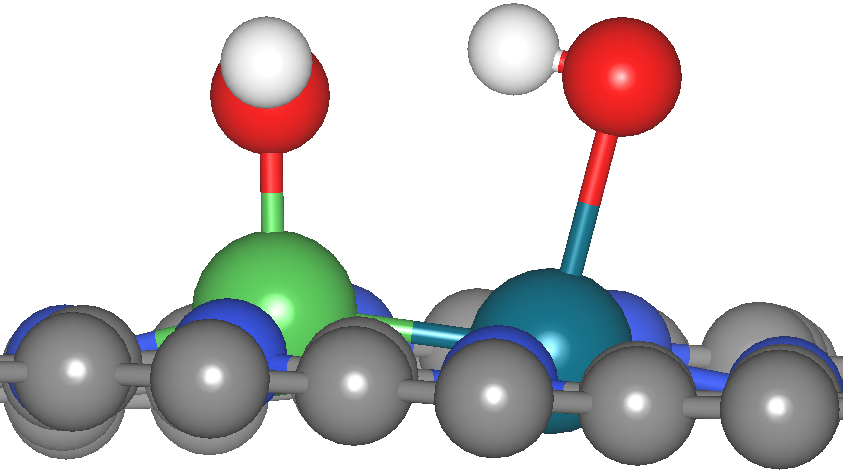 |
| --- | --- | --- |
| NiAu | **-0.84** | 0.00 |
| NiPt | **-0.49** | 0.00 |

***Table S25***. The calculated relative energy of the optimized adsorption configuration for *OH-*OH, Bold fonts represent the most stable configuration.

| **NiM** | 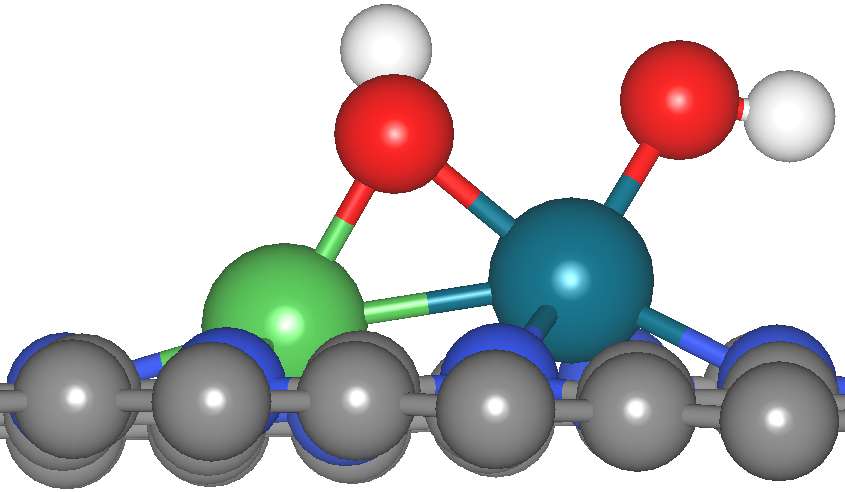 | 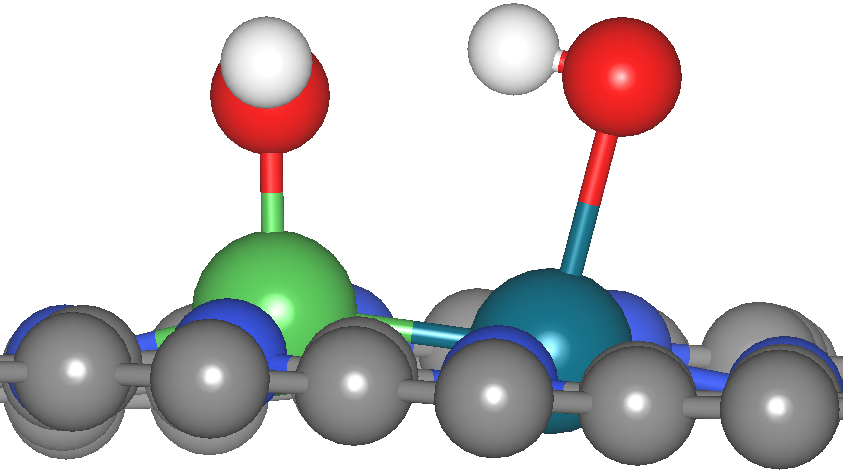 |
| --- | --- | --- |
| NiTi | **0.00** | **/** |
| NiV | **0.00** | **/** |
| NiCr | **0.00** | **/** |
| NiMn | 0.00 | **-0.66** |
| NiZr | 0.00 | **-0.25** |
| NiNb | **0.00** | **/** |
| NiMo | **-0.13** | 0.00 |
| NiRu | **0.00** | **/** |
| NiHf | **0.00** | **/** |
| NiTa | **0.00** | **/** |
| NiW | **0.00** | **/** |
| NiRe | **0.00** | **/** |
| NiOs | **0.00** | **/** |

***Table S26***. Adsorption free energy of CuM@NC for *OH, Bold fonts represent the most stable configuration.

| **CuM** | 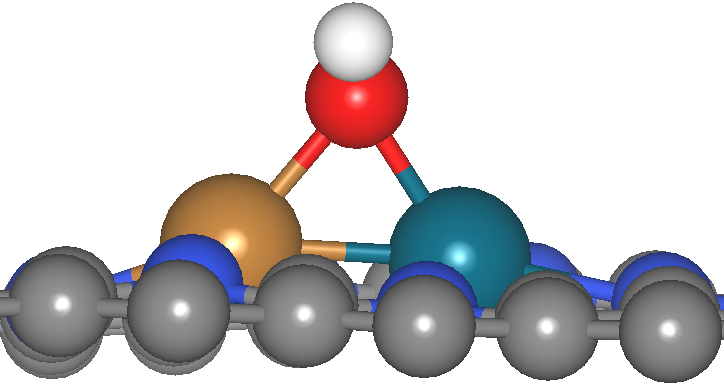 | 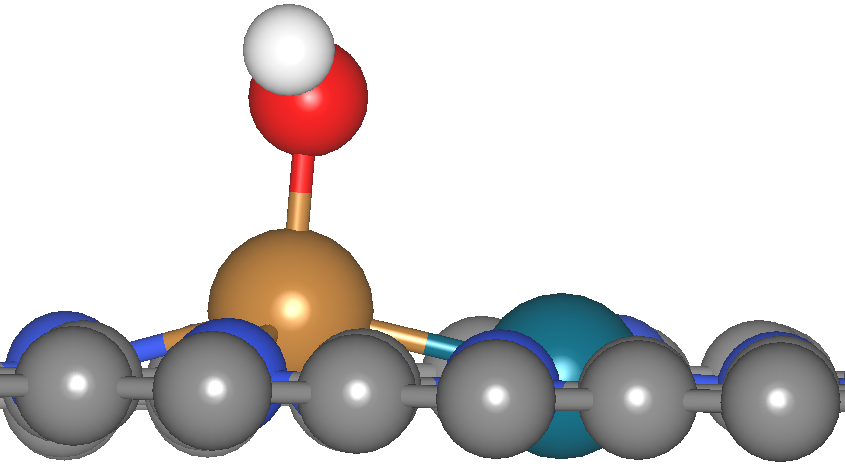 | 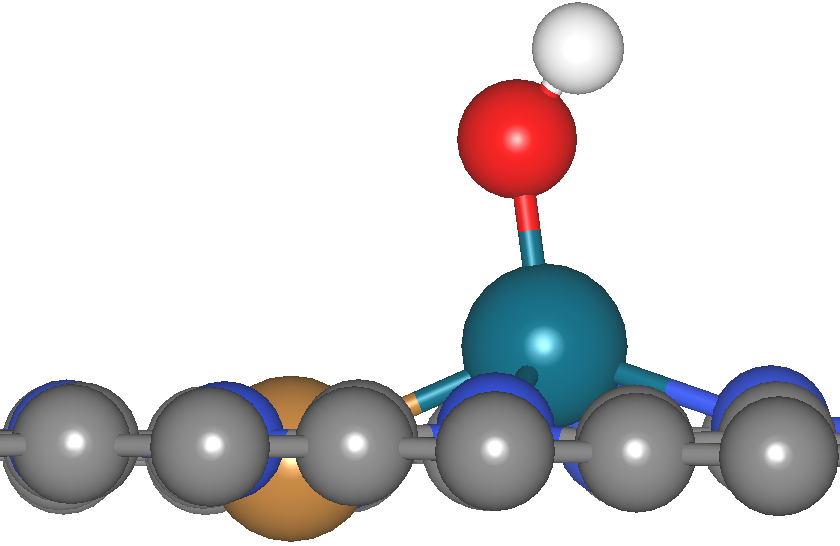 |
| --- | --- | --- | --- |
| CuTi | **-0.43** | / | / |
| CuV | / | / | **-1.27** |
| CuCr | / | / | **-0.58** |
| CuMn | / | / | **-0.59** |
| Cu2 | **0.58** | / | / |
| CuZr | -0.55 | / | **-1.08** |
| CuNb | / | / | **-1.38** |
| CuMo | -0.91 | / | **-1.55** |
| CuRu | / | / | **-0.33** |
| CuRh | **0.59** | / | / |
| CuPd | **0.83** | / | / |
| CuAg | **0.36** | / | 0.56 |
| CuHf | -0.78 | / | **-1.26** |
| CuTa | / | / | **-1.75** |
| CuW | / | / | **-2.13** |
| CuRe | / | / | **-1.43** |
| CuOs | / | 0.58 | **-0.55** |
| CuIr | **0.49** | 1.03 | / |
| CuPt | **0.88** | / | / |
| CuAu | / | **0.80** | / |

***Table S27***. The calculated relative energy of the optimized adsorption configuration for *OH-*OH, Bold fonts represent the most stable configuration.

| **CuM** | 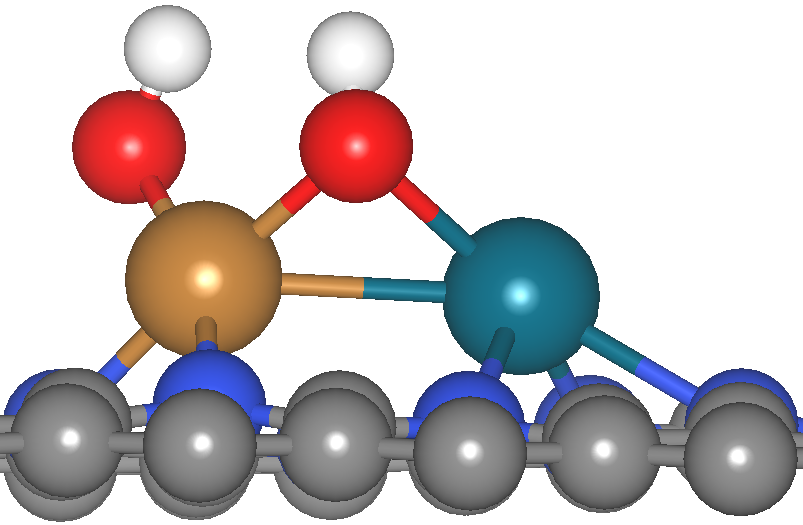 | 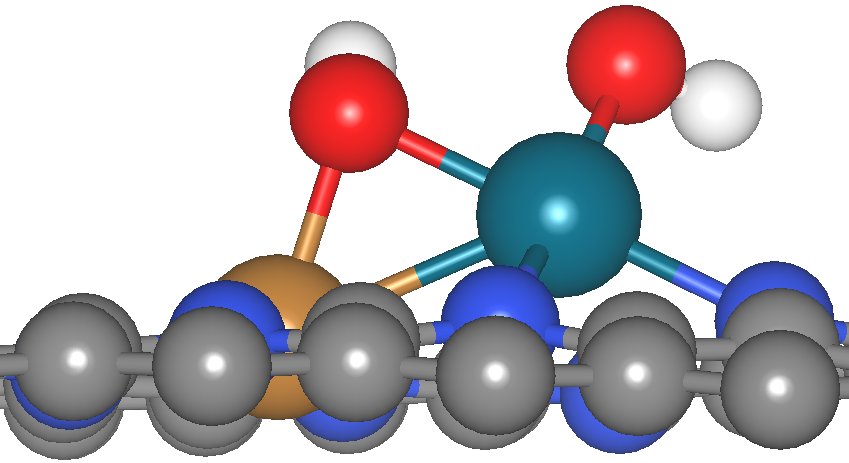 |
| --- | --- | --- |
| CuTi | 0.00 | **-0.22** |
| CuRh | 0.00 | **-0.12** |
| CuPd | **-0.01** | 0.00 |
| CuAg | **-0.24** | 0.00 |
| CuIr | 0.00 | **-0.60** |
| CuPt | 0.00 | **-0.01** |

***Table S28***. The calculated relative energy of the optimized adsorption configuration for *OH-*OH, Bold fonts represent the most stable configuration.

| **CuM** | 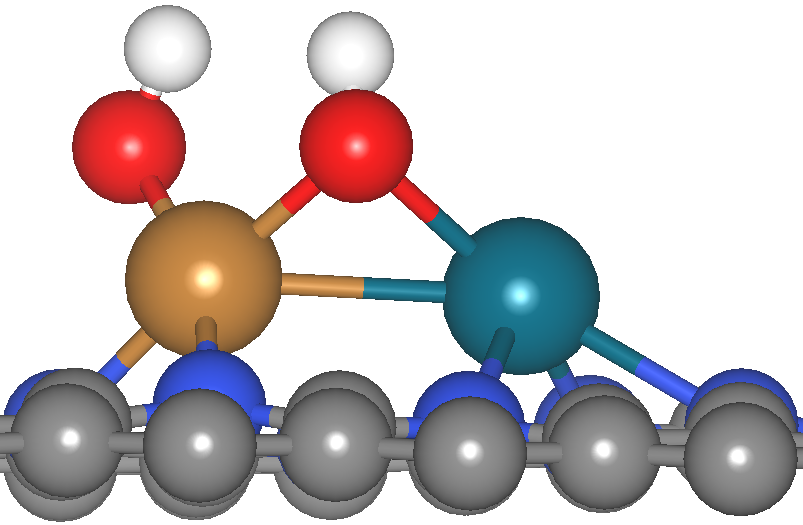 | 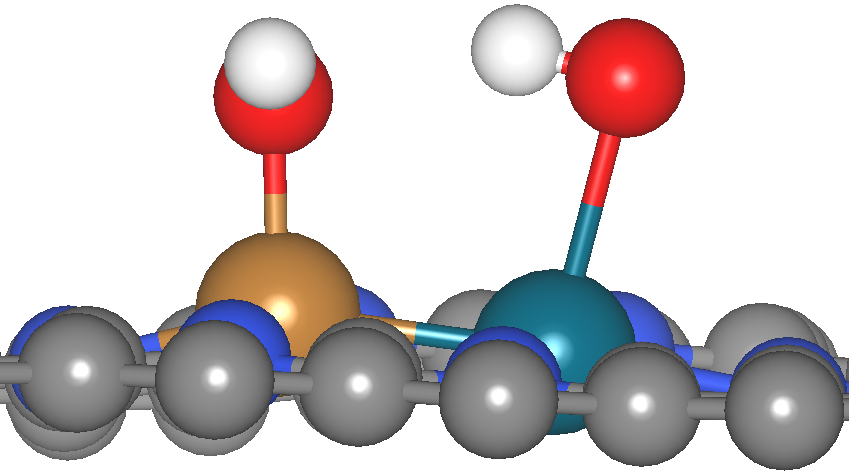 |
| --- | --- | --- |
| CuAu | **-0.44** | 0.00 |

***Table S29***. The calculated relative energy of the optimized adsorption configuration for *OH-*OH, Bold fonts represent the most stable configuration.

| **CuM** | 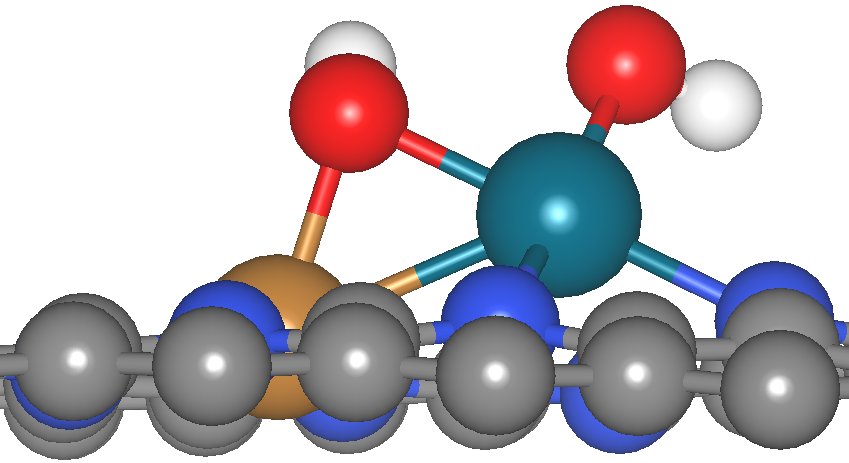 | 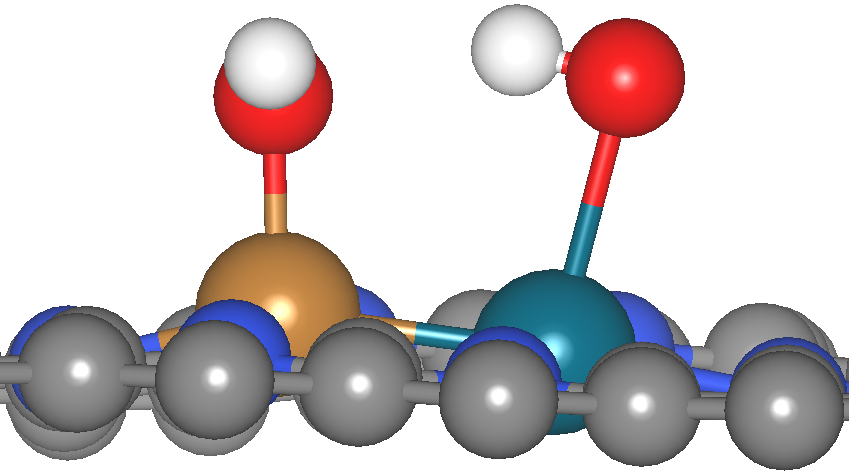 |
| --- | --- | --- |
| CuV | **0.00** | **/** |
| CuCr | **-0.87** | 0.00 |
| CuMn | **0.00** | **/** |
| CuZr | **0.00** | **/** |
| CuNb | **0.00** | **/** |
| CuMo | **0.00** | **/** |
| CuRu | **-0.96** | 0.00 |
| CuHf | **0.00** | **/** |
| CuTa | **0.00** | **/** |
| CuW | **0.00** | **/** |
| CuRe | **0.00** | **/** |
| CuOs | **0.00** | **/** |

***Table S30***. The computed overpotential (*η*, V) of the FeM, CoM, NiM, and CuM dimer on M′M@NC.

|  | FeM | CoM | NiM | CuM |
| --- | --- | --- | --- | --- |
| Ti | 3.23 | 3.24 | 2.04 | 2.07 |
| V | 3.03 | 2.95 | 1.81 | 2.18 |
| Cr | 1.70 | 1.62 | 1.65 | 1.14 |
| Mn | 1.56 | 1.41 | 1.60 | 1.64 |
| Fe | 1.95 | 0.66 | 1.50 | 1.23 |
| Co | 0.66 | 0.73 | 0.71 | 0.34 |
| Ni | 1.50 | 0.71 | 0.39 | 0.25 |
| Cu | 1.23 | 0.31 | 0.25 | 0.25 |
| Zr | 2.61 | 2.13 | 1.94 | 2.09 |
| Nb | 2.97 | 1.98 | 2.17 | 3.32 |
| Mo | 2.40 | 2.51 | 2.48 | 2.62 |
| Ru | 1.68 | 1.26 | 1.16 | 0.86 |
| Rh | 0.64 | 0.65 | 0.63 | 0.40 |
| Pd | 1.23 | 0.22 | 0.06 | 0.07 |
| Ag | 0.95 | 0.45 | 0.58 | 0.41 |
| Hf | 3.06 | 2.37 | 2.02 | 1.88 |
| Ta | 2.50 | 3.44 | 2.79 | 3.64 |
| W | 2.67 | 2.79 | 3.22 | 3.25 |
| Re | 1.82 | 2.55 | 2.30 | 2.71 |
| Os | 1.99 | 1.88 | 1.53 | 1.42 |
| Ir | 0.73 | 0.77 | 0.82 | 0.55 |
| Pt | 1.13 | 0.55 | 0.14 | 0.08 |
| Au | 0.62 | 0.65 | 0.51 | 0.64 |

***Table S31***. Computed total energy (*E*_tot_), zero-potential correction energy (*E*_ZPE_) and entropy contribution (TS, T = 298.15 K) of the oxygenated intermediates on the CoCu@NC system.

| Species | *E*_cal_ (eV) | *E*_ZPE_ (eV) | TS (eV) |
| --- | --- | --- | --- |
| *OH | -644.04 | 0.37 | 0.07 |
| *OH-*OH | -653.82 | 0.71 | 0.15 |
| *OH-*O | -649.54 | 0.43 | 0.14 |
| *O_2_ | -644.28 | 0.13 | 0.16 |

***Table S32***. Computed total energy (*E*_tot_), zero-potential correction energy (*E*_ZPE_) and entropy contribution (TS, T = 298.15 K) of the oxygenated intermediates on the CoPd@NC system.

| Species | *E*_cal_ (eV) | *E*_ZPE_ (eV) | TS (eV) |
| --- | --- | --- | --- |
| *OH | -645.15 | 0.34 | 0.11 |
| *OH-*OH | -655.27 | 0.74 | 0.16 |
| *OH-*O | -650.60 | 0.43 | 0.15 |
| *O_2_ | -645.48 | 0.13 | 0.13 |

***Table S33***. Computed total energy (*E*_tot_), zero-potential correction energy (*E*_ZPE_) and entropy contribution (TS, T = 298.15 K) of the oxygenated intermediates on the NiCu@NC system.

| Species | *E*_cal_ (eV) | *E*_ZPE_ (eV) | TS (eV) |
| --- | --- | --- | --- |
| *OH | -642.36 | 0.37 | 0.07 |
| *OH-*OH | -652.65 | 0.74 | 0.16 |
| *OH-*O | -647.47 | 0.43 | 0.14 |
| *O_2_ | -642.49 | 0.14 | 0.13 |

***Table S34***. Computed total energy (*E*_tot_), zero-potential correction energy (*E*_ZPE_) and entropy contribution (TS, T = 298.15 K) of the oxygenated intermediates on the NiPd@NC system.

| Species | *E*_cal_ (eV) | *E*_ZPE_ (eV) | TS (eV) |
| --- | --- | --- | --- |
| *OH | -643.43 | 0.38 | 0.06 |
| *OH-*OH | -653.73 | 0.73 | 0.15 |
| OH-*O | -648.94 | 0.44 | 0.14 |
| *O_2_ | -644.00 | 0.14 | 0.12 |

***Table S35***. Computed total energy (*E*_tot_), zero-potential correction energy (*E*_ZPE_) and entropy contribution (TS, T = 298.15 K) of the oxygenated intermediates on the NiPt@NC system.

| Species | *E*_cal_ (eV) | *E*_ZPE_ (eV) | TS (eV) |
| --- | --- | --- | --- |
| *OH | -644.42 | 0.33 | 0.14 |
| *OH-*OH | -654.65 | 0.74 | 0.14 |
| *OH-*O | -650.01 | 0.44 | 0.13 |
| *O_2_ | -644.87 | 0.13 | 0.18 |

***Table S36***. Computed total energy (*E*_tot_), zero-potential correction energy (*E*_ZPE_) and entropy contribution (TS, T = 298.15 K) of the oxygenated intermediates on the CuCu@NC system.

| Species | *E*_cal_ (eV) | *E*_ZPE_ (eV) | TS (eV) |
| --- | --- | --- | --- |
| *OH | -640.36 | 0.37 | 0.07 |
| *OH-*OH | -650.04 | 0.71 | 0.18 |
| *OH-*O | -645.04 | 0.43 | 0.15 |
| *O_2_ | -640.36 | 0.14 | 0.14 |

***Table S37***. Computed total energy (*E*_tot_), zero-potential correction energy (*E*_ZPE_) and entropy contribution (TS, T = 298.15 K) of the oxygenated intermediates on the CuPd@NC system.

| Species | *E*_cal_ (eV) | *E*_ZPE_ (eV) | TS (eV) |
| --- | --- | --- | --- |
| *OH | -641.45 | 0.37 | 0.08 |
| *OH-*OH | -651.52 | 0.72 | 0.18 |
| *OH-*O | -646.63 | 0.43 | 0.13 |
| *O_2_ | -641.52 | 0.11 | 0.22 |

***Table S38***. Computed total energy (*E*_tot_), zero-potential correction energy (*E*_ZPE_) and entropy contribution (TS, T = 298.15 K) of the oxygenated intermediates on the CuPt@NC system.

| Species | *E*_cal_ (eV) | *E*_ZPE_ (eV) | TS (eV) |
| --- | --- | --- | --- |
| *OH | -642.46 | 0.37 | 0.08 |
| *OH-*OH | -652.45 | 0.73 | 0.17 |
| *OH-*O | -647.74 | 0.43 | 0.13 |
| *O_2_ | -642.64 | 0.12 | 0.21 |

***Table S39***. Computed total energy (*E*_tot_), zero-potential correction energy (*E*_ZPE_) and entropy contribution (TS, T = 298.15 K) of the oxygenated intermediates on the Co@NC system.

| Species | *E*_cal_ (eV) | *E*_ZPE_ (eV) | TS (eV) |
| --- | --- | --- | --- |
| *OH | -458.07 | 0.34 | 0.13 |
| *O | -452.79 | 0.06 | 0.07 |
| *OOH | -462.61 | 0.43 | 0.13 |
| *O_2_ | -458.54 | 0.14 | 0.15 |

***Table S40***. Computed total energy (*E*_tot_), zero-potential correction energy (*E*_ZPE_) and entropy contribution (TS, T = 298.15 K) of the oxygenated intermediates on the Cu@NC system.

| Species | *E*_cal_ (eV) | *E*_ZPE_ (eV) | TS (eV) |
| --- | --- | --- | --- |
| *OH | -453.23 | 0.31 | 0.10 |
| *O | -447.35 | 0.05 | 0.09 |
| *OOH | -457.71 | 0.40 | 0.19 |
| *O_2_ | -453.66 | 0.11 | 0.16 |

***Table S41***. Computed total energy (*E*_tot_), zero-potential correction energy (*E*_ZPE_) and entropy contribution (TS, T = 298.15 K) of the oxygenated intermediates on the Ni@NC system.

| Species | *E*_cal_ (eV) | *E*_ZPE_ (eV) | TS (eV) |
| --- | --- | --- | --- |
| *OH | -455.92 | 0.30 | 0.18 |
| *O | -450.04 | 0.01 | 0.09 |
| *OOH | -460.49 | 0.38 | 0.26 |
| *O_2_ | -456.50 | 0.10 | 0.21 |

***Table S42***. Computed total energy (*E*_tot_), zero-potential correction energy (*E*_ZPE_) and entropy contribution (TS, T = 298.15 K) of the oxygenated intermediates on the Pd@NC system.

| Species | *E*_cal_ (eV) | *E*_ZPE_ (eV) | TS (eV) |
| --- | --- | --- | --- |
| *OH | -454.65 | 0.30 | 0.12 |
| *O | -448.82 | 0.03 | 0.07 |
| *OOH | -459.32 | 0.40 | 0.21 |
| *O_2_ | -455.49 | 0.11 | 0.19 |

***Table S43***. Computed total energy (*E*_tot_), zero-potential correction energy (*E*_ZPE_) and entropy contribution (TS, T = 298.15 K) of the oxygenated intermediates on the Pt@NC system.

| Species | *E*_cal_ (eV) | *E*_ZPE_ (eV) | TS (eV) |
| --- | --- | --- | --- |
| *OH | -455.78 | 0.30 | 0.13 |
| *O | -450.278 | 0.04 | 0.09 |
| *OOH | -460.48 | 0.39 | 0.24 |
| *O_2_ | -456.69 | 0.11 | 0.18 |

***Table S44***. Computed total energy (*E*_tot_), zero-potential correction energy (*E*_ZPE_) and entropy contribution (TS, T = 298.15 K) of the oxygenated intermediates on the CoCu@NC system along the AEM.

| Species | *E*_cal_ (eV) | *E*_ZPE_ (eV) | TS (eV) |
| --- | --- | --- | --- |
| *O | -639.58 | 0.08 | 0.04 |
| *OOH | -648.46 | 0.42 | 0.19 |
| *O_2_ | -644.28 | 0.13 | 0.16 |

***Table S45***. Computed total energy (*E*_tot_), zero-potential correction energy (*E*_ZPE_) and entropy contribution (TS, T = 298.15 K) of the oxygenated intermediates on the CoPd@NC system along the AEM.

| Species | *E*_cal_ (eV) | *E*_ZPE_ (eV) | TS (eV) |
| --- | --- | --- | --- |
| *O | -640.20 | 0.08 | 0.04 |
| *OOH | -649.08 | 0.43 | 0.17 |
| *O_2_ | -645.52 | 0.14 | 0.16 |

***Table S46***. Computed total energy (*E*_tot_), zero-potential correction energy (*E*_ZPE_) and entropy contribution (TS, T = 298.15 K) of the oxygenated intermediates on the NiCu@NC system along the AEM.

| Species | *E*_cal_ (eV) | *E*_ZPE_ (eV) | TS (eV) |
| --- | --- | --- | --- |
| *O | -637.72 | 0.08 | 0.04 |
| *OOH | -646.12 | 0.44 | 0.17 |
| *O_2_ | -642.53 | 0.12 | 0.12 |

***Table S47***. Computed total energy (*E*_tot_), zero-potential correction energy (*E*_ZPE_) and entropy contribution (TS, T = 298.15 K) of the oxygenated intermediates on the NiPd@NC system along the AEM.

| Species | *E*_cal_ (eV) | *E*_ZPE_ (eV) | TS (eV) |
| --- | --- | --- | --- |
| *O | -638.49 | 0.09 | 0.03 |
| *OOH | -647.35 | 0.44 | 0.16 |
| *O_2_ | -643.05 | 0.15 | 0.13 |

***Table S48***. Computed total energy (*E*_tot_), zero-potential correction energy (*E*_ZPE_) and entropy contribution (TS, T = 298.15 K) of the oxygenated intermediates on the NiPt@NC system along the AEM.

| Species | *E*_cal_ (eV) | *E*_ZPE_ (eV) | TS (eV) |
| --- | --- | --- | --- |
| *O | -639.59 | 0.09 | 0.03 |
| *OOH | -648.32 | 0.44 | 0.17 |
| *O_2_ | -644.86 | 0.13 | 0.19 |

***Table S49***. Computed total energy (*E*_tot_), zero-potential correction energy (*E*_ZPE_) and entropy contribution (TS, T = 298.15 K) of the oxygenated intermediates on the CuCu@NC system along the AEM.

| Species | *E*_cal_ (eV) | *E*_ZPE_ (eV) | TS (eV) |
| --- | --- | --- | --- |
| *O | -634.69 | 0.08 | 0.04 |
| *OOH | -643.93 | 0.43 | 0.19 |
| *O_2_ | -640.31 | 0.13 | 0.15 |

***Table S50***. Computed total energy (*E*_tot_), zero-potential correction energy (*E*_ZPE_) and entropy contribution (TS, T = 298.15 K) of the oxygenated intermediates on the CuPd@NC system along the AEM.

| Species | *E*_cal_ (eV) | *E*_ZPE_ (eV) | TS (eV) |
| --- | --- | --- | --- |
| *O | -636.07 | 0.08 | 0.04 |
| *OOH | -645.24 | 0.44 | 0.17 |
| *O_2_ | -641.49 | 0.11 | 0.26 |

***Table S51***. Computed total energy (*E*_tot_), zero-potential correction energy (*E*_ZPE_) and entropy contribution (TS, T = 298.15 K) of the oxygenated intermediates on the CuPt@NC system along the AEM.

| Species | *E*_cal_ (eV) | *E*_ZPE_ (eV) | TS (eV) |
| --- | --- | --- | --- |
| *O | -637.14 | 0.08 | 0.04 |
| *OOH | -646.22 | 0.44 | 0.18 |
| *O_2_ | -641.49 | 0.11 | 0.26 |

***Table S52.*** Δ*G*_*OH→*OH-*OH_ of the second *OH adsorbed at different sites (M-M site, M-C site and M-M(anti) site) on the FeM@NC DACs.

| FeM | Δ*G*_*OH-*OH_ -Δ*G*_*OH_ | | |
| --- | --- | --- | --- |
|  | M-M site | M-C site | M-M(anti) site |
| FeTi | -0.64 | 1.07 | -0.61 |
| FeV | -0.57 | 1.33 | -0.52 |
| FeCr | -0.07 | 1.64 | -0.33 |
| FeMn | 0.19 | 1.25 | -0.02 |
| FeFe | 0.18 | 1.53 | 0.29 |
| FeCo | 0.86 | 2.09 | 1.41 |
| FeNi | 0.77 | 1.43 | 0.34 |
| FeCu | 0.60 | 1.55 | 0.22 |
| FeZr | -0.68 | 1.60 | 0.07 |
| FeNb | -1.05 | 0.71 | -1.04 |
| FeMo | -1.06 | 0.56 | -0.93 |
| FeRu | 0.44 | 1.27 | 0.17 |
| FeRh | 0.61 | 1.43 | 0.82 |
| FePd | 0.91 | 1.57 | 1.10 |
| FeAg | 0.81 | 1.92 | 1.03 |
| FeHf | -0.83 | 1.25 | -0.62 |
| FeTa | -1.23 | 0.54 | -1.11 |
| FeW | -1.47 | 0.43 | -1.41 |
| FeRe | 0.39 | 1.32 | -0.19 |
| FeOs | 0.39 | 1.17 | -0.17 |
| FeIr | 0.87 | 1.49 | 0.58 |
| FePt | 1.14 | 1.76 | 1.16 |
| FeAu | 1.13 | 1.41 | 0.82 |

***Table S53.*** Δ*G*_*OH→*OH-*OH_ of the second *OH adsorbed at different sites (M-M site, M-C site and M-M(anti) site) on the CoM@NC DACs.

| CoM | Δ*G*_*OH-*OH_ -Δ*G*_*OH_ | | |
| --- | --- | --- | --- |
|  | M-M site | M-C site | M-M(anti) site |
| CoTi | -0.38 | 1.09 | -0.79 |
| CoV | -0.18 | 1.26 | 0.80 |
| CoCr | 0.23 | 2.13 | 0.62 |
| CoMn | 0.19 | 1.26 | 0.78 |
| CoCo | 1.05 | 3.37 | 3.22 |
| CoNi | 0.88 | 1.83 | 0.91 |
| CoCu | 1.41 | 1.49 | 0.37 |
| CoZr | -0.47 | 1.62 | 0.86 |
| CoNb | -0.22 | 1.10 | 1.02 |
| CoMo | -0.02 | 1.38 | -0.13 |
| CoRu | 0.27 | 1.80 | -0.03 |
| CoRh | 1.12 | 1.45 | 0.56 |
| CoPd | 1.16 | 1.36 | 1.41 |
| CoAg | 0.72 | 1.65 | 0.33 |
| CoHf | -0.65 | 1.27 | 0.47 |
| CoTa | -0.32 | 1.09 | 0.68 |
| CoW | 0.07 | 1.24 | -0.14 |
| CoRe | 0.01 | 1.33 | -0.25 |
| CoOs | 0.22 | 1.55 | 0.13 |
| CoIr | 1.11 | 2.52 | 0.68 |
| CoPt | 1.30 | 1.93 | 1.61 |
| CoAu | 1.88 | 1.57 | 0.63 |

***Table S54.*** Δ*G*_*OH→*OH-*OH_ of the second *OH adsorbed at different sites (M-M site, M-C site and M-M(anti) site) on the NiM@NC DACs.

| NiM | Δ*G*_*OH-*OH_ -Δ*G*_*OH_ | | |
| --- | --- | --- | --- |
|  | M-M site | M-C site | M-M(anti) site |
| NiTi | 0.03 | 1.28 | 0.01 |
| NiV | 0.17 | 1.35 | 0.10 |
| NiCr | 0.23 | 1.34 | -0.03 |
| NiMn | 0.48 | 1.34 | 0.28 |
| NiNi | 1.28 | 1.18 | 1.19 |
| NiCu | 0.92 | 1.50 | 1.22 |
| NiZr | -0.54 | 1.40 | -0.08 |
| NiNb | -0.74 | 1.16 | 0.00 |
| NiMo | -0.04 | 1.41 | 0.22 |
| NiRu | 0.29 | 1.61 | 0.24 |
| NiRh | 1.04 | 1.56 | 0.43 |
| NiPd | 0.88 | 1.27 | 0.88 |
| NiAg | 0.80 | 1.87 | 0.82 |
| NiHf | -0.59 | 1.21 | -0.56 |
| NiTa | -0.92 | 1.50 | -0.22 |
| NiW | -0.27 | 1.36 | -0.11 |
| NiRe | -0.19 | 1.37 | 0.05 |
| NiOs | -0.06 | 1.53 | 0.11 |
| NiIr | 0.85 | 1.46 | 0.33 |
| NiPt | 1.11 | 1.38 | 1.13 |
| NiAu | 1.00 | 1.51 | 1.34 |

***Table S55.*** Δ*G*_*OH→*OH-*OH_ of the second *OH adsorbed at different sites (M-M site, M-C site and M-M(anti) site) on the CuM@NC DACs.

| CuM | Δ*G*_*OH-*OH_ -Δ*G*_*OH_ | | |
| --- | --- | --- | --- |
|  | M-M site | M-C site | M-M(anti) site |
| CuTi | -0.93 | 0.70 | -0.78 |
| CuV | 0.05 | 1.46 | 0.11 |
| CuCr | 0.33 | 1.49 | 0.10 |
| CuMn | 0.38 | 1.28 | 0.33 |
| CuCu | 1.48 | 1.66 | 1.29 |
| CuZr | -0.53 | 1.70 | -0.04 |
| CuNb | -0.63 | 1.35 | -0.20 |
| CuMo | -0.63 | 1.24 | -0.28 |
| CuRu | 0.25 | 1.54 | -0.22 |
| CuRh | 1.05 | 1.72 | 0.11 |
| CuPd | 1.10 | 1.51 | 1.20 |
| CuAg | 1.28 | 1.95 | 1.70 |
| CuHf | -0.72 | 1.46 | -0.48 |
| CuTa | -0.92 | 1.26 | -0.74 |
| CuW | -0.88 | 1.22 | -0.49 |
| CuRe | -0.49 | 1.42 | -0.32 |
| CuOs | -0.02 | 1.44 | -0.32 |
| CuIr | 0.73 | 1.62 | -0.05 |
| CuPt | 1.21 | 1.37 | 1.19 |
| CuAu | 1.53 | 1.77 | 1.34 |

***Table S56.*** Desorption free energy change (Δ*G*_*O2→O2(g)_) for *O_2_ toward O_2_ of FeM, CoM, NiM and CuM. Bolded font: With a benchmark of 1.17 eV for NiFe-CNG.

|  | FeM | CoM | NiM | CuM |
| --- | --- | --- | --- | --- |
| Ti | 2.07 | 1.74 | 2.54 | 2.70 |
| V | 2.29 | 1.99 | 3.04 | 2.36 |
| Cr | 2.93 | 2.46 | 1.95 | 2.12 |
| Mn | 2.48 | 2.04 | 1.29 | 1.34 |
| Fe | 2.09 | 1.46 | 1.23 | 1.36 |
| Co | 1.46 | 1.49 | **1.09** | **0.88** |
| Ni | 1.23 | **1.09** | **0.27** | **0.28** |
| Cu | 1.36 | **0.88** | **0.28** | **0.18** |
| Zr | 2.79 | 2.86 | 2.52 | 2.52 |
| Nb | 2.54 | 3.08 | 3.40 | 2.37 |
| Mo | 2.82 | 2.44 | 3.71 | 2.81 |
| Ru | 1.70 | 1.95 | 1.69 | 1.68 |
| Rh | 1.44 | 1.39 | **0.77** | **0.45** |
| Pd | 1.82 | **0.96** | **0.39** | **0.10** |
| Ag | 1.73 | **0.84** | **0.70** | **0.20** |
| Hf | 2.62 | 2.88 | 2.66 | 2.94 |
| Ta | 3.73 | 2.01 | 4.02 | 2.71 |
| W | 3.90 | 4.02 | 4.45 | 3.27 |
| Re | 2.68 | 2.07 | 2.49 | 3.94 |
| Os | 1.72 | 1.92 | 1.87 | 1.78 |
| Ir | 1.45 | 1.49 | **0.78** | **0.45** |
| Pt | 1.80 | **0.88** | **0.26** | **0.15** |
| Au | 1.46 | **0.81** | **0.17** | **-0.11** |

***Table S57.*** Adsorption free energy of FeM@Gr for *OH and the calculated relative energy of the optimized adsorption configuration for *OH-*OH. Bold fonts represent the most stable configuration.

| **FeM** | **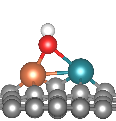** | **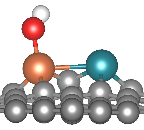** | **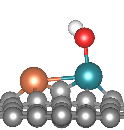** | **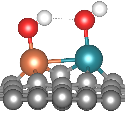** | **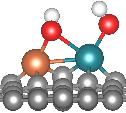** | **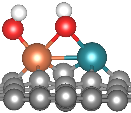** |
| --- | --- | --- | --- | --- | --- | --- |
| FeTi | **-1.92** | / | / | / | **0.00** | / |
| FeV | **-2.03** | / | -1.43 | / | **0.00** | 0.63 |
| FeCr | **-2.00** | / | -1.40 | **0.00** | 0.09 | / |
| FeMn | **-1.95** | / | / | **0.00** | 0.20 | / |
| Fe_2_ | **-1.55** | -1.12 | / | **0.00** | / | / |
| FeCo | **-1.22** | -1.16 | / | **0.00** | / | / |
| FeNi | **-1.06** | -1.11 | / | **0.00** | 0.20 | / |
| FeCu | **-0.85** | / | -0.74 | 0.07 | / | **0.00** |
| FeZr | **-1.89** | / | / | 0.90 | **0.00** |  |
| FeNb | **-1.87** | / | -1.37 | 0.75 | **0.00** |  |
| FeMo | **-1.59** | / | -1.37 |  | **0.00** | 0.95 |
| FeRu | **-1.32** | -1.08 | -0.95 | **0.00** | / | 0.52 |
| FeRh | -1.12 | **-1.20** | -0.48 | **0.00** | / | 0.41 |
| FePd | -1.03 | **-1.15** | / | **0.00** | / | 0.29 |
| FeAg | -2.09 | **-2.17** | / | **0.00** | / | 0.14 |
| FeHf | **-1.99** | / | -1.52 | / | **0.00** | 1.15 |
| FeTa | **-2.04** | / | -1.72 | / | **0.00** | / |
| FeW | -1.80 | / | **-1.88** | 0.27 | **0.00** | / |
| FeRe | -1.48 | / | **-1.77** | 0.18 | **0.00** | / |
| FeOs | -1.49 | -1.07 | **-1.55** | 0.02 | **0.00** | / |
| FeIr | **-1.21** | -1.15 | -0.96 | **0.00** | 0.23 | / |
| FePt | -1.05 | **-1.12** | -0.43 | **0.00** | / | / |
| FeAu | **-0.87** | -0.81 | 0.01 | / | 0.29 | **0.00** |

***Table S58.*** Adsorption free energy of CoM@Gr for *OH and the calculated relative energy of the optimized adsorption configuration for *OH-*OH. Bold fonts represent the most stable configuration.

| **CoM** | 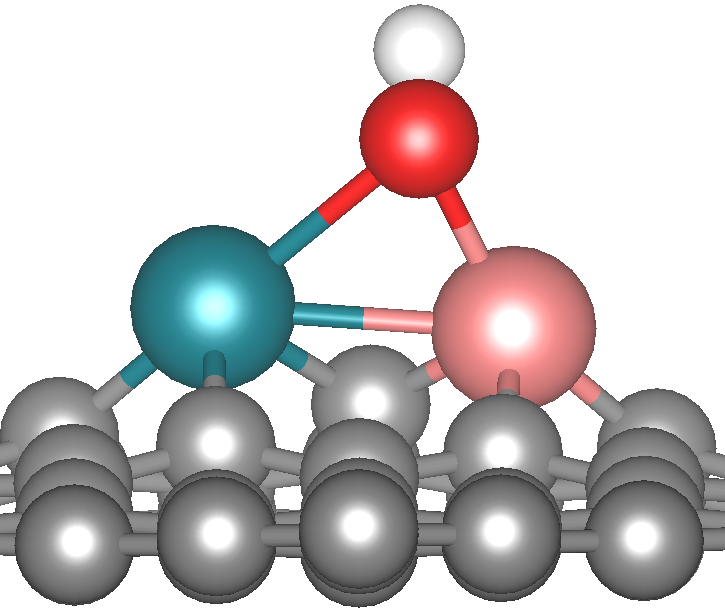 | 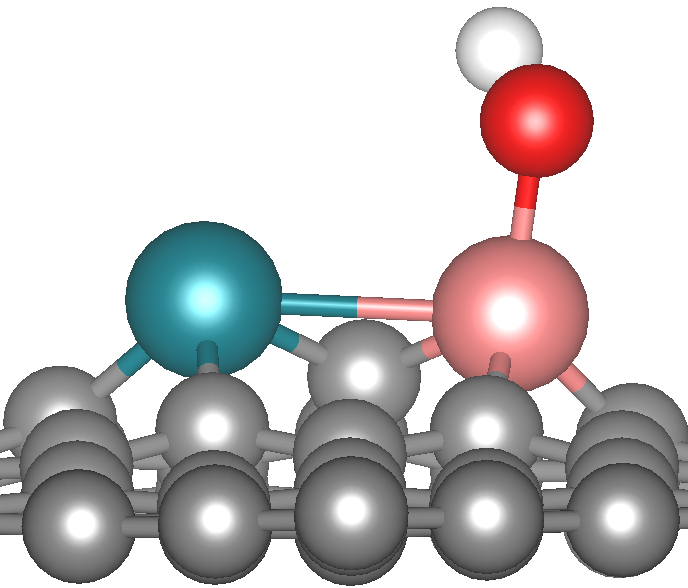 | 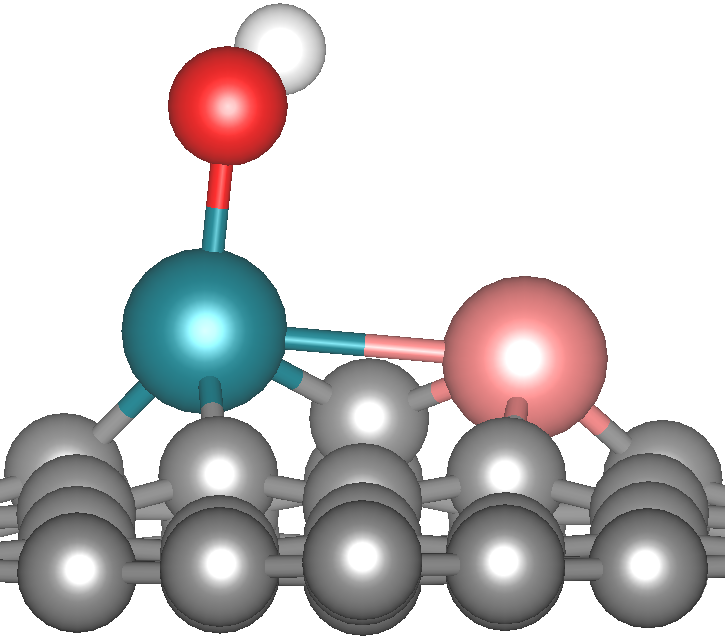 | 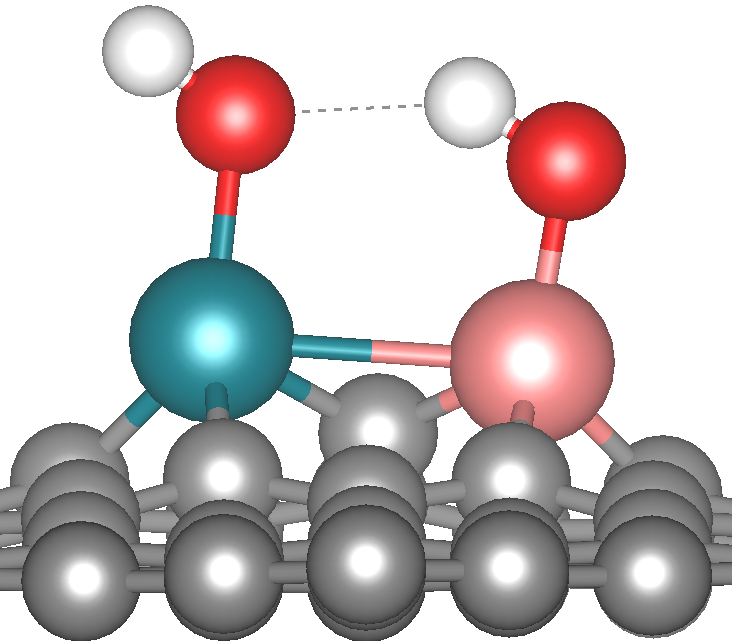 | 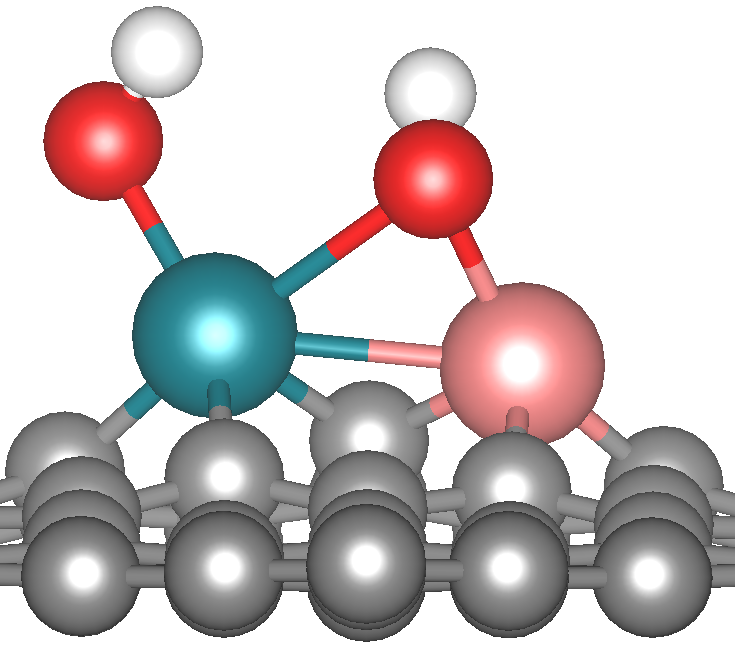 | 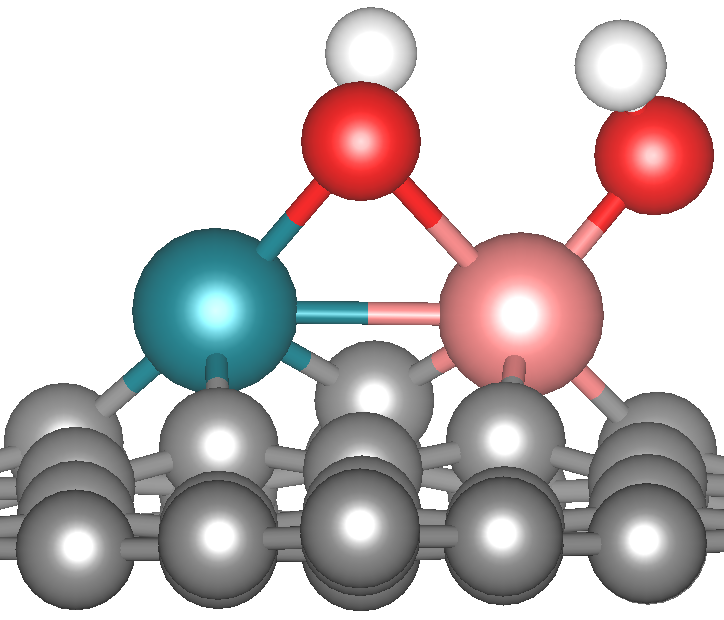 |
| --- | --- | --- | --- | --- | --- | --- |
| CoTi | **-1.81** | / | -1.26 | / | **0.00** | / |
| CoV | **-1.71** | / | -1.38 | / | **0.00** | 0.86 |
| CoCr | **-1.48** | / | -1.31 | 0.15 | **0.00** | / |
| CoMn | **-1.56** | / | -1.33 | **0.00** | 0.07 | / |
| Co_2_ | **-1.20** | / | / | **0.00** | / | / |
| CoNi | **-0.94** | -0.64 | / | / | 0.33 | **0.00** |
| CoCu | **-0.46** | -0.46 | / | / | 0.35 | **0.00** |
| CoZr | **-1.76** | / | -1.16 | 0.87 | **0.00** | / |
| CoNb | **-1.56** | / | -1.38 | 0.98 | **0.00** | / |
| CoMo | **-1.22** | / | -1.28 | / | **0.00** | / |
| CoRu | **-1.03** | / | -1.00 | **0.00** | / | / |
| CoRh | **-1.00** | / | -0.56 | **0.00** | / | / |
| CoPd | **-0.84** | / | / | 0.11 | / | **0.00** |
| CoAg | **-0.61** | -0.54 | / | / | 0.24 | **0.00** |
| CoHf | **-1.93** | / | -1.47 | 1.41 | **0.00** | / |
| CoTa | **-1.76** | / | -1.76 | / | **0.00** | / |
| CoW | -1.46 | / | **-1.80** | / | **0.00** | / |
| CoRe | -1.16 | / | **-1.62** | / | **0.00** | / |
| CoOs | -1.14 | / | **-1.55** | **0.00** | 0.01 | / |
| CoIr | **-1.11** | -0.84 | -1.05 | **0.00** | / | / |
| CoPt | **-0.94** | -0.67 | / | / | **0.00** | / |
| CoAu | **-0.56** | -0.39 | / | / | 0.05 | **0.00** |

***Table S59.*** Adsorption free energy of NiM@Gr for *OH and the calculated relative energy of the optimized adsorption configuration for *OH-*OH. Bold fonts represent the most stable configuration.

| **NiM** | 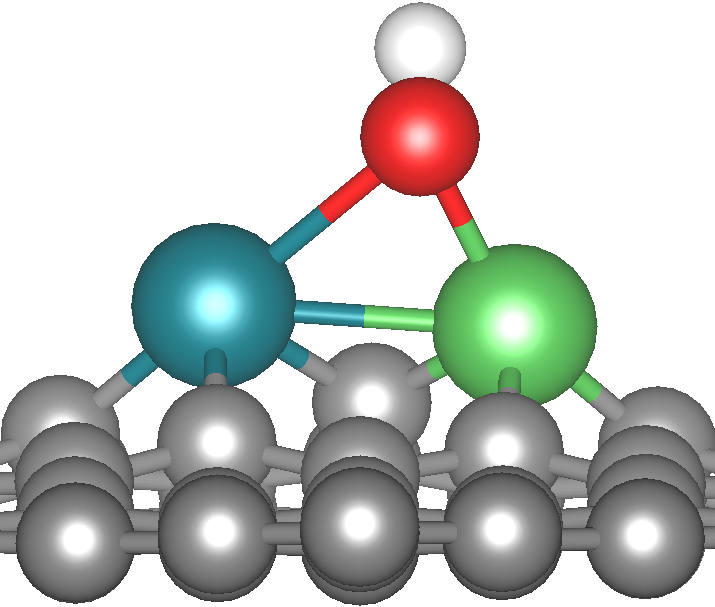 | 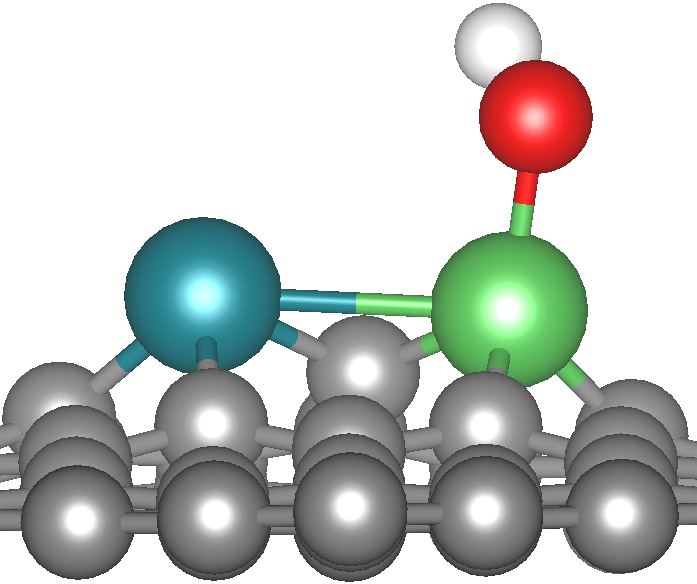 | 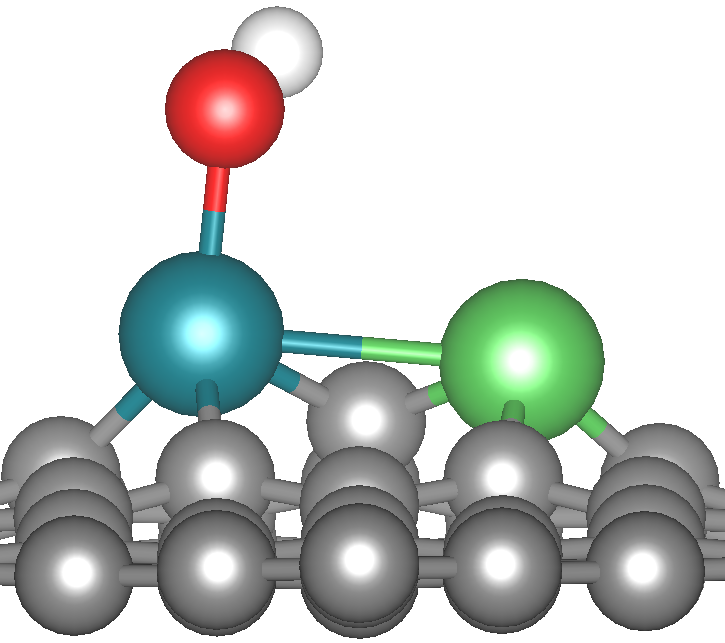 | 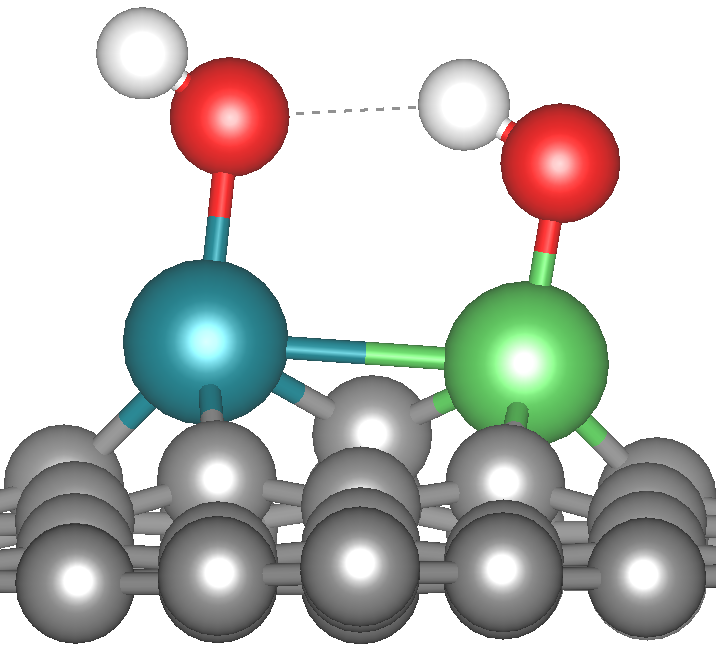 | 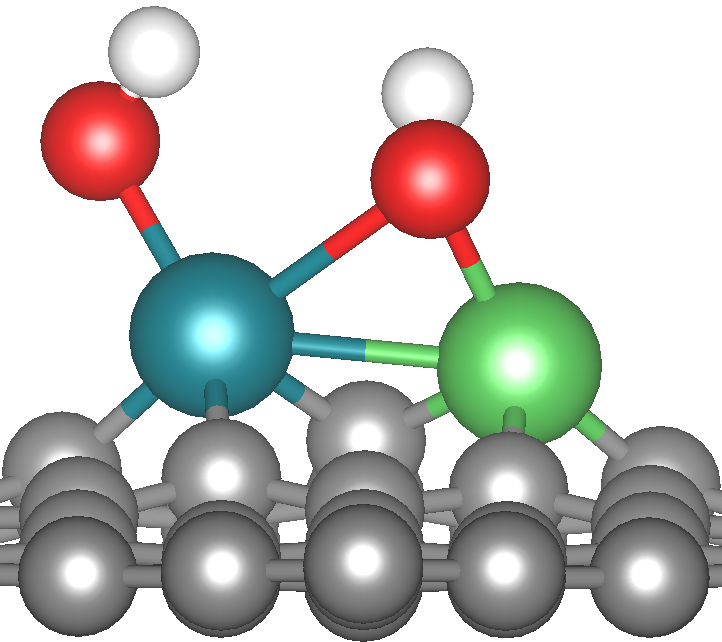 | 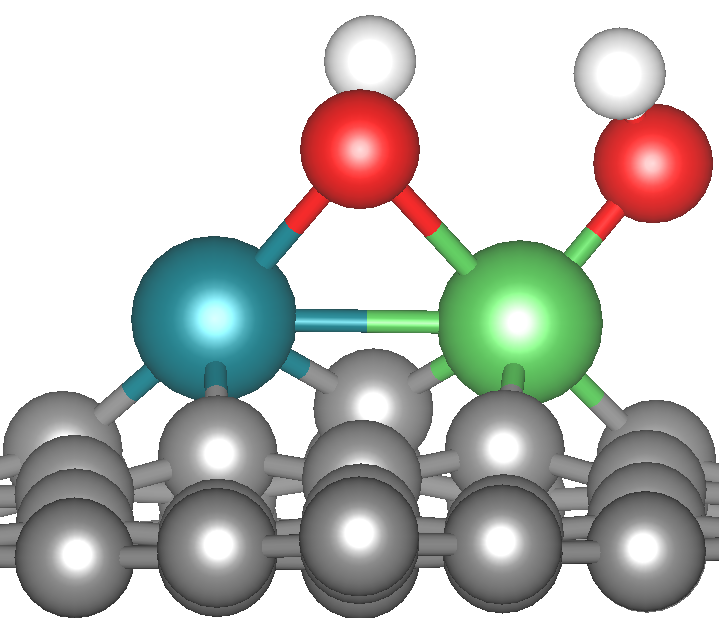 |
| --- | --- | --- | --- | --- | --- | --- |
| NiTi | **-1.54** | / | -1.17 | / | **0.00** | / |
| NiV | -1.24 | / | **-1.27** | / | **0.00** | / |
| NiCr | -1.08 | / | **-1.27** | 0.18 | **0.00** | / |
| NiMn | -0.92 | / | **-1.31** | 0.06 | **0.00** | / |
| Ni_2_ | **-0.63** | / | -0.03 | / | **0.00** | / |
| NiCu | **-0.50** | / | / | / | / | **0.00** |
| NiZr | **-1.54** | / | -1.08 | 0.93 | **0.00** | / |
| NiNb | -1.14 | / | **-1.29** | / | **0.00** | / |
| NiMo | -0.57 | / | **-1.08** | / | **0.00** | / |
| NiRu | **-0.80** | / | / | **0.00** | / | / |
| NiRh | **-0.71** | / | -0.35 | **0.00** | / | 0.46 |
| NiPd | **-0.55** | / | 0.06 | / | **0.00** | 0.25 |
| NiAg | **-0.42** | / | / | / | **0.00** | 0.95 |
| NiHf | **-1.68** | / | -1.44 | / | **0.00** | / |
| NiTa | -1.34 | / | **-1.69** | / | **0.00** | / |
| NiW | / | / | **-1.60** | / | **0.00** | / |
| NiRe | / | / | **-1.87** | 0.36 | **0.00** | / |
| NiOs | -0.94 | / | **-1.50** | 0.03 | **0.00** | / |
| NiIr | **-0.87** | / | -0.86 | / | **0.00** | / |
| NiPt | **-0.66** | / | -0.31 | / | **0.00** | 0.60 |
| NiAu | **-0.61** | / | / | / | **0.00** | 0.25 |

***Table S60.*** Adsorption free energy of CuM@Gr for *OH and the calculated relative energy of the optimized adsorption configuration for *OH-*OH. Bold fonts represent the most stable configuration.

| **CuM** | 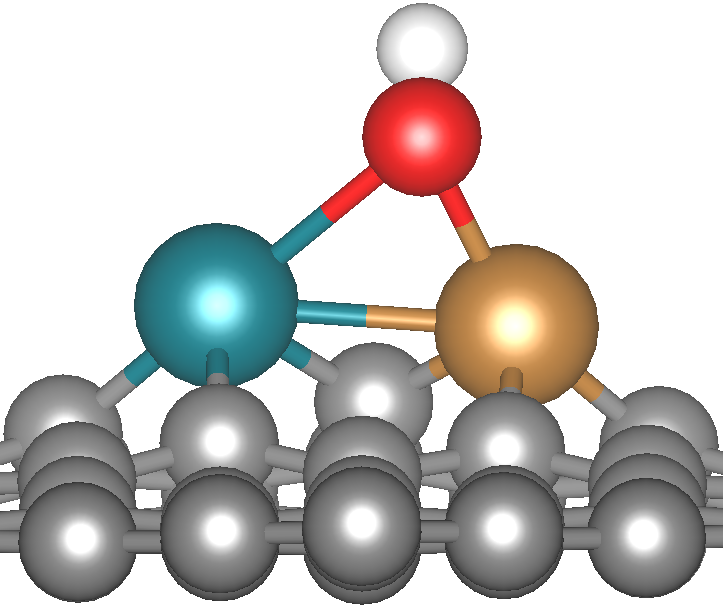 | 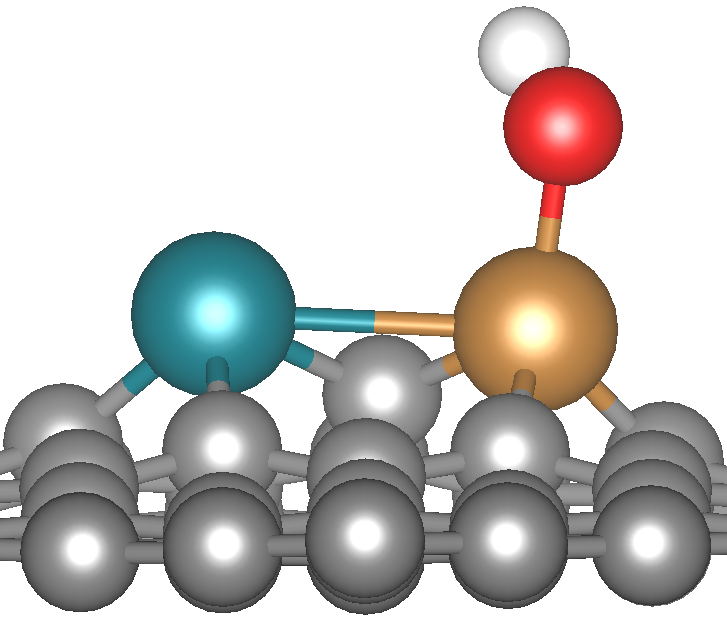 | 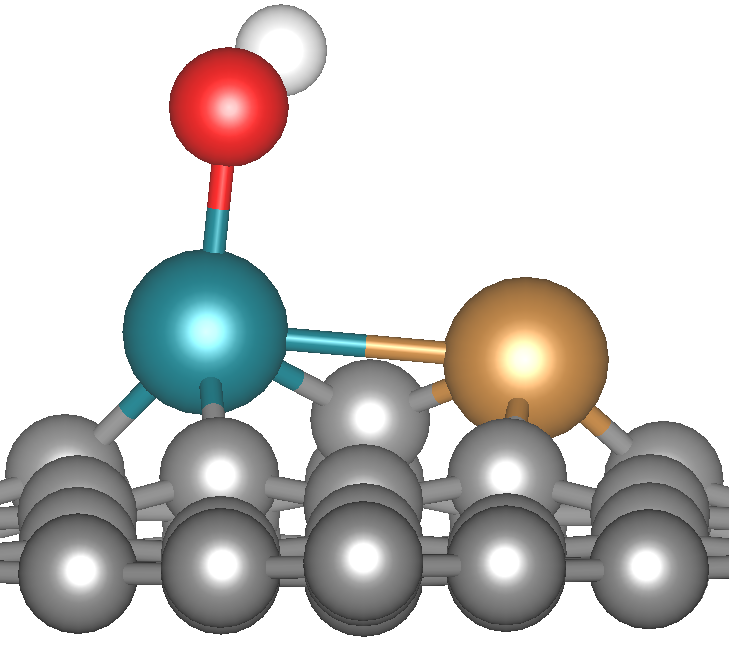 | 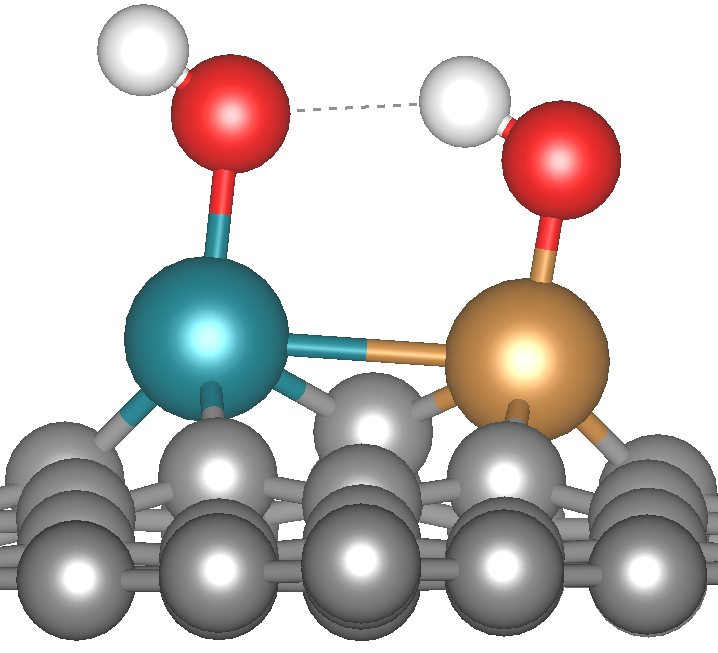 | 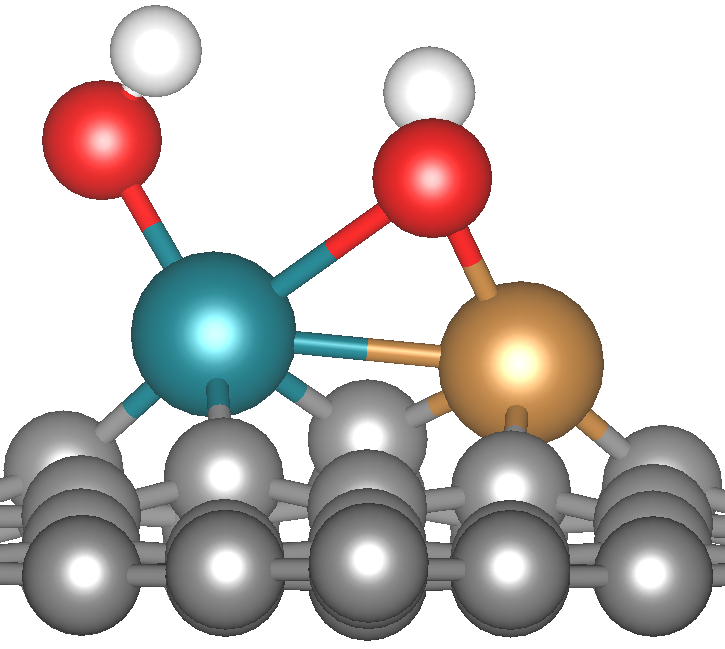 | 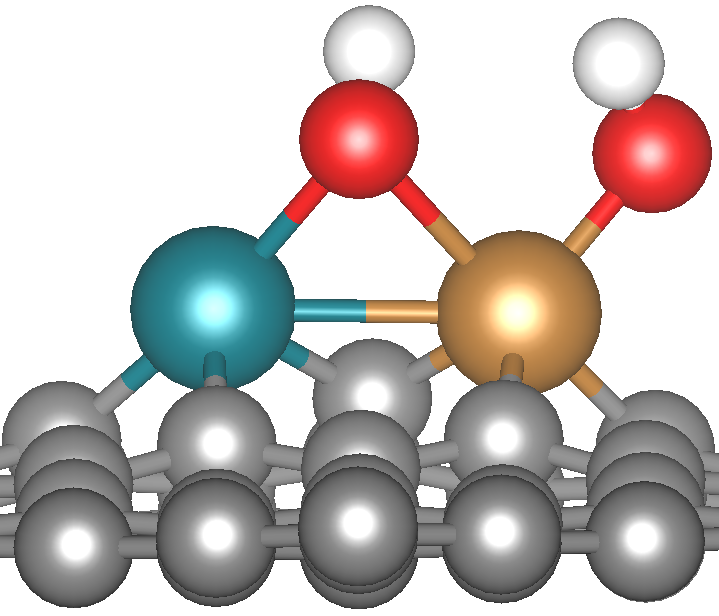 |
| --- | --- | --- | --- | --- | --- | --- |
| CuTi | / | / | **-1.56** |  | **0.00** |  |
| CuV | / | / | **-1.18** |  | **0.00** |  |
| CuCr | -0.74 | / | **-1.41** | 0.28 | **0.00** |  |
| CuMn | -0.78 | / | **-1.19** | 0.10 | **0.00** |  |
| Cu2 | **-0.15** | / | / |  | **0.00** |  |
| CuZr | / | / | **-1.21** |  | **0.00** |  |
| CuNb | / | / | **-1.14** |  | **0.00** |  |
| CuMo | / | / | **-1.32** |  | **0.00** |  |
| CuRu | -0.39 | / | **-0.76** | **0.00** |  |  |
| CuRh | -0.23 | / | **-0.28** |  | **0.00** |  |
| CuPd | **-0.30** | / | / |  | **0.00** | 0.75 |
| CuAg | **-0.20** | / | / |  | **0.00** | 1.51 |
| CuHf | / | / | **-1.56** |  | **0.00** |  |
| CuTa | / | / | **-1.57** |  | **0.00** |  |
| CuW | / | / | **-1.85** |  | **0.00** |  |
| CuRe | / | / | **-1.66** |  | **0.00** |  |
| CuOs | -0.49 | / | **-1.31** | 0.40 | **0.00** |  |
| CuIr | -0.34 | / | **-0.70** |  | **0.00** |  |
| CuPt | **-0.50** | / | -0.20 |  | **0.00** |  |
| CuAu | **-0.32** | / | / |  | **0.00** | 0.27 |

***Table S61***. Computed energy of total energy of M_2_@NC, where *E*_NC_ = 620.53 eV. For comparison, the BE, *E*_agg_, the number of transferred electrons (*N*_e_) during the dissolution and dissolution potential (*U*_diss_) are also listed.

| M_2_ | *E*_M2@NC_ | *N*_e_ | *U*_diss_ | BE | *E*_agg_ |
| --- | --- | --- | --- | --- | --- |
| Ti | -641.80 | 2 | 1.17 | -8.11 | -2.80 |
| V | -641.29 | 2 | 0.21 | -6.74 | -1.38 |
| Cr | -640.51 | 2 | -0.43 | -4.55 | -0.48 |
| Mn | -640.41 | 2 | -0.24 | -4.79 | -0.95 |
| Zr | -643.88 | 4 | 0.13 | -9.31 | -3.15 |
| Nb | -643.12 | 3 | -0.38 | -8.08 | -1.08 |
| Mo | -641.52 | 3 | -0.49 | -5.85 | 0.44 |
| Ru | -638.66 | 2 | 0.29 | -6.59 | 0.17 |
| Rh | -636.15 | 2 | 1.16 | -6.54 | -0.56 |
| Pd | -632.53 | 2 | 1.73 | -4.53 | -0.78 |
| Ag | -625.99 | 1 | 0.81 | -2.53 | -0.01 |
| Hf | -646.40 | 4 | -0.04 | -9.43 | -3.01 |
| Ta | -644.96 | 3 | -0.33 | -8.51 | -0.40 |
| W | -644.25 | 3 | -0.63 | -7.29 | 1.09 |
| Re | -644.11 | 3 | -0.13 | -7.18 | 0.64 |
| Os | -641.67 | 8 | 0.67 | -7.65 | 0.68 |
| Ir | -638.687 | 3 | 1.31 | -7.52 | -0.23 |
| Pt | -634.623 | 2 | 2.13 | -6.51 | -0.95 |
| Au | -626.739 | 3 | 1.42 | -2.92 | 0.11 |

***Table S62***. Computed total energy (*E*_tot_), zero-potential correction energy (*E*_ZPE_) and entropy contribution (TS, T = 298.15 K) of the oxygenated intermediates on the Ni_2_@NC system.

| Species | *E*_cal_ (eV) | *E*_ZPE_ (eV) | TS (eV) |
| --- | --- | --- | --- |
| *OH | -644.43 | 0.40 | 0.05 |
| *OH-*OH | -654.34 | 0.75 | 0.14 |
| *OH-*O | -650.00 | 0.43 | 0.13 |
| *O_2_ | -644.68 | 0.13 | 0.13 |

***Table S63***. Computed total energy (*E*_tot_), zero-potential correction energy (*E*_ZPE_) and entropy contribution (TS, T = 298.15 K) of the oxygenated intermediates on the Cu_2_@NC system.

| Species | *E*_cal_ (eV) | *E*_ZPE_ (eV) | TS (eV) |
| --- | --- | --- | --- |
| *OH | -640.36 | 0.37 | 0.07 |
| *OH-*OH | -650.04 | 0.71 | 0.18 |
| *OH-*O | -645.04 | 0.43 | 0.15 |
| *O_2_ | -640.36 | 0.14 | 0.14 |

***Table S64***. Computed total energy (*E*_tot_), zero-potential correction energy (*E*_ZPE_) and entropy contribution (TS, T = 298.15 K) of the oxygenated intermediates on the Rh_2_@NC system.

| Species | *E*_cal_ (eV) | *E*_ZPE_ (eV) | TS (eV) |
| --- | --- | --- | --- |
| *OH | -646.89 | 0.39 | 0.06 |
| *OH-*OH | -656.90 | 0.69 | 0.14 |
| *OH-*O | -652.72 | 0.43 | 0.14 |
| *O_2_ | -646.73 | 0.14 | 0.12 |

***Table S65***. Computed total energy (*E*_tot_), zero-potential correction energy (*E*_ZPE_) and entropy contribution (TS, T = 298.15 K) of the oxygenated intermediates on the Pd_2_@NC system.

| Species | *E*_cal_ (eV) | *E*_ZPE_ (eV) | TS (eV) |
| --- | --- | --- | --- |
| *OH | -642.30 | 0.38 | 0.06 |
| *OH-*OH | -652.66 | 0.70 | 0.20 |
| *OH-*O | -647.59 | 0.42 | 0.15 |
| *O_2_ | -642.99 | 0.14 | 0.13 |

***Table S66***. Computed total energy (*E*_tot_), zero-potential correction energy (*E*_ZPE_) and entropy contribution (TS, T = 298.15 K) of the oxygenated intermediates on the Ag_2_@NC system.

| Species | *E*_cal_ (eV) | *E*_ZPE_ (eV) | TS (eV) |
| --- | --- | --- | --- |
| *OH | -636.48 | 0.35 | 0.11 |
| *OH-*OH | -645.95 | 0.66 | 0.21 |
| *OH-*O | -640.62 | 0.40 | 0.18 |
| *O_2_ | -636.50 | 0.13 | 0.16 |

***Table S67***. Computed total energy (*E*_tot_), zero-potential correction energy (*E*_ZPE_) and entropy contribution (TS, T = 298.15 K) of the oxygenated intermediates on the Ir_2_@NC system.

| Species | *E*_cal_ (eV) | *E*_ZPE_ (eV) | TS (eV) |
| --- | --- | --- | --- |
| *OH | -649.15 | 0.40 | 0.06 |
| *OH-*OH | -659.08 | 0.74 | 0.16 |
| *OH-*O | -655.32 | 0.44 | 0.14 |
| *O_2_ | -649.33 | 0.13 | 0.17 |

***Table S68***. Computed total energy (*E*_tot_), zero-potential correction energy (*E*_ZPE_) and entropy contribution (TS, T = 298.15 K) of the oxygenated intermediates on the Pt_2_@NC system.

| Species | *E*_cal_ (eV) | *E*_ZPE_ (eV) | TS (eV) |
| --- | --- | --- | --- |
| *OH | -644.26 | 0.35 | 0.10 |
| *OH-*OH | -654.77 | 0.71 | 0.20 |
| *OH-*O | -649.61 | 0.43 | 0.14 |
| *O_2_ | -644.71 | 0.14 | 0.12 |

***Table S69***. Computed total energy (*E*_tot_), zero-potential correction energy (*E*_ZPE_) and entropy contribution (TS, T = 298.15 K) of the oxygenated intermediates on the Au_2_@NC system.

| Species | *E*_cal_ (eV) | *E*_ZPE_ (eV) | TS (eV) |
| --- | --- | --- | --- |
| *OH | -636.28 | 0.30 | 0.13 |
| *OH-*OH | -646.86 | 0.68 | 0.28 |
| *OH-*O | -642.06 | 0.44 | 0.14 |
| *O_2_ | -636.84 | 0.11 | 0.17 |

***Table S70.*** Gibbs free energy change (Δ*G*) for *OH toward *OH-*OH versus *OH → *O + H^+^ +e^−^ of FeM. Yellow highlights represent candidate DACs that possessed *O selectivity (pink part in Figure 6a) and potentially followed the hybrid pathway, as shown in Figure S16.

| FeM | Δ*G*(*OH→*OHOH) | Δ*G*(*OH→*O) |
| --- | --- | --- |
| **FeTi** | **-0.64** | -0.24 |
| **FeV** | **-0.57** | -0.14 |
| FeCr | -0.07 | -0.18 |
| FeMn | 0.19 | 0.07 |
| FeFe | 0.18 | 0.00 |
| FeCo | 0.86 | 0.75 |
| FeNi | 0.77 | 0.40 |
| **FeCu** | **0.60** | 0.83 |
| **FeZr** | **-0.68** | 0.17 |
| **FeNb** | **-1.05** | -0.11 |
| FeMo | -1.06 | -1.18 |
| FeRu | 0.44 | 0.05 |
| FeRh | 0.61 | 0.19 |
| FePd | 0.91 | 0.72 |
| **FeAg** | **0.81** | 0.91 |
| **FeHf** | **-0.83** | 0.12 |
| **FeTa** | **-1.23** | -0.18 |
| **FeW** | **-1.47** | -0.77 |
| FeRe | 0.39 | -0.72 |
| FeOs | 0.39 | -0.09 |
| FeIr | 0.87 | 0.26 |
| FePt | 1.14 | 0.81 |
| FeAu | 1.13 | 0.77 |

***Table S71.*** Gibbs free energy change (Δ*G*) for *OH toward *OH-*OH versus *OH → *O + H^+^ +e^−^ of CoM. Yellow highlights represent candidate DACs that possessed *O selectivity (pink part in Figure 6a) and potentially followed the hybrid pathway, as shown in Figure S16.

| CoM | Δ*G*(*OH→*OHOH) | Δ*G*(*OH→*O) |
| --- | --- | --- |
| **CoTi** | **-0.38** | -0.12 |
| CoV | -0.18 | -0.22 |
| CoCr | 0.23 | 0.10 |
| CoMn | 0.19 | 0.03 |
| CoCo | 1.05 | 0.40 |
| **CoNi** | **0.88** | 0.94 |
| CoCu | 1.41 | 0.81 |
| **CoZr** | **-0.47** | 0.43 |
| **CoNb** | **-0.22** | 0.55 |
| CoMo | -0.02 | -0.46 |
| CoRu | 0.27 | 0.10 |
| CoRh | 1.12 | 0.46 |
| **CoPd** | **1.16** | 1.36 |
| **CoAg** | **0.72** | 0.85 |
| **CoHf** | **-0.65** | 0.37 |
| **CoTa** | **-0.32** | 0.42 |
| CoW | 0.07 | -0.51 |
| CoRe | 0.01 | -0.47 |
| CoOs | 0.22 | -0.09 |
| CoIr | 1.11 | 0.35 |
| CoPt | 1.30 | 0.77 |
| CoAu | 1.88 | 1.00 |

***Table S72.*** Gibbs free energy change (Δ*G*) for *OH toward *OH-*OH versus *OH → *O + H^+^ +e^−^ of NiM. Yellow highlights represent candidate DACs that possessed *O selectivity (pink part in Figure 6a) and potentially followed the hybrid pathway, as shown in Figure S16.

| NiM | Δ*G*(*OH→*OHOH) | Δ*G*(*OH→*O) |
| --- | --- | --- |
| **NiTi** | **0.03** | 0.37 |
| NiV | 0.17 | -0.29 |
| NiCr | 0.23 | 0.19 |
| **NiMn** | **0.48** | 0.82 |
| NiNi | 1.28 | 0.49 |
| **NiCu** | **0.92** | 0.98 |
| **NiZr** | **-0.54** | 0.19 |
| **NiNb** | **-0.74** | 0.41 |
| NiMo | -0.04 | -0.66 |
| **NiRu** | **0.29** | 0.42 |
| NiRh | 1.04 | 0.67 |
| **NiPd** | **0.88** | 1.27 |
| **NiAg** | **0.80** | 1.30 |
| **NiHf** | **-0.59** | 0.24 |
| **NiTa** | **-0.92** | 0.42 |
| NiW | -0.27 | -0.70 |
| NiRe | -0.19 | -0.73 |
| **NiOs** | **-0.06** | 0.32 |
| NiIr | 0.85 | 0.57 |
| **NiPt** | **1.11** | 1.29 |
| **NiAu** | **1.00** | 1.42 |

***Table S73.*** Gibbs free energy change (Δ*G*) for *OH toward *OH-*OH versus *OH → *O + H^+^ +e^−^ of CuM. Yellow highlights represent candidate DACs that possessed *O selectivity (pink part in Figure 6a) and potentially followed the hybrid pathway, as shown in Figure S16.

| CuM | Δ*G*(*OH→*OHOH) | Δ*G*(*OH→*O) |
| --- | --- | --- |
| **CuTi** | **-0.93** | -0.53 |
| CuV | 0.05 | -0.46 |
| CuCr | 0.33 | 0.21 |
| **CuMn** | **0.38** | 0.72 |
| **CuCu** | **1.48** | 2.00 |
| **CuZr** | **-0.53** | 0.33 |
| **CuNb** | **-0.63** | -0.42 |
| **CuMo** | **-0.63** | -0.04 |
| **CuRu** | **0.25** | 0.33 |
| CuRh | 1.05 | 1.02 |
| **CuPd** | **1.10** | 1.16 |
| **CuAg** | **1.28** | 1.65 |
| **CuHf** | **-0.72** | 0.33 |
| **CuTa** | **-0.92** | -0.44 |
| CuW | -0.88 | -0.93 |
| CuRe | -0.49 | -0.87 |
| **CuOs** | **-0.02** | 0.03 |
| **CuIr** | **0.73** | 0.94 |
| **CuPt** | **1.21** | 1.67 |
| CuAu | 1.53 | 1.44 |

***Table S74.*** Gibbs free energy change (Δ*G*) for *OH toward *OH-*OH versus *OH → *O + H^+^ +e^−^ of M_2_.

| M_2_ | Δ*G*(*OH→*OHOH) | Δ*G*(*OH→*O) |
| --- | --- | --- |
| **Ti_2_** | **-0.80** | -0.56 |
| **V_2_** | **-0.33** | -0.26 |
| **Cr_2_** | **-1.30** | -0.91 |
| Mn_2_ | -0.21 | -0.30 |
| **Zr_2_** | **-1.08** | -0.23 |
| **Nb_2_** | **-0.82** | -0.16 |
| Mo_2_ | -0.85 | -0.86 |
| Ru_2_ | 0.31 | 0.16 |
| Rh_2_ | 1.13 | 0.36 |
| **Pd_2_** | **0.75** | 0.75 |
| **Ag_2_** | **1.66** | 1.84 |
| **Hf_2_** | **-1.41** | -0.38 |
| **Ta_2_** | **-1.36** | -0.39 |
| **W_2_** | **-1.32** | -1.09 |
| **Re_2_** | **-0.34** | -0.23 |
| Os_2_ | 0.28 | -0.05 |
| Ir_2_ | 1.23 | 0.04 |
| **Pt_2_** | **0.68** | 0.73 |
| **Au_2_** | **0.58** | 0.97 |

***Table S75.*** Gibbs free energy change (Δ*G*) for *OH-*OH → *OH-*O + H^+^ +e^−^ versus *OH-*OH → *OOH + H^+^ +e^−^ of FeM.

| FeM | Δ*G*  (*OHOH →*OHO) | Δ*G*  (*OHOH →*OOH) |
| --- | --- | --- |
| FeTi | **-0.37** | 3.75 |
| FeV | **0.14** | / |
| FeCu | **0.58** | 2.53 |
| FeZr | **-0.44** | 4.24 |
| FeNb | **-0.22** | / |
| FeAg | **0.62** | 2.57 |
| FeHf | **-0.37** | 4.36 |
| FeTa | **-0.38** | 5.03 |
| FeW | **-0.18** | 5.39 |

***Table S76.*** Gibbs free energy change (Δ*G*) for *OH-*OH → *OH-*O + H^+^ +e^−^ versus *OH-*OH → *OOH + H^+^ +e^−^ of CoM.

| CoM | Δ*G*  (*OHOH →*OHO) | Δ*G*  (*OHOH →*OOH) |
| --- | --- | --- |
| CoTi | **-0.44** | / |
| CoNi | **0.54** | 2.58 |
| CoZr | **-0.38** | 3.96 |
| CoNb | **-0.34** | 3.28 |
| CoPd | **0.96** | 1.95 |
| CoAg | **1.34** | 3.07 |
| CoHf | **-0.25** | 4.21 |
| CoTa | **-0.33** | / |

***Table S77.*** Gibbs free energy change (Δ*G*) for *OH-*OH → *OH-*O + H^+^ +e^−^ versus *OH-*OH → *OOH + H^+^ +e^−^ of NiM.

| NiM | Δ*G*  (*OHOH →*OHO) | Δ*G*  (*OHOH →*OOH) |
| --- | --- | --- |
| NiTi | **-0.24** | 3.16 |
| NiMn | **0.31** | / |
| NiCu | **1.48** | 2.20 |
| NiZr | **0.07** | 3.87 |
| NiNb | **0.14** | / |
| NiRu | **0.79** | / |
| NiPd | **1.10** | 2.24 |
| NiAg | **1.81** | 2.62 |
| NiHf | **-0.01** | 3.74 |
| NiTa | **0.24** | / |
| NiOs | **0.73** | / |
| NiPt | **0.94** | 2.14 |
| NiAu | **1.74** | 1.91 |

***Table S78.*** Gibbs free energy change (Δ*G*) for *OH-*OH → *OH-*O + H^+^ +e^−^ versus *OH-*OH → *OOH + H^+^ +e^−^ of CuM.

| CuM | Δ*G*  (*OHOH →*OHO) | Δ*G*  (*OHOH →*OOH) |
| --- | --- | --- |
| CuTi | **-0.07** | 1.33 |
| CuMn | **0.63** | 2.89 |
| CuCu | **1.34** | 1.71 |
| CuZr | **0.35** | 3.95 |
| CuNb | **-0.24** | / |
| CuMo | **0.10** | / |
| CuRu | **0.94** | 2.50 |
| CuPd | **1.25** | 2.00 |
| CuAg | **1.64** | 2.10 |
| CuHf | **0.49** | 4.73 |
| CuTa | **-0.27** | / |
| CuOs | **0.73** | / |
| CuIr | **1.14** | 2.79 |
| CuPt | **1.04** | 1.90 |

***Table S79.*** Gibbs free energy change (Δ*G*) for *OH-*OH → *OH-*O + H^+^ +e^−^ versus *OH-*OH → *OOH + H^+^ +e^−^ of M_2_.

| M_2_ | Δ*G*  (*OHOH →*OHO) | Δ*G*  (*OHOH →*OOH) |
| --- | --- | --- |
| Ti_2_ | **-0.77** | 4.57 |
| V_2_ | **-0.61** | / |
| Cr_2_ | **0.14** | / |
| Zr_2_ | **-1.61** | 4.45 |
| Nb_2_ | **-0.72** | 4.22 |
| Pd_2_ | **1.44** | 2.32 |
| Ag_2_ | **1.70** | 1.78 |
| Hf_2_ | **-1.80** | 4.94 |
| Ta_2_ | **-0.79** | / |
| W_2_ | **-0.74** | / |
| Re_2_ | **-0.58** | / |
| Pt_2_ | **1.53** | 2.34 |
| Au_2_ | **1.29** | 2.50 |

***Table S80.*** Energy difference between after and before oxygen coupling, Δ*E* = *E*_Post-coupling_ − *E*_Pre-coupling_.

| M′M@NC | Δ*E*(eV) |
| --- | --- |
| CoCu@NC | -0.69 |
| CoPd@NC | -0.59 |
| NiCu@NC | -0.42 |
| NiPd@NC | -0.83 |
| NiPt@NC | -0.67 |
| CuCu@NC | -1.14 |
| CuPd@NC | -1.55 |
| CuPt@NC | -1.13 |

***Table S81.*** Free energy changes for each intermediate of NiPd@NC under different conditions.

|  | Δ*G* (eV) | | | | | |
| --- | --- | --- | --- | --- | --- | --- |
| Intermediate |  |  | *U* = 0 V vs.RHE | | *U* = 1.23 V vs.RHE | |
|  | Neutral | Candle | pH = 1 | pH = 13 | pH = 1 | pH = 13 |
| *OH | 0.868 | 0.799 | 0.509 | 0.676 | 0.319 | 0.427 |
| *OH-*OH | 1.371 | 1.179 | 1.217 | 1.220 | 1.368 | 1.287 |
| *OH-*O | 2.799 | 2.403 | 2.288 | 2.042 | 2.665 | 2.471 |
| *O_2_ | 3.975 | 3.715 | 3.714 | 3.624 | 3.927 | 3.815 |

***Table S82.*** Free energy changes for each intermediate of CuPd@NC under different conditions.

|  | Δ*G* (eV) | | | | | |
| --- | --- | --- | --- | --- | --- | --- |
| Intermediate |  |  | *U* = 0 V vs.RHE | | *U* = 1.23 V vs.RHE | |
|  | Neutral | Candle | pH = 1 | pH = 13 | pH = 1 | pH = 13 |
| *OH | 1.074 | 0.756 | 0.505 | 0.564 | 0.092 | 0.396 |
| *OH-*OH | 2.003 | 1.420 | 1.342 | 1.196 | 1.270 | 1.399 |
| *OH-*O | 3.256 | 2.581 | 2.510 | 2.151 | 2.670 | 2.680 |
| *O_2_ | 4.975 | 4.537 | 4.111 | 3.965 | 3.969 | 4.129 |

***Table S83.*** Free energy changes for each intermediate of CuPt@NC under different conditions.

|  | Δ*G* (eV) | | | | | |
| --- | --- | --- | --- | --- | --- | --- |
| Intermediate |  |  | *U* = 0 V vs.RHE | | *U* = 1.23 V vs.RHE | |
|  | Neutral | Candle | pH = 1 | pH = 13 | pH = 1 | pH = 13 |
| *OH | 1.081 | 0.775 | 0.523 | 0.597 | 0.088 | 0.406 |
| *OH-*OH | 2.120 | 1.558 | 1.436 | 1.306 | 1.335 | 1.481 |
| *OH-*O | 3.198 | 2.539 | 2.399 | 2.066 | 2.522 | 2.551 |
| *O_2_ | 4.908 | 4.486 | 4.089 | 3.943 | 3.962 | 4.112 |
