# Supplementary material for: Synergy of dual-atom catalysts deviated from the scaling relationship for oxygen evolution reaction
Source: Nat Commun. 2023 Jul 24;14:4449. doi: 10.1038/s41467-023-40177-1 (PMC10366111; doi:10.1038/s41467-023-40177-1)
Supplement: Supplementary file 3 — Description of Additional Supplementary Files [file 41467_2023_40177_MOESM3_ESM.pdf]

Description of Additional Supplementary Files

File Name: **Supplementary Data 1**

Description: Atomic coordinates for the computational models of electronic structure involved in Supplementary Figure 19 (NiPd@NC and OH/NiPd@NC) and Supplementary Figure 22 (CuPd@NC, OH/CuPd@NC, CuPt@NC and OH/CuPt@NC).
